# Supplementary material for: Association of relationship satisfaction with blood pressure: a cross-sectional study of older adults in rural Burkina Faso
Source: BMJ Open. 2024 Nov 12;14(11):e089374. doi: 10.1136/bmjopen-2024-089374 (PMC12185928; doi:10.1136/bmjopen-2024-089374)
Supplement: Supplementary data [file bmjopen-14-11-s001.pdf]

Section I: Sociodemographic Characteristics

A. Demographic Information, household composition and educational attainment

DEMOGRAPHIC INFORMATION		
D1	Are you the head of this household?	Yes…………..……………………………1<br>No….…………………………………..2
D2	What is your relationship to the head of this household?	Partner<br>Parent<br>Grandparent<br>Grandson or Granddaughter<br>Son or daughter<br>Aunt or uncle<br>Niece or nephew<br>Cousin<br>Tenant<br>Friend<br>Other, specify: _________________
D3	Sex. Ask if not clear:	Male……………………………………….1<br>Female……………………………………2
D4	How old are you?	__________ years
D5	How many people over the age of 18, including yourself, live in your household?	__________ people
D6	How many people under the age of 18 live in your household?	__________ people
D7	In total, how many years have you spent at school or in full-time study?	__________ years
D8	What is the highest level of education you have completed?	No Formal Schooling………….1<br>Less than Primary……..………..2<br>Primary complete……..……….3<br>Some secondary ……..………..4<br>Secondary complete…..……..5<br>High School complete…..……6<br>College/University………………7
D9	What is your marital status?	Never Married……………………….1<br>Currently Married…..………………2<br>Separated………………………………3<br>Divorced…………………………………4<br>Widowed………………………………..5<br>Cohabitating……………………………6<br>Refused……………………………………7

B. Socioeconomic Status, Health Expenditures, and Insurance

B1. Household assets

HOUSEHOLD ASSETS		
W1	What is the main source of water drunk by members of your household?	• Eau du robinet dans le logement<br>• Eau du robinet dans la cour/concession<br>• Eau du robinet public/borne fontaine<br>• Eau du robinet chez le voisin<br>• Puits à pompe ou forage<br>• Puits protégés<br>• Puits non protégés<br>• Source protégée<br>• Source non protégée<br>• Eau de pluie<br>• Camion citerne<br>• Charette avec petite citerne/tonneau<br>• Eau de surface<br>• Eau en bouteille<br>• Autre
W2	How long does it take to get the water and return home?	• Time, in minutes
W3	What type of toilet do members of your household usually use?	• Chasse d'eau - à un système d'égout<br>• Chasse d'eau - à un système d'égout - shared<br>• Chasse d'eau - à une fosse septique<br>• Chasse d'eau - à une fosse septique - shared<br>• Chasse d'eau - à des latrines<br>• Fosses/latrines - ventilées ameliorées (VIP)<br>• Fosses/latrines - ventilées ameliorées (VIP) - shared<br>• Fosses/latrines - avec dalles<br>• Fosses/latrines - avec dalles - shared<br>• Fosses/latrines - sans dalles/trou ouvert<br>• Fosses/latrines - sans dalles/trou ouvert - shared<br>• Toilettes à compostage<br>• Toilettes à compostage - shared<br>• Pas de toilette/nature<br>• Autre<br>• Autre - shared
W4	Do you share this toilet with other households?	• Yes/No
W5	What type of fule does your household usually use for cooking?	• Gaz propane liquifié (GPL)<br>• Charbon de bois<br>• Bois<br>• Repas non préparé dans le mémage<br>• Autre
W6	Do you have a separate room that you use as a kitchen?	• Yes/No
W7	How many bedrooms does this household have?	• Number
W8	Does your household own livestock, herds, other farm animals or poultry?	• Yes/No, if N, skip next questions if No

W9	Which of the following animals does your household own:	• Yes/No
W10	Vaches laitières ou taureaux ?	• Yes/No
W11	Autre bétail ?	• Yes/No
W12	Chevaux, ânes ou mules ?	• Yes/No
W13	Chèvres ?	• Yes/No
W14	Moutons ?	• Yes/No
W15	Poulets ou autre volaille ?	• Yes/No
W16	Does anyone in your household own farmland?	• Yes/No, skip next question if No
W17	How many hectares of farmland do household members have?	• Number
	In this household, doyou have :	
W18	L'électricité ?	• Yes/No
W19	Un poste radio ?	• Yes/No
W20	Une télévision ?	• Yes/No
W21	Un téléphone fixe ?	• Yes/No
W22	Un ordinateur ?	• Yes/No
W23	Un réfrigérateur ?	• Yes/No
W24	Un table?	• Yes/No
W25	Des chaises?	• Yes/No
W26	Une Armoire/bibliothèque?	• Yes/No
	Does a member of this household own :	
W27	Une montre ?	• Yes/No
W28	Un téléphone portable ?	• Yes/No
W29	If yes, how many mobile phones in your household are cur-rently in use?	• Number
W30	How many of the mobile phones currently in use in your household are “smartphones”?	• Number
W31	If so, do you pay for mobile data for any of these phones?	• Yes/No
W32	Une bicyclette ?	• Yes/No
W33	Une motocyclette ou un scooter ?	• Yes/No
W34	Une charrette tirée par un animal ?	• Yes/No
W35	Une voiture ou une camionnette ?	• Yes/No
W37	Does any household member have a bank account ?	• Yes/No

C. Frailty (For disability, see WHODAS 2.0 above)

FRIED FRAILTY + FALLS		
F1	Have you or those close to you noticed that you have lost weight or become thinner over the last year?	Yes…………………………………………1<br>No…………………………………………2<br>(IF NO, SKIP TO F3)
F2	How much weight have you lost (in kg)?	1. Provide in kilograms
F3	Does your health now limit the kinds or amounts of vigorous activities you can do, like digging, fetching water from a well or splitting firewood?	Yes, limited a lot……………………1<br>Yes, limited a little………………..2<br>No………………………………………..3
F4	How has your overall health changed in the last 12 months?	Much better……………………………..1<br>Better………………………………………2<br>The same…………………………………..3<br>Worse………………………………………4<br>Much worse……………………………5
	Please tell me how often you have felt this way during the past week:	
F5	I felt that everything I did was an effort.	Rarely (<1 days)……………………………..1<br>Some of the time (1-2 days)………….2<br>Occasionally (3-4 days)…………………3<br>Most or all of the time (5-7 days)….4
F6	I could not get going.	Rarely (<1 days)……………………………..1<br>Some of the time (1-2 days)………….2<br>Occasionally (3-4 days)…………………3<br>Most or all of the time (5-7 days)….4
	Please consider your activity during a usual week. The rest of the questions will provide extra information on sedentary behaviour.	
F7	On a usual weekday, how many hours did you spend sitting or reclining (excluding sleep)? This may include time sitting on a chair or bench, visiting friends, reading, sitting in church, sitting down to watch television.	________________________Hours<br>________________________Minutes
F8	On a usual weekend day, how many hours did you spend sitting or reclining (excluding sleep)? This may include time sitting on a chair or bench, visiting friends, reading, sitting in church, sitting down to watch television.	________________________Hours<br>________________________Minutes

Jaspert FM, *et al. BMJ Open* 2024; 14:e089374. doi: 10.1136/bmjopen-2024-089374

PHQ9 (9 questions)		
	Over the past two weeks, how often have you been bothered by any of the following problems?	
P1	Little interest or pleasure in doing things	Not at all……………………………….0<br>Several days…………………………..1<br>More than half the days…………2<br>Nearly every day……………………3
P2	Feeling down, depressed or hopeless	Not at all……………………………….0<br>Several days…………………………..1<br>More than half the days…………2<br>Nearly every day……………………3
P3	Trouble falling asleep, staying asleep, or sleeping too much	Not at all……………………………….0<br>Several days…………………………..1<br>More than half the days…………2<br>Nearly every day……………………3
P4	Feeling tired or having little energy	Not at all……………………………….0<br>Several days…………………………..1<br>More than half the days…………2<br>Nearly every day……………………3
P5	Poor appetite or overeating	Not at all……………………………….0<br>Several days…………………………..1<br>More than half the days…………2<br>Nearly every day……………………3
P6	Feeling bad about yourself – or that you are a failure or have let yourself or your family down	Not at all……………………………….0<br>Several days…………………………..1<br>More than half the days…………2<br>Nearly every day……………………3
P7	Trouble concentrating on things	Not at all……………………………….0<br>Several days…………………………..1<br>More than half the days…………2<br>Nearly every day……………………3
P8	Moving or speaking so slowly that other people could have noticed. Or the opposite – becoming so fidgety or restless that you have been moving around a lot more than usual	Not at all……………………………….0<br>Several days…………………………..1<br>More than half the days…………2<br>Nearly every day……………………3
P9	Thoughts that you would be better off dead, or thoughts of hurting yourself in some way	Not at all……………………………….0<br>Several days…………………………..1<br>More than half the days…………2<br>Nearly every day……………………3

		1	2	3	4	5	6	7
12	In most ways my life is close to ideal.							
13	I am satisfied with the current state of affairs in my life.							
14	If I could live my life over, I would change almost nothing.							
15	My life *does not* live up to the standards I have for a good life.							
16	I am satisfied with my life.							

E. Relationship satisfaction

IF Currently married or cohabiting: skip to Q2

1. Are you currently in a romantic relationship with someone?<br>For example: currently dating, being engaged, being married	Yes	No

IF No: Skip to next section

	Extremely unhappy	Fairly un-happy	A little unhappy	Happy	Very happy	Extremely happy
2. Please indicate the degree of happiness, all things considered, of your relationship.						
	Not at all	A little	Somewhat	Mostly	Almost completely	Completely
3. How true is the statement: "I have a warm and comfortable relationship with my partner"						
4. How rewarding is your relationship with your partner?						
5. In general, how satisfied are you with your relationship?						

Section IV: Healthcare Utilization

A. Medical History

MEDICAL HISTORY & STEPS		
MH1a	Have you ever had your blood pressure measured by a doctor or other health worker?	Yes……………………………………….1<br>No……………………………………….2<br>Don't Know………………………….3
MH1b	Have you ever been told by a doctor or other health worker that you have raised blood pressure or hypertension?	Yes……………………………………….1<br>No……………………………………….2<br>Don't Know………………………….3
MH1c	If yes, have you ever received treatment for raised blood pressure from a doctor or other health worker?	Yes……………………………………….1<br>No……………………………………….2<br>Don't Know………………………….3
MH1d	If yes, during the past two weeks, have you been taking your treatment for raised blood pressure with medications prescribed by a doctor or other health worker?	Yes……………………………………….1<br>No……………………………………….2<br>Don't Know………………………….3
MH2a	Have you ever had your blood sugar measured by a doctor or other health worker?	Yes……………………………………….1<br>No……………………………………….2<br>Don't Know………………………….3
MH2b	Have you ever been told by a doctor or other health worker that you have raised blood sugar or diabetes?	Yes……………………………………….1<br>No……………………………………….2<br>Don't Know………………………….3
MH2c	If yes, have you ever received treatment for raised blood sugar or diabetes from a doctor or other health worker?	Yes……………………………………….1<br>No……………………………………….2<br>Don't Know………………………….3
MH2d	If yes, during the past two weeks, have you been taking your treatment for raised blood sugar or diabetes with medications prescribed by a doctor or other health worker?	Yes……………………………………….1<br>No……………………………………….2<br>Don't Know………………………….3
MH3a	Have you ever had your cholesterol measured by a doctor or other health worker?	Yes……………………………………….1<br>No……………………………………….2<br>Don't Know………………………….3
MH3b	Have you ever been told by a doctor, nurse, or other health worker that you have high cholesterol?	Yes……………………………………….1<br>No……………………………………….2<br>Don't Know………………………….3
MH3c	If yes, have you ever received treatment for high cholesterol by a doctor or other health worker?	Yes……………………………………….1<br>No……………………………………….2<br>Don't Know………………………….3
MH3d	If yes, during the past two weeks, have you been taking your treatment for high cholesterol with medications prescribed by a doctor or other health worker?	Yes……………………………………….1<br>No……………………………………….2<br>Don't Know………………………….3
MH4a	Have you ever been told by a doctor or other health doctor that you have a heart disease (heart failure, heart attack, angina etc.)?	Yes……………………………………….1<br>No……………………………………….2<br>Don't Know………………………….3

Section V: Physical Battery, Measurements & Laboratory Studies

Height/Weight		
	Height	
	Weight	
	Waist Circumference	
	Women: are you pregnant?	Yes……………………………………….1<br>No………………………………………..2<br>Don't Know…………………………...3

Blood Pressure		
	Blood Pressure Measurement 1	Systolic:<br>Diastolic:
	Blood Pressure Measurement 2	Systolic:<br>Diastolic:
	Blood Pressure Measurement 3	Systolic:<br>Diastolic:

Walk speed		
	Normal walk [REFER TO SHOWCARDS] Did respondent complete the walk at usual pace?	Yes………………………………………………….1<br>No, REFUSED……………..…………………..2<br>No, cannot walk even with support...3
	Normal walk. Time at 4 meters:	_____________________Seconds
	Normal walk: Second attempt	_____________________Seconds

Grip strength		
	Have you had any surgery on your left arm, hand or wrist in the last 3 months OR arthritis or pain in your left hand or wrist?	YES...1<br>NO 2
	Have you had any surgery on your right arm, hand or wrist in the last 3 months OR arthritis or pain in your right hand or wrist?	Yes...1<br>No 2
	Which hand do you consider your dominant hand? [INSTRUCTIONS: IF A RESPONDENT IS AMBIDEXTROUS, THE HAND THAT IS USED FOR SIGNING/WRITING IS CONSIDERED THE DOMINANT HAND.]	LEFT…………………………………………..1<br>RIGHT………………………………………..2<br>USE BOTH THE SAME…………………3
	Did respondent complete the grip strength test?	Yes<br>No, refused<br>No, unable to understand instructions

Couple Satisfaction Index (CSI-4) - French Version

Avez-vous actuellement un partenaire?
- Oui
- Non

1. Dans l'ensemble, quel est le degré de bonheur de votre relation?
   0) Extrêmement malheureux
   1) Assez malheureux
   2) un peu malheureux
   3) Content
   4) Très heureux
   5) Extrêmement heureux

2. Dans quelle mesure cette affirmation est-elle vraie pour vous: "J'ai une relation chaleureuse et confortable avec mon partenaire"
   0) Pas du tout
   1) Un peu
   2) Modérément
   3) La plupart
   4) Presque totalment
   5) Complètement

3. Dans quelle mesure cette affirmation est-elle vraie pour vous: "J'ai une relation gratifiante avec mon partenaire"?
   0) Pas du tout
   1) Un peu
   2) Modérément
   3) La plupart
   4) Presque totalment
   5) Complètement

4. En général, dans quelle mesure êtes-vous satisfait de votre relation?
   0) Pas du tout
   1) Un peu
   2) Modérément
   3) La plupart
   4) Presque totalment
   5) Complètement
