# Supplementary material for: Association of relationship satisfaction with blood pressure: a cross-sectional study of older adults in rural Burkina Faso
Source: BMJ Open. 2024 Nov 12;14(11):e089374. doi: 10.1136/bmjopen-2024-089374 (PMC12185928; doi:10.1136/bmjopen-2024-089374)
Supplement: Supplementary data [file bmjopen-14-11-s002.pdf]

Table 7

Comparison of Mean Body Mass Index by Wealth Quintiles with ANOVA and Tukey-HSD[1] Results

		Mean	F-value
1st	Poorest quintile	20.6	54.8***
2nd	2nd poorest quintile	21.3	
3rd	Middle quintile	21.9	
4th	2nd richest quintile	22.1	
5th	Richest quintile	24.3	

Note. [1]Post-hoc comparisons revealed that means differed significantly between all pairs of wealth quintiles, except for the 1st and 2nd, 2nd and 3rd, and 3rd and 4th quintiles.
