# Supplementary material for: Association of relationship satisfaction with blood pressure: a cross-sectional study of older adults in rural Burkina Faso
Source: BMJ Open. 2024 Nov 12;14(11):e089374. doi: 10.1136/bmjopen-2024-089374 (PMC12185928; doi:10.1136/bmjopen-2024-089374)
Supplement: Author's manuscript [file bmjopen-2024-089374.draft_revisions.pdf]

BMJ Open is committed to open peer review. As part of this commitment we make the peer review history of every article we publish publicly available.

When an article is published we post the peer reviewers' comments and the authors' responses online. We also post the versions of the paper that were used during peer review. These are the versions that the peer review comments apply to.

The versions of the paper that follow are the versions that were submitted during the peer review process. They are not the versions of record or the final published versions. They should not be cited or distributed as the published version of this manuscript.

BMJ Open is an open access journal and the full, final, typeset and author-corrected version of record of the manuscript is available on our site with no access controls, subscription charges or pay-per-view fees (http://bmjopen.bmj.com).

If you have any questions on BMJ Open's open peer review process please email info.bmjopen@bmj.com

BMJ Open

The Association of Relationship Satisfaction with Blood Pressure in a Population-Based Sample of Older Adults in Rural Burkina Faso

Journal:	*BMJ Open*
Manuscript ID	bmjopen-2024-089374
Article Type:	Original research
Date Submitted by the Author:	28-May-2024

Complete List of Authors:	Jaspert, Felicitas; Heidelberg University, Institute of Medical Psychology, Medical Faculty and University Hospital<br>Harling, Guy; Africa Health Research Institute, Research Department of Infection and Population Health; University College London, Institute for Global Health<br>Sie, Ali; Centre de Recherche en Sante de Nouna<br>Bountogo, Mamadou; Centre de Recherche en Sante de Nouna<br>Bärnighausen, Till ; University of Heidelberg, Institute of Global Health<br>Ditzen, Beate; Heidelberg University, Institute of Medical Psychology, Medical Faculty and University Hospital<br>Fischer, Melanie; Heidelberg University, Institute of Medical Psychology, Medical Faculty and University Hospital

Keywords:	Epidemiology < TROPICAL MEDICINE, Public health < INFECTIOUS DISEASES, Cardiovascular Disease, Hypertension < CARDIOLOGY

SCHOLARONE™
Manuscripts

I, the Submitting Author has the right to grant and does grant on behalf of all authors of the Work (as defined in the below author licence), an exclusive licence and/or a non-exclusive licence for contributions from authors who are: i) UK Crown employees; ii) where BMJ has agreed a CC-BY licence shall apply, and/or iii) in accordance with the terms applicable for US Federal Government officers or employees acting as part of their official duties; on a worldwide, perpetual, irrevocable, royalty-free basis to BMJ Publishing Group Ltd ("BMJ") its licensees and where the relevant Journal is co-owned by BMJ to the co-owners of the Journal, to publish the Work in this journal and any other BMJ products and to exploit all rights, as set out in our licence.

The Submitting Author accepts and understands that any supply made under these terms is made by BMJ to the Submitting Author unless you are acting as an employee on behalf of your employer or a postgraduate student of an affiliated institution which is paying any applicable article publishing charge ("APC") for Open Access articles. Where the Submitting Author wishes to make the Work available on an Open Access basis (and intends to pay the relevant APC), the terms of reuse of such Open Access shall be governed by a Creative Commons licence – details of these licences and which Creative Commons licence will apply to this Work are set out in our licence referred to above.

Other than as permitted in any relevant BMJ Author's Self Archiving Policies, I confirm this Work has not been accepted for publication elsewhere, is not being considered for publication elsewhere and does not duplicate material already published. I confirm all authors consent to publication of this Work and authorise the granting of this licence.

For peer review only

The Association of Relationship Satisfaction with Blood Pressure in a Population-Based Sample of Older Adults in Rural Burkina Faso

For peer review only

ABSTRACT

Introduction

With increasing life expectancy and population growth in Sub-Saharan Africa, rising numbers of older adults are expected to generate a major public health challenge of increased non-communicable diseases, with cardiovascular diseases playing a central role in terms of morbidity and mortality. Research conducted in high-income settings suggests that relationship satisfaction is associated with better physical and mental health outcomes. Strong and supportive relationships may enhance cardiovascular health through various mechanisms such as reducing stress levels, improving immune function, and promoting healthy behaviors. As a first step, we examined how relationship satisfaction is associated with blood pressure (BP) – the main causal factor for cardiovascular disease – in a low-income setting.

Methods

Analyses were conducted using cross-sectional questionnaire and physical measurement data collected in 2018 via a household survey of 3,026 older adults (≥ 40 years) in rural northwestern Burkina Faso. Multiple linear regression models were calculated for systolic and diastolic BP and adjusted for age, gender, demographics/socioeconomics, and other health-related variables. Gender moderation was tested using interaction terms in multivariable analyses.

Results

A significant positive association existed between relationship satisfaction (CSI-4 score) and systolic BP (B = 0.23, 95% CI (0.02 to 0.45), p = .03) when controlling for demographics/socioeconomics. Nevertheless, this relationship lost statistical significance when additional adjustments were made for health-related variables (B = 0.21, 95% CI (-0.01 to 0.42),

For peer review only

p = .06). There was no significant association of relationship satisfaction and diastolic BP and no evidence of gender moderation.

Conclusion

In contrast to many higher-income settings, we found a positive association between relationship satisfaction and SBP in very-low-income rural Burkina Faso. Our results add to the evidence regarding the contextual nature of the association between relationship satisfaction and health, as high relationship satisfaction may not act as a health promotor in this socioeconomic context.

For peer review only

STRENGHTS AND LIMITATIONS OF THIS STUDY

- Population-based Design: The study's large, representative sample of older adults from rural Burkina Faso enhances the generalizability of the findings to similar sociodemographic settings in Sub-Saharan Africa.
- Objective Measurements: The use of standardized questionnaires and physical measurements allows for objective quantification of health parameters, ensuring comparability with other studies.
- High Reliability of the CSI-4: The Couples Satisfaction Index-4 (CSI-4) demonstrated high reliability in the sample, supporting the robustness of the relationship satisfaction measure used.
- Language Translation: The translation of the CSI-4 into a widely spoken local language during interviews improves accessibility, though it may introduce subtle differences in expression that could affect the results.
- Cross-sectional Design: The cross-sectional nature of the study limits the ability to infer causation, highlighting the need for longitudinal research to better understand the causal pathways between relationship satisfaction and blood pressure in this context.

For peer review only

INTRODUCTION

Between 2019 and 2050, Sub-Saharan Africa (SSA) will account for more than half of world population growth with a predicted population rise of one billion people (1). Meanwhile, life expectancy in SSA has steadily improved from 49.4 years in 1990 to 62.9 years in 2019 and is projected to reach 68.5 years by 2050 (1). Based on population growth and population ageing, the proportion and absolute number of older people in SSA is expected to increase. As demographics change, non-communicable diseases (NCDs), including cardiovascular diseases (CVDs), are on the rise. This is reflected in the 67% increase in total disability-adjusted life years (DALYs) associated with NCDs in SSA between 1990 and 2017 (2). In 2017, the age-standardized DALY rate due to NCDs was almost as high as the one due to communicable, maternal, neonatal, and nutritional diseases, the main burden of disease in SSA to date (2). Among the NCDs causing DALYs, CVDs account for the largest share from age 40 onwards, making them particularly impactful on the older population in SSA (2). The systemic role of CVD is emphasized by the fact that in most low- and middle-income countries, the main cause of decline in NCD mortality is due to the decline in deaths from CVD (3). It is therefore especially important to target main risk factors for CVD to address the increasing public health burden of NCDs in SSA.

Social relationships are associated with the development and progression of CVD (4,5) and comparable in effect size to established health risk factors (6). Epidemiological studies have shown that lower social support is associated with a higher mortality rate of affected individuals, especially due to CVDs (e.g. 5,7). However, to date, studies examining the impact of social support and relationships on health have been conducted mainly in high-income Western countries.

When considering sources of social support, close relationships such as marriage and romantic partnership play a key role. Comparing the effects of different social relationships on well-being, the quality of marriage is more strongly associated with health than the quality of other family and friendship relationships (8), making spousal relationships a particularly important

For peer review only

target in health promotion. Although married people generally exhibit better health than unmarried people (9), the mere fact of being in a partnership or marriage does not in itself necessarily bring benefits, but rather satisfaction and support within such a relationship (10).

Relationship satisfaction (RS) is the most important construct studied to represent relationship quality (11), as well as a core construct in basic relationship research (12). Relationship quality is measured almost exclusively by means of self-reports, thematically built on two main approaches: First, on interpersonal aspects of specific couple interactions such as communication and conflict, and second, on intrapersonal aspects such as a global, subjective evaluation of the relationship. RS tends to be assigned to the second approach, although items in established measurement scales often overlap with both (11). In Funk and Rogge's (12) evaluation of measurement scales assessing RS, self-reports focusing on the global assessment of partnership were found to have more information, higher precision, and greater explanatory power with respect to group differences, arguing for more focused measurements of RS using intrapersonal aspects. Since satisfaction measures based on global ratings of RS are less dependent on culturally determined interaction processes (such as communication norms) than measures based on ratings of specific relationship behaviors (13,14), RS is a readily applicable measure for assessing couple relationships in a non-Western context. In Africa, very little research exists specifically addressing RS or its impact (e.g. 15-17). In fact, as far as we know, our study marks the first investigation into the association between RS and cardiovascular health outcomes in African countries. Concurrent research conducted by Kurniawan *et al.* (18) within the same survey explored the association between RS and metabolic outcomes, expanding upon our inquiry. This represents an initial step in addressing the need for targeted relationship research in this socioeconomic and cultural context.

To determine the association of RS with CVD, surrogate endpoints that serve as biomarkers for CVD risk can be used as health endpoints. High blood pressure (BP), a reliable surrogate

For peer review only

endpoint and main risk factor for CVD, is most strongly evidenced as a causative factor and highly prevalent in exposure (19,20). With the shift in world's highest BP values from high- to low-income countries, SSA has become one of the regions with the highest mean BP levels and increasing prevalence of elevated BP (21). Systolic BP ranked first among modifiable risk factors for CVD burden in 2019 (22), and given the high prevalence and impact of elevated BP as the most common risk factor for developing CVD in SSA (23), elevated BP is particularly important as a key factor for CVD preventability. Many established risk factors for elevated BP such as age, high body-mass-index (BMI), a sedentary lifestyle, high salt intake, alcohol consumption and smoking have already been confirmed and studied in SSA (23,24), while data on the influence of RS on BP remain scarce or even non-existent. Given that RS is a potentially modifiable risk factor, for example via relationship education programs (25), an improved understanding of potential associations could have important implications for intervention.

Apart from the lack of research on health impacts of RS in SSA in general, there is an ongoing need to clarify possible moderating effects. Literature on health effects of relationship quality shows evidence of moderation by gender. This moderation is particularly evidenced when cardiovascular surrogate endpoints are used as outcomes and tends to indicate a stronger association of relationship quality and cardiovascular health for women than for men (9). Nevertheless, most studies conducted separate analyses for gender and did not test directly whether effects were significantly different for men and women, and found mostly small, non-significant differences, where it can be assumed that the sample size used was not sufficient to detect such small effect sizes.

Current study

The current study examined the association between RS and BP, a surrogate endpoint to future cardiovascular risk, in older people of Burkina Faso, a low-income country in Western-SSA, and the moderating role of gender in this context using a large, representative sample. In

For peer review only

this first investigation of RS and cardiovascular health in SSA, we focused on the potential health impact of RS on clinically important, biological, and modifiable CVD risk factors. Burkina Faso is a country in which two CVDs rank among the top ten causes of death in 2019, and in which high BP ranks as the fourth major risk factor for most deaths and disability (26). This reinforces the relevance of our research question in this specific country and findings can potentially suggest relevant societal and health policy directions. Based on previous research from Western countries we expected (a) that higher RS would have a protective and thus negative association with systolic and diastolic BP levels, and (b) that gender would moderate the relationship, with women showing a stronger association than men.

METHODS

Study area and population

Burkina Faso, a land-locked country of SSA, is ranking as one of the lowest-income countries in the world. In 2016, Burkina Faso was listed as the seventh last country in terms of the Human Development Index with 64.8% of the population living in severe multidimensional poverty (27). The sample used for the study was drawn from the population of the Nouna Health and Demographic Surveillance System (HDSS), located in the Kossi Province, western Burkina Faso. The sample is representative of the population of Burkina Faso with appropriate caution (28). The HDSS includes the semi-urban city of Nouna and 58 surrounding villages, a study area with a total population of ~ 107,000 people living in around 15,000 households according to the 2015 census. It is surveyed by the national health research center Centre de Recherche en Santé de Nouna (CRSN). The mostly rural study population relies primarily on farming and cattle keeping. Major ethnic groups that make up the multi-ethnic health district are Dafing, Bwaba, Mossi, Peulh and Samo. Islam and the Roman Catholic Church are the most prominent religions represented. Besides the official language French, there are several local dialects, with Dioula being the most widely spoken. Most children receive hardly any education, averaging one year of

For peer review only

schooling for women and two years for men (27). In 2018, less than half of adults (≥ 15 years) could both read and write (28). Marriage is the most common relationship status for adults in Burkina Faso. By the age of 25, 95% of women are married. 58% of the population in marital unions live monogamously, with polygamy also being widespread, concerning almost 42% of married women (30).

Procedure

The study used cross-sectional data collected as part of the CRSN Heidelberg Aging Study (CHAS), a household survey conducted in the Nouna HDSS between May and July 2018. The primary objective of CHAS was to conduct a quantitative survey to assess the health status of older adults (≥ 40 years) in rural Burkina Faso. The age-eligible population consisted of approximately 18,000 individuals from the 2015 HDSS census. With the target of 3,000 responses and the assumption of 25% loss due to mortality, mobility, or non-response, 3,998 individuals were selected using a stratified two-stage cluster random sampling approach, explained in more detail elsewhere (31). Selected individuals were asked for written informed consent, with a witness in case of illiteracy. Data collection was carried out by trained fieldworkers in the form of a structured questionnaire at the respondents' place of residence. It consisted of an interview on sociodemographic characteristics, physical and mental health, and healthcare utilization, conducted either in French or translated into Dioula. Furthermore, physical measurements and a blood sample were taken by certified research staff. Ethical approval for CHAS was granted by [redacted for blind review].

Measures

Relationship satisfaction

Considering the sociocultural background as well as the size of the sample, an applicable, brief, reliable, and valid measure of RS was aimed for. Given these requirements, the Couple Satisfaction Index (CSI-4) was selected (12). The CSI-4 is a psychometrically optimized 4-item

For peer review only

self-report scale designed to assess RS in married or cohabiting couples. Items assess RS at a global level (e.g., “In general, how satisfied are you with your relationship?”). The items are typically rated on a 7-point Likert scale for the first item (0 = extremely unhappy; 6 = perfect) and on a 6-point Likert scale for the other three items (0 = not at all; 5 = perfect). The CSI-4 was translated to French for the purpose of the CHAS survey and, like other self-report measures in the survey, was administered as an interview by research staff. In order to optimize the measure for administration in CHAS, all items were rated on a 6-point Likert scale, thus making the highest score of the first item 5 = extremely happy. Item scores are summed, with higher total scores reflecting higher levels of overall RS. The CSI-4 total score ranges from 0 to 20 (usually: 0 to 21) and is used as a continuous score. Internal consistency (Cronbach’s alpha) in the current sample was $\alpha = .84$. A CSI-4 score of 13.5 or lower has been established as a cutoff to indicate relationship distress (12). Compared to traditional RS measurements, such as the Marital Adjustment Test and the Dyadic Adjustment Scale, which have already been applied in Burkina Faso (16), the CSI-4, although much shorter, provides a large amount of information, demonstrating a high degree of precision and power (12).

Blood Pressure

Following the recommendations of the WHO STEPS Surveillance Manual (32), BP – Systolic (SBP) and Diastolic (DBP) – was measured a total of three times in the left arm after at least 15 minutes of rest in a seated position. Measurements were performed using a portable BP Monitor (Omron Series 7) at 5-minute intervals between the second and third reading. The mean systolic and diastolic values of the last two measurements were calculated to minimize random errors and ensure a more accurate estimate of BP. The mean values were included in the analyses as continuous variables. Hypertension (HT) was defined using the cutoff values set by current guidelines from the American College of Cardiology/American Heart Association (SBP $\geq$ 130 mmHg or DBP $\geq$ 80 mmHg) (33).

For peer review only

Covariates

This study accounted for age and gender, relevant demographic and socioeconomic variables as well as health related variables. Age was included as a continuous variable, gender as a binary variable with men as the reference group. Demographic and socioeconomic variables included wealth quintiles, defined using a wealth index based on important housing features and household ownership of durable assets (34) which were categorized from quintile one (poorest = reference group) to five (richest), education coded 0 (less than primary = reference group) and 1 (some = primary completed or more) and ethnicity including the five main ethnic groups and others as a reference group. Health related variables included body mass index (BMI), depression measured using the Patient Health Questionnaire for Depression (PHQ-9) and sitting hours during a usual week (Sit-h/week), an indicator of physical activity, all used as continuous variables.

Patient and public involvement statement

Patients and/or the public were not involved in the design, or conduct, or reporting, or dissemination plans of this research.

Statistical analyses

Multiple linear regression models were applied to test the association between RS and SBP as well as DBP. All models were adjusted for age and gender. The effect of RS on BP was assessed when controlling for demographic and socioeconomic characteristics (Model 1) and when additionally controlling for relevant health related variables (Model 2). The individual conditions for the application of multiple linear regression models, such as the absence of multicollinearity or normal distribution of the residuals, were checked. To investigate whether the association between RS and BP was moderated by gender, an interaction term (gender x CSI-4) was created, and multivariable linear regression models were run for SBP and DBP with all the above covariates and the interaction term. All analyses were performed using R Studio (35).

RESULTS

For peer review only

Of the 3,998 people sampled, 3,026 were willing and able to give informed consent to participate and provided baseline information on sociodemographic variables. The subsample used in our analyses included only respondents who were not pregnant at the time of the survey (N = 2,969), as pregnancy is known to be associated with significant physiological changes including BP levels (36). In the subsample, 2,254 study participants reported being in a partnership and provided information on RS. Of these, averaged SBP and DBP readings were collected from 2,222 individuals. 101 participants stated having taken BP medication in the last two weeks and were therefore not included in the analyses. Six participants with missing values for BMI and Sit-h/week, as well as one outlier with an extreme value for Sit-h/week (168h = 7x24h) were removed from the sample, giving a total subsample size of N = 2,114 participants. Summary statistics were compiled for the total subsample and for women and men individually and can be found in Table 1.

For peer review only

Table 1

Sociodemographic Characteristics of Included Participants (N = 2,114)

	Total (%)	Female (%)	Male (%)
	N = 2,114 (100)	*N* = 826 (39.07)	*N* = 1,288 (60.93)
Age mean (years)	51.7	51	52.1
Marital Status			
Never married	17 (0.80)	7 (0.85)	10 (0.78)
Currently married	1,952 (92.34)	759 (91.89)	1,193 (92.62)
Separated	11 (0.52)	5 (0.61)	6 (0.47)
Divorced	0 (0.00)	0 (0.00)	0 (0.00)
Widowed	10 (0.47)	9 (1.09)	1 (0.08)
Cohabiting	124 (5.87)	46 (5.57)	78 (6.06)
Wealth-Quintiles			
Poorest quintile	332 (15.70)	140 (16.95)	192 (14.91)
2nd poorest quintile	442 (20.91)	176 (21.31)	266 (20.65)
Middle quintile	451 (21.33)	168 (20.34)	283 (21.97)
2nd richest quintile	466 (22.04)	185 (22.40)	281 (21.82)
Richest quintile	423 (20.01)	157 (19.01)	266 (20.65)
Ethnicity			
Dafin	834 (39.45)	306 (37.05)	528 (40.99)
Bwama	623 (29.47)	263 (31.84)	360 (27.95)
Mossi	282 (13.34)	105 (12.17)	177 (13.74)
Peulh	186 (8.80)	80 (9.69)	106 (8.23)
Samo	150 (7.10)	57 (6.90)	93 (7.22)

For peer review only

Other	39 (1.84)	15 (1.82)	24 (1.86)
Language			
French	73 (3.45)	5 (0.61)	68 (5.28)
Dioula	1,857 (87.84)	742 (89.83)	1,115 (86.57)
Other	184 (8.71)	79 (9.56)	105 (8.15)
Education			
Less than primary	1,958 (92.62)	797 (96.49)	1,161 (90.14)
Some	156 (7.38)	29 (3.51)	127 (9.86)

Descriptive statistics for all study variables are reported in Table 2.

For peer review only

Table 2

Descriptive Characteristics of Included Participants (N = 2,114): Means and Standard Deviations for the Total Sample, Women and Men and T-tests Comparing Gender Group Differences

	Total		Women		Men		T-tests
	N = 2114		*N* = 826		*N* = 1288		
	Mean	SD	Mean	SD	Mean	SD	*t*
CSI-4	12.2	3.6	11.6	3.7	12.5	3.5	5.43***
SBP[a]	125.22	18.22	123.01	19.20	126.63	17.43	4.48***
DBP	80.54	10.98	79.83	10.99	80.99	10.96	2.38*
BMI[b]	22.10	3.90	22.56	4.53	21.80	3.41	-4.41***
PHQ-9	4	3.4	4.3	3.3	3.7	3.4	-4.02***
Sit-h/week	24.1	12.6	24	12.2	24.2	12.8	0.53
	N	%	N	%	N	%	
Hypertensive[c]	1140	53.93	418	50.61	722	56.06	
Stage I[d]	637	30.13	229	27.72	408	31.68	
Stage II[e]	503	23.79	189	22.88	314	24.38	

.$p < .1$. *$p < .05$. **$p < .01$. ***$p < .001$

[a] BP [mmHg]

[b] BMI [kg/m^2]

[c] SBP ≥130 mmHg or DBP ≥80 mmHg

[d] SBP 130-139 mmHg or DBP 80-89 mmHg

[e] SBP ≥140 mmHg or DBP ≥ 90 mmHg

For peer review only

RS and SBP were significantly but weakly positively correlated, $r(2112) = .05$, $p = .024$, while RS and DBP were not significantly correlated, $r(2112) = .04$, $p = .056$. The results of the multiple linear regression analyses are presented in Table 3 (SBP) and Table 4 (DBP).

For peer review only

Table 3

Results of Regression Models Estimating the Association Between Relationship Satisfaction and Systolic Blood Pressure, Controlling for Demographic and Socioeconomic Characteristics (Model 1) and Additionally Relevant Health Related Variables (Model 2)

	Model 1			Model 2		
	Estimate (*B*)	95% CI	*p*	Estimate (*B*)	95% CI	*p*
CSI-4	0.23	[0.02; 0.45]	.03	0.21	[-0.01; 0.42]	.06
Age (years)	0.42	[0.34; 0.50]	<.001	0.43	[0.34; 0.51]	<.001
Female (vs male)	-2.83	[-4.41; -1.26]	<.001	-3.60	[-5.17; -2.03]	<.001
Household wealth (vs poorest quintile)						
2nd poorest quintile	0.22	[-2.33; 2.77]	.87	-0.09	[-2.61; 2.43]	.95
Middle quintile	1.09	[-1.47; 3.65]	.40	0.27	[-2.28; 2.82]	.84
2nd richest quintile	2.36	[-0.22; 4.94]	.07	1.55	[-1.02; 4.11]	.24
Richest quintile	2.03	[-0.67; 4.73]	.14	-0.29	[-3.04; 2.46]	.83
Education (vs no education)	1.76	[-1.28; 4.81]	.26	1.02	[-2.00; 4.04]	.51

For peer review only

Ethnicity						
(vs Other ethnicities)						
Bwaba	-1.34	[-7.13; 4.46]	.65	-0.87	[-6.60; 4.86]	.77
Dafin	-2.95	[-8.69; 2.80]	.31	-2.36	[-8.04; 3.32]	.42
Mossi	-4.59	[-10.58; 1.41]	.13	-4.11	[-10.04;1.82]	.17
Peulh	-5.84	[-12.02; 0.35]	.06	-4.08	[-10.22; 2.05]	.19
Semo	-1.98	[-8.26; 4.30]	.54	-1.77	[-7.99; 4.44]	.58
Body mass index				0.75	[0.54; 0.95]	<.001
PHQ-9				0.15	[-0.08; 0.39]	.20
Sitting hours per week				0.05	[-0.01; 0.12]	.08

Note. CSI-4 = Couple Satisfaction Index; PHQ-9 = Patient Health Questionnaire 9.

For peer review only

Table 4

Results of Regression Models Estimating the Association Between Relationship Satisfaction and Diastolic Blood Pressure, Controlling for Demographic and Socioeconomic Characteristics (Model 1) and Additionally Relevant Health Related Variables (Model 2)

	Model 1			Model 2		
	Estimate (*B*)	95% CI	*p*	Estimate (*B*)	95% CI	*p*
CSI-4	0.09	[-0.04; 0.22]	.18	0.07	[-0.06; 0.21]	.28
Age (years)	0.03	[-0.02; 0.08]	.21	0.04	[-0.01; 0.10]	.12
Female (vs male)	-0.89	[-1.87; 0.08]	.07	-1.33	[-2.30; -0.36]	.01
Household wealth (vs poorest quintile)						
2nd poorest quintile	-0.57	[-2.15; 1.00]	.47	-0.77	[-2.33; 0.79]	.34
Middle quintile	0.47	[-1.11; 2.05]	.56	-0.04	[-1.61; 1.54]	.96
2nd richest quintile	0.56	[-1.03; 2.15]	.49	0.06	[-1.52; 1.65]	.94
Richest quintile	1.94	[0.27; 3.61]	.02	0.56	[-1.14; 2.26]	.52

For peer review only

Education (vs no education)	1.93	[0.05; 3.81]	.04	1.48	[-0.39; 3.35]	.12
Ethnicity						
(vs Other ethnicities)						
Bwaba	-2.15	[-5.73; 1.43]	.24	-1.87	[-5.41; 1.68]	.30
Dafin	-2.62	[-6.17; 0.92]	.15	-2.26	[-5.77; 1.26]	.21
Mossi	-2.94	[-6.64; 0.76]	.12	-2.65	[-6.32; 1.02]	.16
Peulh	-3.63	[-7.45; 0.19]	.06	-2.57	[-6.37; 1.22]	.18
Semo	-2.64	[-6.52; 1.24]	.18	-2.49	[-6.33; 1.36]	.20
Body mass index				0.44	[0.31; 0.57]	<.001
PHQ-9				0.06	[-0.08; 0.21]	.40
Sitting hours per week				0.03	[-0.01; 0.06]	.17

Note. CSI-4 = Couple Satisfaction Index; PHQ-9 = Patient Health Questionnaire 9.

For peer review only

Systolic blood pressure

RS was significantly and positively (*B* = 0.23; 95% CI (0.02 to 0.45); *p* = .03) associated with SBP in Model 1 which controlled for demographic and socioeconomic variables, indicating that individuals who were more satisfied in their relationship had slightly higher SBP values. When relevant health parameters were added to the model (Model 2), the association between RS and SBP was no longer significant (*B* = 0.21; 95% CI (-0.01 to 0.42); *p* = .06). In both models for SBP, age and gender were significant covariates. Age was positively associated with SBP in Model 1 and 2, and women had lower SBP compared to men. In Model 2, BMI was also significantly and positively associated with SBP. The adjusted R^2 was .06 for Model 1 and .08 for Model 2, indicating that the predictors used in each model jointly explained about 6% and 8% of the variance in SBP.

Diastolic blood pressure

No significant association was found between RS and DBP, neither in Model 1 (*B* = 0.09; 95% CI (-0.04 to 0.22); *p* = .18), which controlled for demographic and socioeconomic variables, nor in Model 2 (*B* = 0.07; 95% CI (-0.06 to 0.21); *p* = .28), when relevant health parameters were added. In Model 1, the wealthiest, represented by wealth quintile five, had significantly higher DBP compared to the poorest, as did the educated compared to the uneducated. In Model 2, significant differences in DBP existed between genders, with women having lower DBP compared to the reference group of men. Also, BMI was significantly and positively associated with DBP. The adjusted R^2 was .01 for Model 1 and .03 for Model 2.

Gender as a moderator

The interaction term (gender x CSI-4) was not statistically significant, neither in the linear regression model predicting SBP (*B* = 0.03; 95% CI (-0.08 to 0.47); *p* = .88), nor in the linear regression model predicting DBP (*B* = 0.01; 95% CI (-0.10 to 0.24); *p* = .92), reflecting that there was no evidence of gender differences in the association of RS with BP.

DISCUSSION

For peer review only

The results of our population-based analysis of older adults in Burkina Faso suggest that RS is not associated with DBP. However, RS was positively associated with SBP, although the association was no longer significant after controlling for additional health-related parameters such as the BMI. The correlation coefficient between RS and SBP, although small, was comparable in magnitude to previously reported, mostly small effect sizes of the association between marital quality and health outcomes (r = .07 to .21) (9) that have important implications at a population level. In our multiple regression model adjusted for demographic and socioeconomic characteristics RS was significantly and positively associated with an increase in SBP of 0.23 mmHg per unit change in the CSI-4. The effect size corresponds to roughly half of the SBP-increasing effect of one year of ageing and points into a direction that suggests, contrary to most prior findings (9), that more relationally satisfied individuals in this population have higher SBP levels. Given that the association of RS and SBP was no longer significant after controlling for additional health-related parameters, these findings should be interpreted with caution. However, this potential association of RS with *higher* cardiovascular risk warrants further attention, as, in general, RS has been shown to be associated with health benefits. Previous data from Burkina Faso have shown that RS was associated with higher health-related quality of life among postpartum women (16), also pointing in the opposite direction of our findings. At the same time, Kurniawan *et al.* (18) also observed an unexpected positive association between RS and BMI values in our study's population, contrary to their hypothesis of higher RS leading to lower levels of metabolic risk factors.

There are in fact a few relevant exceptions in the literature where better relationship quality was associated with poorer health outcome. Birditt and Antonucci (8) demonstrated a positive association between relationship quality and mortality in a representative sample of chronically ill Americans over 40 years of age. Furthermore, Birditt *et al.* (37) found that, in contrast to the traditional moderation hypotheses of relationship quality on the stress-health link, negative relationship quality reduced the negative effects of stress on BP. Both findings

For peer review only

were mainly attributed to the complexity and context-dependency of relationship quality. Indeed, there might be important moderators, such as the type of relationship, the form of support and the respective situation or life circumstances studied, such as in the context of chronic illness (8,37). As chronic diseases are highly prevalent in our study population (38), the sample's characteristic of chronic illness could provide partial explanation for the unexpected direction of our findings: In the socioeconomic and cultural context of Burkina Faso, the family is a primary resource for coping with stressors such as sickness (39), implying that family members have a significant role as caregivers. Caregiving, however, is associated with all the characteristics of a chronic stress situation, including increased BP levels (40). As reflected in the significant prediction of the onset of hypertension by spousal care (41), close family members as caregivers are particularly at risk. However, not only caregiving, but also witnessing the spouses' suffering due to physical/psychological problems or existential/spiritual distress is a direct factor influencing CVD in caregivers (42) and can be expected to be high in SSA given the high prevalence of communicable and non-communicable diseases, mental disorders, and vital threats such as malnutrition and violence (24). As a consequence, and further mediating factor, perceived stress and its origin, might play an important role: On the one hand, a high relationship quality can buffer negative effects of stress on health both directly by spousal support and indirectly by reducing stress ratings (9). On the other hand, a high RS could likely exacerbate the negative health impact of stress if the stress arises from spousal caregiving or spousal's suffering, as Monin *et al.* (42) already hypothesized. Higher RS could therefore be associated with worse cardiovascular outcomes if more relationally satisfied individuals are more attuned to their spouses' suffering and/or experience higher caregiving distress.

There also are methodological considerations to take into account when interpreting the findings. In prior studies on the association of RS and BP, researchers have used different methods to determine BP – clinical BP derived as a mean from a one-time series of

For peer review only

measurements and ambulatory BP derived as a mean from 24-hour measurements. When clinical BP, as in our analysis, was used, no association between relationship quality/satisfaction on BP was found (e.g., 37,42). Holt-Lunstad *et al.* (10), on the other hand, used ambulatory BP values and found a significant association between marital satisfaction and 24-hour SBP as well as waking SBP. The difference in the significance of the results might be due to the different precision of BP values depending on the BP measurement method used. Ambulatory BP measurements are currently considered the most accurate measurement method for determining true BP values, as readings are taken during both daily and nocturnal activities and can therefore consider the dynamic BP changes caused by routine activities and night-time BP drops (43). Since the association of RS on health outcomes seems to be situation and circumstance dependent, the use of 24-hour BP measurements in the home and everyday environment may be the more appropriate and accurate method for determining BP values and may potentially lead to greater power in detecting the association between RS and BP.

Gender differences

The association between RS and BP was not moderated by gender, which is consistent with Kurniawan *et al.* (18), who similarly found no gender differences through the utilization of an interaction term analysis. This aligns with previous findings that found no gender differences in the relationship between marital satisfaction and mortality in a general American population sample that directly tested gender differences (44). When separate analyses for gender were conducted, Robles *et al.* (9), on the other hand, reported statistically significant, larger associations between marital quality and surrogate cardiovascular endpoints in women than in men within the framework of a meta-analysis. It was discussed that the small, mostly insignificant gender differences were possibly due to too little power to measure small effect sizes or a smaller proportion of women in the sample. Both aspects might have had an influence on our study's findings as well.

Relationship satisfaction

For peer review only

This study represents one of the first investigations to examine RS in a Burkinabe population. The level of RS in our sample indicates that most participants experienced relationship distress, as about 64% (*N* = 1,345) of individuals reported CSI-4 scores below the cut-off value of 13.5. However, this is likely a slight overestimation of individuals scoring in the distressed range, given that the maximum possible score on the CSI-4 as implemented in the current study was one point lower compared to the original scale. If the cutoff was lowered by one point to 12.5, still over half of our sample, 53.7% (N = 1136), would be considered distressed in their relationships. Women reported poorer overall RS than men, according to mean CSI-4 scores. This gender difference could be due to sociocultural specific characteristics, especially gender roles and patriarchy (45), given the results of Jackson *et al.*'s (46) meta-analysis indicating that there are no differences in marital satisfaction between men and women in community-based samples.

Strengths and limitations

A significant strength of the current study is that it is large, population-based and representative of local adults from the age of 40 years onwards who are married or have a partner. Accordingly, our findings are locally valid and may also be transferable to other rural areas in Burkina Faso and other SSA countries. Using both standardized questionnaires and physical measurements, internationally established health parameters could be objectively quantified and are thus comparable to other studies. On a statistical level, the CSI-4 was highly reliable in our sample. However, its translation into the widely spoken local language during interviews, makes it susceptible to differences in expression. Also, the cross-sectional type of our study cannot demonstrate causation. Limitations that might have prevented the detection of associations between RS, DBP, and SBP after controlling for health-related factors could be in the study design: the use of clinical rather than ambulatory BP and a brief screening measure for RS (4-item CSI) rather than a longer measure that might be able to capture more nuanced

For peer review only

differences in RS (e.g., 16- or 32-item CSI). However, another reason could be that other predictors are in fact much stronger drivers for BP in the setting of Burkina Faso.

CONCLUSIONS

Our study is the first to examine the direct association of RS and BP in rural SSA using a study design that offers initial, valuable insights into the role of RS for cardiovascular risk factors in the context of low-income countries in SSA, a sociodemographic and economic setting that has been largely ignored in this research field. Our data suggest that RS contributes marginally to explaining SBP variance compared to established BP drivers, especially age and BMI (47), in Burkina Faso and interestingly in the opposite direction to what has mainly been described in other settings. These findings provide evidence for the importance of the contextuality of the relationship between RS and health. Further research is needed in the context of low-income SSA to better understand the effects of RS in this sociocultural setting and to draw general conclusions about the pathways through which RS might exert influence. To examine the direct effect of RS in SSA, future studies should address clinical outcomes such as mortality, as well as other cardiovascular surrogate endpoints such as ambulatory BP. Furthermore, the moderating role of RS on the stress-health link should be investigated further in the context of low-income countries, as it may yield interesting results regarding relevant sample characteristics such as high-prevalence chronic disease or family roles.

For peer review only

ACKNOWLEDGMENTS

We would like to express our gratitude to all the interviewers and data collectors responsible for collecting and executing the field study as well as to the participants for taking part in the survey. Additionally, we would like to thank the attendees of the [redacted for blind review] Conference of [redacted for blind review] for their interest in our research and the valuable discussions during the poster session. The presented results have already been showcased at the conference, and the feedback received has contributed to the development of this manuscript. We refer to the published abstract by [redacted for blind review].

FUNDING

This work was supported by the Alexander von Humboldt Foundation through an Alexander von Humboldt Professorship award to [redacted for blind review], which is funded by the German Federal Ministry of Education and Research. [Redacted for blind review] is supported by a fellowship from the Wellcome Trust and Royal Society (grant number 210479/Z/18/Z). [Redacted for blind review] was supported by a grant of the Baden-Wuerttemberg Ministry of Science, Research, and the Arts (Germany) and the European Social Fund. This research was funded in whole, or in part, by the Wellcome Trust (grant number 210479/Z/18/Z). For the purpose of open access, the author has applied a CC BY public copyright license to any Author Accepted Manuscript version arising from this submission. The funders had no role in study design, data collection and analysis, decision to publish, or preparation of the manuscript.

COMPETING INTERESTS

The authors declare no conflict of interest.

DATA SHARING STATEMENT

Data used in this study are not publicly available due to restrictions on data sharing imposed by the consent agreements with participants. The dataset includes entire age cohorts from specific villages, and there is a potential risk of deductive disclosure with sufficient local

For peer review only

information. Anonymized data can be obtained from the CHAS study data controllers upon signing a data use agreement that restricts onward transmission. Researchers interested in replicating the analyses or pursuing collaborative studies using CHAS data are encouraged to contact [redacted for blind review] for further information and to discuss potential collaborations.

REFLEXIVITY STATEMENT

The manuscript underwent individual review by all co-authors, ensuring constructive engagement with the reflexivity statement. Research partners who conducted on-site data collection and survey implementation collaborated with partners specialized in relationship satisfaction to develop the research question. Given the increasing prevalence of cardiovascular diseases in LMICs, investigating potential risk factors is a priority. While there is no LMIC co-author as first or last author due to the collaborative nature of the research question, which originated from two German institutes and was conducted as part of a medical doctoral thesis, LMIC co-authors played a significant role in the design and implementation of the large-scale population study as well as in the development of this manuscript. Data sharing is ensured as the study is approved by all parties including LMIC co-authors. Furthermore, an open access publication is aimed for to facilitate publication dissemination. Open access funding from the Wellcome Trust has been granted.

ETHICAL APPROVAL

Ethical approval for this study was granted by [redacted for blind review]. Prior to participation, oral assent was obtained from all village elders. Additionally, written informed consent was obtained from each participant, and in cases of illiteracy, a literate witness assisted in the consent process.

TRANSPARENCY STATEMENT

For peer review only

The lead authors affirm that this manuscript provides an honest, accurate, and transparent account of the study reported herein. No important aspects of the study have been omitted, and any discrepancies from the original study plan have been duly explained.

For peer review only

REFERENCES

1. United Nations. World Population Prospects 2019: Highlights: United Nations; 2019. https://doi.org/10.18356/13bf5476-en
2. Gouda HN, Charlson F, Sorsdahl K, *et al.* Burden of non-communicable diseases in sub-Saharan Africa, 1990–2017: results from the Global Burden of Disease Study 2017. *The Lancet Global Health* 2019;7(10):e1375-e87. https://doi.org/10.1016/S2214-109X(19)30374-2
3. Bennett JE, Stevens GA, Mathers CD, *et al.* NCD Countdown 2030: worldwide trends in non-communicable disease mortality and progress towards Sustainable Development Goal target 3.4. *The Lancet* 2018;392(10152):1072-88. https://doi.org/10.1016/S0140-6736(18)31992-5
4. Holt-Lunstad J, Smith TB, Layton JB. Social Relationships and Mortality Risk: A Meta-analytic Review. *PLOS Medicine* 2010;7(7):e1000316. https://doi.org/10.1371/journal.pmed.1000316
5. Orth-Gomér K, Rosengren A, Wilhelmsen L. Lack of social support and incidence of coronary heart disease in middle-aged Swedish men. *Psychosomatic medicin.* 1993;55(1):37-43. https://doi.org/10.1097/00006842-199301000-00007
6. House JS, Landis KR, Umberson D. Social relationships and health. *Science* 1988;241(4865):540-5. https://doi.org/10.1126/science.3399889
7. Rutledge T, Reis SE, Olson M, *et al.* Social Networks Are Associated With Lower Mortality Rates Among Women With Suspected Coronary Disease: The National Heart, Lung, and Blood Institute-Sponsored Women's Ischemia Syndrome Evaluation Study. *Psychosomatic Medicine* 2004;66(6):882-8. https://doi.org/10.1097/01.psy.0000145819.94041.52

For peer review only

8. Birditt KS, Antonucci TC. Life sustaining irritations? Relationship quality and mortality in the context of chronic illness. *Social Science & Medicine* 2008;67(8):1291-9. https://doi.org/10.1016/j.socscimed.2008.06.029.

9. Robles TF, Slatcher RB, Trombello JM, *et al.* Marital quality and health: A meta-analytic review. *Psychological Bulletin* 2014;140(1): 140–187. https://doi.org/10.1037/a0031859

10. Holt-Lunstad J, Birmingham W, Jones BQ. Is There Something Unique about Marriage? The Relative Impact of Marital Status, Relationship Quality, and Network Social Support on Ambulatory Blood Pressure and Mental Health. *Annals of Behavioral Medicine* 2008;35(2):239-44. https://doi.org/10.1007/s12160-008-9018-y

11. Fincham FD, Rogge R, Beach SRH. Relationship Satisfaction. In: Vangelisti AL, Perlman D, editors. The Cambridge Handbook of Personal Relationships. Cambridge: Cambridge University Press 2018;p. 422–36. (Cambridge Handbooks in Psychology). https://doi.org/10.1017/9781316417867.033

12. Funk JL, Rogge RD. Testing the ruler with item response theory: Increasing precision of measurement for relationship satisfaction with the Couples Satisfaction Index. *Journal of Family Psychology* 2007;21(4):572-583. https://doi.org/10.1037/0893-3200.21.4.572

13. Fonseca AL, Ye T, Curran M, *et al.* Cultural similarities and differences in relationship goals in intercultural romantic couples. *Journal of Family Issues* 2020;42(4), 813-838. https://doi.org/10.1177/0192513X20929071

14. Heyman RE, Sayers SL, Bellack AS. Global marital satisfaction versus marital adjustment: An empirical comparison of three measures. *Journal of Family Psychology* 1995;8(4):432–446. https://doi.org/10.1037/0893-3200.8.4.432

For peer review only

15. Lesch E, Engelbrecht S-K. Relationship satisfaction and gender differences in a South African farm-worker community. *South African Review of Sociology* 2011;42(1):58-77. https://doi.org/10.1080/21528586.2011.563542

16. Lokubal P, Calvert C, Cousens S, *et al.* Investigating the effect of relationship satisfaction on postpartum women's health-related quality of life in Burkina Faso: a cross-sectional analysis. *BMJ Open* 2021;11(9), e048230. https://doi.org/10.1136/bmjopen-2020-048230

17. Sodeinde KJ, Olu-Abiodun O, Bamdele EF, *et al.* A cross-sectional study of female HIV patient perspective of dyadic adjustment in HIV sero-concordant and sero-discordant couples. *Research Journal of Health Sciences* 2021;*9*(2):133-141. https://doi.org/10.4314/rejhs.v9i2.3

18. Kurniawan AL, Schretzmann J, Paramastri R, et al. Relationship satisfaction and metabolic health parameters: A cross-sectional study in Burkinabe population of older adults. BMC Public Health 2024;24:827. https://doi.org/10.1186/s12889-024-17998-w

19. Fuchs FD, Whelton PK. High blood pressure and cardiovascular disease. *Hypertension* 2020;*75*(2):285-292. https://doi.org/10.1161/hypertensionaha.119.14240

20. Rasnake CM, Trumbo PR, Heinonen TM. Surrogate endpoints and emerging surrogate endpoints for risk reduction of cardiovascular disease. *Nutrition Reviews* 2008;66(2):76-81. https://doi.org/10.1111/j.1753-4887.2007.00010.x

21. Zhou B, Bentham J, Di Cesare M, *et al.* Worldwide trends in blood pressure from 1975 to 2015: a pooled analysis of 1479 population-based measurement studies with 19·1 million participants. *The Lancet* 2017;389(10064):37-55. https://doi.org/10.1016/S0140-6736(16)31919-5

For peer review only

22. Roth GA, Mensah GA, Johnson CO, *et al.* Global Burden of Cardiovascular Diseases and Risk Factors, 1990–2019: Update From the GBD 2019 Study. *Journal of the American College of Cardiology* 2020;76(25):2982-3021. https://doi.org/10.1016/j.jacc.2020.11.010

23. Hendriks ME, Wit FW, Roos MT, *et al.* Hypertension in sub-Saharan Africa: cross-sectional surveys in four rural and urban communities. *PLoS One* 2012;7(3):e32638.

24. Bos ER, Jamison DT, Bainga F, *et al*. Disease and Mortality in Sub-Saharan Africa: *The World Bank* 2006. 410 p. https://doi.org/10.1596/978-0-8213-6397-3

25. Markman HJ, Rhoades GK. Relationship Education Research: Current Status and Future Directions. *Journal of Marital and Family Therapy* 2012;38(1):169-200. https://doi.org/10.1111/j.1752-0606.2011.00247.x

26. Vos T, Lim SS, Abbafati C, *et al.* Global burden of 369 diseases and injuries in 204 countries and territories, 1990–2019: a systematic analysis for the Global Burden of Disease Study 2019. *The Lancet* 2020;396(10258):1204-22. https://doi.org/10.1016/S0140-6736(20)30925-9

27. United Nations Development Programme. Statistical update 2018: Human development indices and indicators [online]. http://report2017.archive.s3-website-us-east-1.amazonaws.com (accessed 11.09.2023).

28. Sié A, Louis VR, Gbangou A, *et al.* The Health and Demographic Surveillance System (HDSS) in Nouna, Burkina Faso, 1993-2007. *Glob Health Action* 2010;3, 5284. https://doi.org/10.3402/gha.v3i0.5284

29. Macrotrends. *Burkina Faso literacy rate 1975-2024*. [online] https://www.macrotrends.net/countries/BFA/burkina-faso/literacy-rate (accessed 25.03.2024).

For peer review only

30. Institut National de la Statistique et de la Démographie [INSD] et ICF International. Enquête démographique et de santé et à indicateurs multiples du Burkina Faso 2010. (EDSBF-MICS IV). 2012. https://dhsprogram.com/pubs/pdf/FR256/FR256.pdf

31. Witham MD, Davies JI, Bärnighausen T, *et al.* Frailty and physical performance in the context of extreme poverty: a population-based study of older adults in rural Burkina Faso. *Wellcome Open Research* 2019;4:135. https://doi.org/10.12688/wellcomeopenres.15455.1

32. World Health Organization. Noncommunicable Diseases and Mental Health Cluster. WHO STEPS surveillance manual: the WHO STEPwise approach to chronic disease risk factor surveillance/ Noncommunicable Diseases and Mental Health. *World Health Organization* 2005. https://iris.who.int/handle/10665/43376

33. Whelton PK, Carey RM, Aronow WS, *et al.* 2017 ACC/AHA/AAPA/ABC/ACPM/AGS/APhA/ASH/ASPC/NMA/PCNA Guideline for the Prevention, Detection, Evaluation, and Management of High Blood Pressure in Adults: A Report of the American College of Cardiology/American Heart Association Task Force on Clinical Practice Guidelines. *Hypertension* 2018;71(6):e13-e115. https://doi.org/doi:10.1161/HYP.0000000000000065

34. Rutstein SO, Johnson K. The DHS wealth index. Calverton, Maryland, USA: *ORC Macro* 2004. http://dhsprogram.com/pubs/pdf/CR6/CR6.pdf

35. R Core Team. R: A language and environment for statistical computing. R Foundation for Statistical Computing, Vienna, Austria; 2020. https://www.R-project.org/

36. Sanghavi M, Rutherford JD. Cardiovascular Physiology of Pregnancy. *Circulation* 2014;130(12):1003-8. https://doi.org/10.1161/CIRCULATIONAHA.114.009029

For peer review only

37. Birditt KS, Newton N, Hope S. Implications of Marital/Partner Relationship Quality and Perceived Stress for Blood Pressure Among Older Adults. *The Journals of Gerontology: Series B* 2012;69(2):188-98. https://doi.org/10.1093/geronb/gbs123

38. Odland ML, Payne C, Witham MD, *et al.* Epidemiology of multimorbidity in conditions of extreme poverty: a population-based study of older adults in rural Burkina Faso. *BMJ Global Health* 2020;5:e002096. https://doi.org/10.1136/bmjgh-2019-002096

39. Bougma M, Rossier C. The role of family networks and social capital on women's fertility intentions in Ouagadougou, Burkina Faso. *Journal of Demographic Economics* 2022;88(2):237-55. https://doi.org/10.1017/dem.2021.18

40. Schulz R, Sherwood PR. Physical and Mental Health Effects of Family Caregiving. *AJN The American Journal of Nursing* 2008;108(9):23-7. https://doi.org/10.1097/01.NAJ.0000336406.45248.4c

41. Capistrant BD, Moon JR, Glymour MM. Spousal caregiving and incident hypertension. *American Journal of Hypertension* 2012;25(4):437-43. https://doi.org/10.1038/ajh.2011.232

42. Monin JK, Schulz R, Martire LM, *et al.* Spouses' Cardiovascular Reactivity to Their Partners' Suffering. *The Journals of Gerontology: Series B* 2010;65B(2):195-201. https://doi.org/10.1093/geronb/gbp133

43. Schutte AE, Kollias A, Stergiou GS. Blood pressure and its variability: classic and novel measurement techniques. *Nature Reviews Cardiology* 2022;1-12. https://doi.org/10.1038/s41569-022-00690-0

44. Whisman MA, Gilmour AL, Salinger JM. Marital satisfaction and mortality in the United States adult population. *Health Psychology* 2018;37(11):1041-4. https://doi.org/10.1037/hea0000677

For peer review only

45. Dobrowolska M, Groyecka-Bernard A, Sorokowski P, *et al.* Global Perspective on Marital Satisfaction. *Sustainability* 2020;12(21):8817. https://doi.org/10.3390/su12218817

46. Jackson JB, Miller RB, Oka M, *et al.* Gender Differences in Marital Satisfaction: A Meta-analysis. *Journal of Marriage and Family* 2014;76(1):105-29. https://doi.org/10.1111/jomf.12077

47. Bosu WK, Aheto JMK, Zucchelli E, *et al.* Determinants of systemic hypertension in older adults in Africa: a systematic review. *BMC Cardiovascular Disorders*. 2019;19(1):173. https://doi.org/10.1186/s12872-019-1147-7

48. [redacted for blind review] Abstract presented at the [redacted for blind review] Conference [redacted for blind review]

For peer review only

Manuscript Submission "The Association of Relationship Satisfaction with Blood Pressure in a Population-Based Sample of Older Adults in Rural Burkina Faso"

Dear Adrian Aldcroft,

Enclosed is our manuscript entitled, "The Association of Relationship Satisfaction with Blood Pressure in a Population-Based Sample of Older Adults in Rural Burkina Faso", which we are submitting for consideration for publication in BMJ Open.

Due to the novelty of the sample in relation to this specific research field, the large, population-based and representative sample size of local adults aged over 40 and the associated novelty of the results, we submit this manuscript for consideration as a research article.
The material presented in this manuscript is original and not under consideration for publication elsewhere.

Reflecting the suitability of our sample's characteristics, another research article, drawing from the CHAS dataset in Burkina Faso, has already been published in BMJ Open:
Whitaker J, Harling G, Sié A, et al. Non-fatal injuries in rural Burkina Faso amongst older adults, disease burden and health system responsiveness: a cross-sectional household survey. BMJ Open 2021;11:e045621. https://doi.org/10.1136/bmjopen-2020-045621

Additionally, concurrent research conducted by Kurniawan et al. explored the association between relationship satisfaction and metabolic outcomes within the same survey, highlighting the importance of targeted relationship research in this specific socioeconomic and cultural context:
Kurniawan AL, Schretzmann J, Paramastri R, et al. Relationship satisfaction and metabolic health parameters: A cross-sectional study in Burkinabe population of older adults. BMC Public Health 2024;24:827. https://doi.org/10.1186/s12889-024-17998-w.

Furthermore, our results have been showcased at the 37th Annual Conference of the European Health Psychology Society conference in September 2023, and the feedback received has contributed to the development of this manuscript:
Jaspert FM, Harling G, Sié A, et al. "Association of relationship satisfaction and blood pressure in rural Burkina Faso's elderly population." Abstract presented at the 37th Annual Conference of the European Health Psychology Society (EHPS 2023), September 4-8, 2023, Bremen, p.404. https://2023.ehps.net/wp-content/uploads/2023/09/EHPS_2023_Abstracts_UPLOAD.pdf

While Whitaker et al. explored non-fatal injuries, disease burden and health system responsiveness and Kurniawan et al. examined relationship satisfaction effects on metabolic health in rural Burkina Faso's elderly population, our research delves into the direct link between relationship satisfaction and blood pressure, a key cardiovascular risk factor. Cardiovascular markers were not examined in Kurniawan et al. Pioneering in rural Sub-Saharan Africa, our study provides valuable initial insights into relationship satisfaction's significance for cardiovascular risk factors within low-income Sub-Saharan African contexts. Our findings, which differ from those in high-income contexts but are somewhat consistent with those of Kurniawan et al., emphasize the nuanced nature of the associations of relationship satisfaction and health.

The authors declare no conflict of interest.

We look forward to hearing from you regarding the appropriateness of our manuscript as a research article for BMJ Open.

Sincerely,

Felicitas M. Jaspert

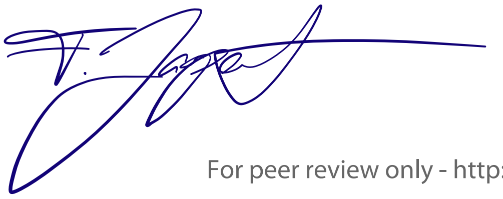

The Association of Relationship Satisfaction with Blood Pressure: A Cross-Sectional Study of Older Adults in Rural Burkina Faso

Journal:	*BMJ Open*
Manuscript ID	bmjopen-2024-089374.R1
Article Type:	Original research
Date Submitted by the Author:	09-Sep-2024
Complete List of Authors:	Jaspert, Felicitas; Heidelberg University, Institute of Medical Psychology, Medical Faculty and University Hospital<br>Harling, Guy; Africa Health Research Institute, Research Department of Infection and Population Health; University College London, Institute for Global Health<br>Sie, Ali; Centre de Recherche en Sante de Nouna<br>Bountogo, Mamadou; Centre de Recherche en Sante de Nouna<br>Bärnighausen, Till ; University of Heidelberg, Institute of Global Health<br>Ditzen, Beate; Heidelberg University, Institute of Medical Psychology, Medical Faculty and University Hospital<br>Fischer, Melanie; Heidelberg University, Institute of Medical Psychology, Medical Faculty and University Hospital
**Primary Subject Heading**:	Public health
Secondary Subject Heading:	Cardiovascular medicine, Epidemiology, Global health
Keywords:	Cardiovascular Disease, Aged, Blood Pressure, Cross-Sectional Studies, Social Support, PUBLIC HEALTH

SCHOLARONE™
Manuscripts

I, the Submitting Author has the right to grant and does grant on behalf of all authors of the Work (as defined in the below author licence), an exclusive licence and/or a non-exclusive licence for contributions from authors who are: i) UK Crown employees; ii) where BMJ has agreed a CC-BY licence shall apply, and/or iii) in accordance with the terms applicable for US Federal Government officers or employees acting as part of their official duties; on a worldwide, perpetual, irrevocable, royalty-free basis to BMJ Publishing Group Ltd ("BMJ") its licensees and where the relevant Journal is co-owned by BMJ to the co-owners of the Journal, to publish the Work in this journal and any other BMJ products and to exploit all rights, as set out in our licence.

The Submitting Author accepts and understands that any supply made under these terms is made by BMJ to the Submitting Author unless you are acting as an employee on behalf of your employer or a postgraduate student of an affiliated institution which is paying any applicable article publishing charge ("APC") for Open Access articles. Where the Submitting Author wishes to make the Work available on an Open Access basis (and intends to pay the relevant APC), the terms of reuse of such Open Access shall be governed by a Creative Commons licence – details of these licences and which Creative Commons licence will apply to this Work are set out in our licence referred to above.

Other than as permitted in any relevant BMJ Author's Self Archiving Policies, I confirm this Work has not been accepted for publication elsewhere, is not being considered for publication elsewhere and does not duplicate material already published. I confirm all authors consent to publication of this Work and authorise the granting of this licence.

For peer review only

The Association of Relationship Satisfaction with Blood Pressure: A Cross-Sectional Study of Older Adults in Rural Burkina Faso

Felicitas M. Jaspert[1], Guy Harling[2,3,4,5], MD, ScD, Ali Sié[6], MD, PhD, Mamadou Bountogo[6], MD, Till Bärnighausen[7], MD, PhD, ScD, Beate Ditzen[1], PhD and Melanie S. Fischer[1], PhD

[1] Institute of Medical Psychology, Heidelberg University Hospital, Heidelberg University, Germany

[2] Africa Health Research Institute, South Africa

[3] Institute for Global Health, University College London, UK

[4] School of Nursing & Public Health, University of KwaZulu-Natal, South Africa

[5] MRC/Wits Rural Public Health & Health Transitions Research Unit (Agincourt), University of the Witwatersrand, South Africa

[6] Centre de Recherche en Santé de Nouna, Burkina Faso

[7] Heidelberg Institute of Global Health, Heidelberg University, Germany

For peer review only

Author Note

Guy Harling https://orcid.org/0000-0001-6604-491X

Ali Sié https://orcid.org/0000-0002-3959-2520

Mamadou Bountogo https://orcid.org/0000-0001-5010-1617

Till Bärnighausen https://orcid.org/0000-0002-4182-4212

Beate Ditzen https://orcid.org/0000-0001-5853-4572

Melanie S. Fischer https://orcid.org/0000-0002-3907-1549

Melanie S. Fischer is now at the Department of Psychology, Philipps-Universität Marburg, Germany.

Correspondence concerning this article should be addressed to Melanie S. Fischer, Department of Psychology, Philipps-Universität Marburg, Gutenbergstr. 18, 35037 Marburg, Germany. E-mail: melanie.fischer@uni-marburg.de

The corresponding author attests that all listed authors meet authorship criteria and that no others meeting the criteria have been omitted.

For peer review only

ABSTRACT

Objectives

To examine the association between relationship satisfaction and blood pressure (BP) in a low-income setting and to screen for gender moderation in this context. Research conducted in high-income settings suggests that relationship satisfaction is associated with better physical and mental health outcomes.

Design

Cross-sectional study design using cross-sectional questionnaire and physical measurement data. Multiple linear regression models were calculated for systolic and diastolic BP and adjusted for age, gender, demographics/socioeconomics, and other health-related variables. Gender moderation was tested using interaction terms in multivariable analyses.

Setting

Household survey conducted in 2018 in rural northwestern Burkina Faso.

Participants

Final analysis included 2,114 participants aged over 40 who were not pregnant, reported being in a partnership, and had valid BP readings.

Main Outcome Measures

Systolic and diastolic BP levels.

Results

A significant positive association existed between relationship satisfaction (CSI-4 score) and systolic BP (B = 0.23, 95% CI (0.02 to 0.45), p = .03) when controlling for demographics/socioeconomics. Nevertheless, this relationship lost statistical significance when additional adjustments were made for health-related variables (B = 0.21, 95% CI (-0.01 to 0.42),

For peer review only

p = .06). There was no significant association of relationship satisfaction and diastolic BP and no evidence of gender moderation.

Conclusion

In contrast to many higher-income settings, we found a positive association between relationship satisfaction and systolic BP in very-low-income rural Burkina Faso. Our results add to the evidence regarding the contextual nature of the association between relationship satisfaction and health, as high relationship satisfaction may not act as a health promotor in this socioeconomic context.

For peer review only

STRENGHTS AND LIMITATIONS OF THIS STUDY

- Population-based Design: The study's large, representative sample of older adults from rural Burkina Faso enhances the generalizability of the findings to similar sociodemographic settings in Sub-Saharan Africa.
- Objective Measurements: The use of standardized questionnaires and physical measurements allows for objective quantification of health parameters, ensuring comparability with other studies.
- High Reliability of the CSI-4: The Couples Satisfaction Index-4 (CSI-4) demonstrated high reliability in the sample, supporting the robustness of the relationship satisfaction measure used.
- Measurement Constraints: The study's reliance on a brief, unidimensional measure of relationship satisfaction (CSI-4) and the use of clinical blood pressure measurements, rather than ambulatory methods, may have limited the ability to capture nuanced variations in relationship satisfaction and its potential associations with blood pressure.
- Cross-sectional Design: The cross-sectional nature of the study limits the ability to infer causation, highlighting the need for longitudinal research to better understand the causal pathways between relationship satisfaction and blood pressure in this context.

For peer review only

INTRODUCTION

Between 2019 and 2050, Sub-Saharan Africa will account for more than half of world population growth with a predicted population rise of one billion people (1). Meanwhile, life expectancy in Sub-Saharan Africa has steadily improved from 49.4 years in 1990 to 62.9 years in 2019 and is projected to reach 68.5 years by 2050 (1). Based on population growth and population ageing, the proportion and absolute number of older people in Sub-Saharan Africa is expected to increase. As demographics change, non-communicable diseases, including cardiovascular diseases (CVDs), are on the rise. This is reflected in the 67% increase in total disability-adjusted life years associated with non-communicable diseases in Sub-Saharan Africa between 1990 and 2017 (2). In 2017, the age-standardized disability-adjusted life year rate due to non-communicable diseases was almost as high as the one due to communicable, maternal, neonatal, and nutritional diseases, the main burden of disease in Sub-Saharan Africa to date (2). Among the non-communicable diseases causing disability-adjusted life years, CVDs account for the largest share from age 40 onwards, making them particularly impactful on the older population in Sub-Saharan Africa (2). The systemic role of CVD is emphasized by the fact that in most low- and middle-income countries, the main cause of decline in non-communicable disease mortality is due to the decline in deaths from CVD (3). It is therefore especially important to target main risk factors for CVD to address the increasing public health burden of non-communicable diseases in Sub-Saharan Africa.

Social relationships are associated with the development and progression of CVD (4,5) and comparable in effect size to established health risk factors (6). Epidemiological studies have shown that lower social support is associated with a higher mortality rate of affected individuals, especially due to CVDs (e.g. 5,7). However, to date, studies examining the impact of social support and relationships on health have been conducted mainly in high-income Western countries.

When considering sources of social support, close relationships such as marriage and romantic partnership play a key role. Comparing the effects of different social relationships on

For peer review only

well-being, the quality of marriage is more strongly associated with health than the quality of other family and friendship relationships (8), making spousal relationships a particularly important target in health promotion. Although married people generally exhibit better health than unmarried people, as demonstrated in a meta-analysis by Robles *et al.* (9), the mere fact of being in a partnership or marriage does not in itself necessarily bring benefits, but rather satisfaction and support within such a relationship (10).

Relationship satisfaction (RS) is the most important construct studied to represent relationship quality (11), as well as a core construct in basic relationship research (12). Relationship quality is measured almost exclusively by means of self-reports, thematically built on two main approaches: First, on interpersonal aspects of specific couple interactions such as communication and conflict, and second, on intrapersonal aspects such as a global, subjective evaluation of the relationship. RS tends to be assigned to the second approach, although items in established measurement scales often overlap with both (11). In Funk and Rogge's (12) evaluation of measurement scales assessing RS, self-reports focusing on the global assessment of partnership were found to have more information, higher precision, and greater explanatory power with respect to group differences, arguing for more focused measurements of RS using intrapersonal aspects. Since satisfaction measures based on global ratings of RS are less dependent on culturally determined interaction processes (such as communication norms) than measures based on ratings of specific relationship behaviors (13,14), RS is a readily applicable measure for assessing couple relationships in a non-Western context.

In Africa, very little research exists specifically addressing RS or its impact (e.g. 15-17). Previous research in Western countries demonstrate a significant association between high RS and better cardiovascular health, including lower BP, reduced risk of coronary heart disease, improved heart rate variability, as well as decreased incidence of myocardial infarction (9,18,19). To date, several psychophysiological pathways have been proposed to explain these associations, including

For peer review only

the promotion of better long-term health behaviors, effects on the endocrine and immune systems, and chronic stress buffering effects (9,18,20-22). Central to psychosocial research on CVD is the 'reactivity hypothesis', which posits that stress triggers cardiovascular reactivity, such as elevated blood pressure (BP) and heart rate, as well as increases in stress hormone levels and the promotion of inflammation, all of which contribute to the development and progression of CVD (18,22). Reviews of the literature show, that marital quality significantly affects cardiovascular reactivity, with negative aspects, such as hostility, leading to increased BP and heart rate in marital conflict and positive aspects, such as support, buffering stress reactivity (9,23). However, the association of RS with cardiovascular health is complex. Particularly in high-quality relationships, stress can be transmitted between partners if one partner has health problems, which emphasizes the possible detrimental effects of high relationship quality on health (23). As far as we know, our study marks the first investigation into the association between RS and cardiovascular health outcomes in African countries. Concurrent research conducted by Kurniawan *et al.* (24) within the same survey explored the association between RS and metabolic outcomes, expanding upon our inquiry. This represents an initial step in addressing the need for targeted relationship research in this socioeconomic and cultural context.

To determine the association of RS with CVD, surrogate endpoints that serve as biomarkers for CVD risk can be used as health endpoints. High BP, a reliable surrogate endpoint and main risk factor for CVD, is most strongly evidenced as a causative factor and highly prevalent in exposure (25,26). With the shift in world's highest BP values from high- to low-income countries, Sub-Saharan Africa has become one of the regions with the highest mean BP levels and increasing prevalence of elevated BP (27). Systolic BP ranked first among modifiable risk factors for CVD burden in 2019 (28) and given the high prevalence and impact of elevated BP as the most common risk factor for developing CVD in Sub-Saharan Africa (29), elevated BP is particularly important as a key factor for CVD preventability. In Sub-Saharan Africa, many established risk factors for

For peer review only

elevated BP such as age, high body-mass-index (BMI), a sedentary lifestyle, high salt intake, alcohol consumption and smoking have already been confirmed and studied (29,30), while data on the influence of RS on BP remain scarce or even non-existent. As research on composite indices distinguishing cardiovascular health levels and high normal BP in older adults is just beginning to emerge (31), and considering that RS is a potentially modifiable risk factor - such as through relationship education programs (32) - a more refined understanding of these associations could significantly inform targeted interventions.

Apart from the lack of research on health impacts of RS in Sub-Saharan Africa in general, there is an ongoing need to clarify possible moderating effects. Literature on health effects of relationship quality shows evidence of moderation by gender. This moderation is particularly evidenced when cardiovascular surrogate endpoints are used as outcomes and tends to indicate a stronger association of relationship quality and cardiovascular health for women than for men (9). Nevertheless, most studies conducted separate analyses for gender and did not test directly whether effects were significantly different for men and women, and found mostly small, non-significant differences, where it can be assumed that the sample size used was not sufficient to detect such small effect sizes.

Current study

The current study examined the association between RS and BP, a surrogate endpoint to future cardiovascular risk, in older people of Burkina Faso, a low-income country in Western-Sub-Saharan Africa, and the moderating role of gender in this context using a large, representative sample. This is the first investigation of RS and cardiovascular health in Sub-Saharan Africa focusing on the potential health impact of RS on clinically important, biological, and modifiable CVD risk factors.

Marriage practices in Burkina Faso differ significantly from Western norms. Marriage is nearly universal, with women often entering unions before age 18 through arranged marriages,

For peer review only

179 frequently involving significant age disparities. Polygamous marriages are widespread, especially
180 in rural areas, and are commonly associated with lower levels of education (33,34). While such
181 marriages are generally perceived positively by most Burkinabe women (35) and can offer benefits
182 like emotional support among co-wives, shared child-rearing responsibilities, and economic
183 advantages (36), research indicates that women in polygamous marriages exhibit significantly
184 higher rates of mental health issues and report greater difficulties in family functioning, marital
185 relationships, and overall life satisfaction, with senior wives being more affected than junior wives
186 (33,37,38). The frequency of divorces initiated by women is increasing, alongside a rise in
187 remarriages (35,39). When examining RS in this context, it's crucial to consider the unique aspects
188 of relationship formation and marital expectations: While Western marriages often emphasize
189 passionate love and intimacy, Burkinabe unions may prioritize family obligations and economic
190 considerations (39). Therefore, in arranged marriages, RS may be more closely tied to fulfilling
191 familial duties and achieving social stability rather than personal emotional
192 fulfillment. Nevertheless, an individual's global assessment of their RS, even if based on factors
193 specific to this cultural context, may still play an important role in terms of health outcomes.

194 Additionally, Burkina Faso faces a high prevalence of CVDs, with two CVDs ranking
195 among the top ten causes of death in 2019, and high BP being the fourth major risk factor for
196 mortality and disability (40). This reinforces the relevance of our research question in this specific
197 country and unique cultural and health context, potentially suggesting relevant societal and health
198 policy directions.

199 Based on previous research from Western countries we expected (a) that higher RS would
200 have a protective and thus negative association with systolic and diastolic BP levels, and (b) that
201 gender would moderate the relationship, with women showing a stronger association than men.

202 **METHODS**

203 **Study area and population**

For peer review only

204 Burkina Faso, a land-locked country of Sub-Saharan Africa, is ranking as one of the lowest-income countries in the world. In 2016, Burkina Faso was listed as the seventh last country in terms of the Human Development Index with 64.8% of the population living in severe multidimensional poverty (41). The sample used for the study was drawn from the population of the Nouna Health and Demographic Surveillance System (HDSS), located in the Kossi Province, western Burkina Faso. The sample is representative of the population of Burkina Faso with appropriate caution (42). The HDSS includes the semi-urban city of Nouna and 58 surrounding villages, a study area with a total population of ~ 107,000 people living in around 15,000 households according to the 2015 census. It is surveyed by the national health research center Centre de Recherche en Santé de Nouna (CRSN). The mostly rural study population relies primarily on farming and cattle keeping. Major ethnic groups that make up the multi-ethnic health district are Dafing, Bwaba, Mossi, Peulh and Samo. Islam and the Roman Catholic Church are the most prominent religions represented. Besides the official language French, there are several local dialects, with Dioula being the most widely spoken. Most children receive hardly any education, averaging one year of schooling for women and two years for men (41). In 2018, less than half of adults (≥ 15 years) could both read and write (42,43). Marriage is the most common relationship status for adults in Burkina Faso. By the age of 25, 95% of women are married. 58% of the population in marital unions live monogamously, with polygamy also being widespread, concerning almost 42% of married women (44).

Procedure

The study used cross-sectional data collected as part of the CRSN Heidelberg Aging Study, a household survey conducted in the Nouna HDSS between May and July 2018. The primary objective of the CRSN Heidelberg Aging Study was to conduct a quantitative survey to assess the health status of older adults (≥ 40 years) in rural Burkina Faso. The age-eligible population consisted of approximately 18,000 individuals from the 2015 HDSS census. With the target of

For peer review only

3,000 responses and the assumption of 25% loss due to mortality, mobility, or non-response, 3,998 individuals were selected using a stratified two-stage cluster random sampling approach, explained in more detail elsewhere (45). Selected individuals were asked for written informed consent, with a witness in case of illiteracy. Data collection was carried out by trained local fieldworkers in the form of a structured questionnaire at the respondents' place of residence. It consisted of an interview on sociodemographic characteristics, physical and mental health, and healthcare utilization, conducted either in French or translated into Dioula. The interviews were individual and either public or private, as chosen by the interviewees. Furthermore, physical measurements and a blood sample were taken by certified research staff. Ethical approval for the CRSN Heidelberg Aging Study was granted by the CRSN Comité d'éthique institutionnelle, the Comité national d'éthique pour la recherche en santé in Ouagadougou, and the Ethics Committee of the University of Heidelberg's Faculty of Medicine.

Measures

Relationship satisfaction

Considering the sociocultural background as well as the size of the sample, an applicable, brief, reliable, and valid measure of RS was aimed for. Given these requirements, the Couple Satisfaction Index (CSI-4) was selected (12). The CSI-4 is a psychometrically optimized 4-item self-report scale designed to assess RS in married or cohabiting couples. Items assess RS at a global level (e.g., "In general, how satisfied are you with your relationship?"). The items are typically rated on a 7-point Likert scale for the first item (0 = extremely unhappy; 6 = perfect) and on a 6-point Likert scale for the other three items (0 = not at all; 5 = perfect). The CSI-4 was translated to French for the purpose of the CRSN Heidelberg Aging Study survey and, like other self-report measures in the survey, was administered as an interview by research staff. In order to optimize the measure for administration in the CRSN Heidelberg Aging Study, all items were rated on a 6-point Likert scale, thus making the highest score of the first item 5 = extremely happy. Item

For peer review only

scores are summed, with higher total scores reflecting higher levels of overall RS. The CSI-4 total score ranges from 0 to 20 (usually: 0 to 21) and is used as a continuous score. Internal consistency (Cronbach's alpha) in the current sample was α = .84. A CSI-4 score of 13.5 or lower has been established as a cutoff to indicate relationship distress (12). Compared to traditional RS measurements, such as the Marital Adjustment Test and the Dyadic Adjustment Scale, which have already been applied in Burkina Faso (16), the CSI-4, although much shorter, provides a large amount of information, demonstrating a high degree of precision and power (12).

Blood Pressure

Following the recommendations of the WHO STEPS Surveillance Manual (46), BP – Systolic (SBP) and Diastolic (DBP) – was measured a total of three times in the left arm after at least 15 minutes of rest in a seated position. Measurements were performed using a portable BP Monitor (Omron Series 7) at 5-minute intervals between the second and third reading. The mean systolic and diastolic values of the last two measurements were calculated to minimize random errors and ensure a more accurate estimate of BP. The mean values were included in the analyses as continuous variables. Hypertension was defined using the cutoff values set by current guidelines from the American College of Cardiology/American Heart Association (SBP ≥ 130 mmHg or DBP ≥ 80 mmHg) (47).

Covariates

This study accounted for age and gender, relevant demographic and socioeconomic variables as well as health related variables. The selection of covariates was based on their established relevance in epidemiological studies and their known influence on both RS and BP, aiming to control for potential confounding factors. Age was included as a continuous variable, and gender as a binary variable with men as the reference group. Demographic and socioeconomic variables included wealth quintiles, defined using a wealth index based on important housing features and household ownership of durable assets (48), categorized from quintile one (poorest =

For peer review only

279 reference group) to five (richest). Education was coded as a binary variable where 0 represented
280 those who did not complete primary school (less than primary = reference group) and 1 represented
281 those who completed primary school or attained higher levels of education (some = primary
282 completed or more). In the context of Burkina Faso, primary education consists of six years of
283 formal schooling, and completing this level is considered a significant achievement in education
284 attainment, particularly in rural areas where access to education is limited (49). Because the
285 number of participants who completed any additional schooling past primary school was extremely
286 small (4.1%), we combined education levels into these two categories. Ethnicity was included with
287 the five main ethnic groups coded separately and others as the reference group. Health related
288 variables included body mass index (BMI), depression measured using the Patient Health
289 Questionnaire for Depression (PHQ-9) and sitting hours during a usual week (Sit-h/week), an
290 indicator of physical activity, all used as continuous variables.

291 **Patient and public involvement statement**

292 Patients and/or the public were not involved in the design, or conduct, or reporting, or
293 dissemination plans of this research.

294 **Statistical analyses**

295 To account for potential confounding effects of covariates that might influence both RS
296 and BP values, Pearson's correlation coefficients were calculated for all pairs of continuous
297 variables to assess linear relationships. For categorical variables, mean RS and BP values were
298 computed for each category. Differences between binary categories were assessed using t-tests,
299 while differences across multiple categories were evaluated using one-way ANOVA, providing
300 insight into the statistical significance of group differences.

301 Multiple linear regression models were then applied to test the association between RS and
302 SBP as well as DBP. All models were adjusted for age and gender. The effect of RS on BP was
303 assessed when controlling for demographic and socioeconomic characteristics (Model 1) and when

For peer review only

additionally controlling for relevant health related variables (Model 2). The individual conditions for the application of multiple linear regression models, such as the absence of multicollinearity or normal distribution of the residuals, were checked.

To investigate whether the association between RS and BP was moderated by gender, an interaction term (gender x CSI-4) was created, and multivariable linear regression models were run for SBP and DBP with all the above covariates and the interaction term. All analyses were performed using R Studio (50).

RESULTS

Of the 3,998 people sampled, 3,026 were willing and able to give informed consent to participate and provided baseline information on sociodemographic variables. The subsample used in our analyses included only respondents who were not pregnant at the time of the survey (N = 2,969), as pregnancy is known to be associated with significant physiological changes including BP levels (51). In the subsample, 2,254 study participants reported being in a partnership and provided information on RS. Of these, averaged SBP and DBP readings were collected from 2,222 individuals. 101 participants stated having taken BP medication in the last two weeks and were therefore not included in the analyses. Six participants with missing values for BMI and Sit-h/week, as well as one outlier with an extreme value for Sit-h/week (168h = 7x24h) were removed from the sample, giving a total subsample size of N = 2,114 participants. Summary statistics were compiled for the total subsample and for women and men individually and can be found in Table 1. To protect participant confidentiality and prevent identification of individuals in groups with small numbers, the marital statuses of separated, divorced, and widowed were combined into a single category. Descriptive statistics for all study variables are reported in Table 2.

For peer review only

Table 1
Sociodemographic Characteristics of Included Participants (N = 2,114)

	Total (%)	Female (%)	Male (%)
	N = 2,114 (100)	*N* = 826 (39.07)	*N* = 1,288 (60.93)
Age mean (years)	51.7	51	52.1
Marital Status			
Never married	17 (0.80)	7 (0.85)	10 (0.78)
Currently married	1,952 (92.34)	759 (91.89)	1,193 (92.62)
Cohabiting	124 (5.87)	46 (5.57)	78 (6.06)
Seperated, divorced or widowed [a]	21 (0.99)	14 (1.70)	7 (0.55)
Wealth-Quintiles			
Poorest quintile	332 (15.70)	140 (16.95)	192 (14.91)
2nd poorest quintile	442 (20.91)	176 (21.31)	266 (20.65)
Middle quintile	451 (21.33)	168 (20.34)	283 (21.97)
2nd richest quintile	466 (22.04)	185 (22.40)	281 (21.82)
Richest quintile	423 (20.01)	157 (19.01)	266 (20.65)
Ethnicity			
Dafin	834 (39.45)	306 (37.05)	528 (40.99)
Bwama	623 (29.47)	263 (31.84)	360 (27.95)
Mossi	282 (13.34)	105 (12.17)	177 (13.74)
Peulh	186 (8.80)	80 (9.69)	106 (8.23)
Samo	150 (7.10)	57 (6.90)	93 (7.22)
Other	39 (1.84)	15 (1.82)	24 (1.86)
Language			
French	73 (3.45)	5 (0.61)	68 (5.28)
Dioula	1,857 (87.84)	742 (89.83)	1,115 (86.57)
Other	184 (8.71)	79 (9.56)	105 (8.15)
Education			
Less than primary	1,958 (92.62)	797 (96.49)	1,161 (90.14)
Primary school or more	156 (7.38)	29 (3.51)	127 (9.86)

[a] Categories were combined in order to avoid reporting on cells with < 3 participants.

For peer review only

Table 2

Descriptive Characteristics of Included Participants (N = 2,114): Means and Standard Deviations for the Total Sample, Women and Men and T-tests Comparing Gender Group Differences

	Total		Women		Men		T-tests
	N = 2114		*N* = 826		*N* = 1288		
	Mean	SD	Mean	SD	Mean	SD	*t*
CSI-4	12.2	3.6	11.6	3.7	12.5	3.5	5.43***
SBP[a]	125.22	18.22	123.01	19.20	126.63	17.43	4.48***
DBP	80.54	10.98	79.83	10.99	80.99	10.96	2.38*
BMI[b]	22.10	3.90	22.56	4.53	21.80	3.41	-4.41***
PHQ-9	4	3.4	4.3	3.3	3.7	3.4	-4.02***
Sit-h/week	24.1	12.6	24	12.2	24.2	12.8	0.53
	N	%	N	%	N	%	
Hypertensive[c]	1140	53.93	418	50.61	722	56.06	
Stage I[d]	637	30.13	229	27.72	408	31.68	
Stage II[e]	503	23.79	189	22.88	314	24.38	

$.p < .1$. $*p < .05$. $**p < .01$. $***p < .001$

[a] BP [mmHg]

[b] BMI [kg/m^2]

[c] SBP ≥130 mmHg or DBP ≥80 mmHg

[d] SBP 130-139 mmHg or DBP 80-89 mmHg

[e] SBP ≥140 mmHg or DBP ≥ 90 mmHg

For peer review only

The results of the correlation matrix can be found in Table 3, and the statistical comparisons across categories are detailed in Table 4. RS and SBP were significantly but weakly positively correlated, $r(2112) = .05$, $p = .024$, while RS and DBP were not significantly correlated, $r(2112) = .04$, $p = .056$. The results of the multiple linear regression analyses are presented in Table 5 (SBP) and Table 6 (DBP).

Table 3

Pearson Correlation Matrix of Continuous Variables

Variables	CSI-4	SBP	DBP	Age	BMI	PHQ-9	Sit-h/week
CSI-4	1						
SBP	0.05*	1					
DBP	0.04.	0.78***	1				
Age	-0.04.	0.21***	0.02	1			
BMI	0.07**	0.12***	0.16***	-0.17***	1		
PHQ-9	-0.15***	0.06**	0.00	0.29***	-0.11***	1	
Sit-h/week	0.00	0.09***	0.04*	0.23***	-0.01	0.17***	1

.$p < .1$ and *$p < .05$ and **$p < .01$ and ***$p < .001$

For peer review only

Table 4

Comparison of Mean RS, SBP, and DBP by Categorical Variables with T-test and ANOVA Results

Variable[a]	Category	RS		SBP		DBP	
		Mean	*t*	Mean	*t*	Mean	*t*
Education	Less than primary	12.1	-2.34*	125.06	-1.42	80.31	-3.36***
	Some	12.8		127.22		83.38	
		Mean	F-value	Mean	F-value	Mean	F-value
Ethnicity	Dafin	12.4	5.21***	125.45	2.46*	80.59	1.41
	Bwama	11.7		125.96		80.70	
	Mossi	12		124.06		80.25	
	Peulh	12.9		121.49		79.22	
	Samo	12.1		127.19		80.82	
	Other	12.5		127.08		84.03	
Wealth-Quintiles	Poorest quintile	11.4	10.78***	124.88	1.30	80.00	4.49**
	2nd poorest quintile	11.9		123.78		79.34	
	Middle quintile	11.9		125.05		80.38	
	2nd richest quintile	12.5		126.18		80.55	
	Richest quintile	12.9		126.10		82.36	

[a]Gender differences are reported in Table 2.

Table 5

Results of Regression Models Estimating the Association Between Relationship Satisfaction and Systolic Blood Pressure, Controlling for Demographic and Socioeconomic Characteristics (Model 1) and Additionally Relevant Health Related Variables (Model 2)

	Model 1			Model 2		
	Estimate (*B*)	95% CI	*p*	Estimate (*B*)	95% CI	*p*
CSI-4	0.23	[0.02; 0.45]	.03	0.21	[-0.01; 0.42]	.06
Age (years)	0.42	[0.34; 0.50]	<.001	0.43	[0.34; 0.51]	<.001
Female (vs male)	-2.83	[-4.41; -1.26]	<.001	-3.60	[-5.17; -2.03]	<.001
Household wealth (vs poorest quintile)						
2nd poorest quintile	0.22	[-2.33; 2.77]	.87	-0.09	[-2.61; 2.43]	.95
Middle quintile	1.09	[-1.47; 3.65]	.40	0.27	[-2.28; 2.82]	.84
2nd richest quintile	2.36	[-0.22; 4.94]	.07	1.55	[-1.02; 4.11]	.24
Richest quintile	2.03	[-0.67; 4.73]	.14	-0.29	[-3.04; 2.46]	.83
Education (vs no education)	1.76	[-1.28; 4.81]	.26	1.02	[-2.00; 4.04]	.51
Ethnicity (vs Other ethnicities)						
Bwaba	-1.34	[-7.13; 4.46]	.65	-0.87	[-6.60; 4.86]	.77
Dafin	-2.95	[-8.69; 2.80]	.31	-2.36	[-8.04; 3.32]	.42
Mossi	-4.59	[-10.58; 1.41]	.13	-4.11	[-10.04;1.82]	.17
Peulh	-5.84	[-12.02; 0.35]	.06	-4.08	[-10.22; 2.05]	.19
Semo	-1.98	[-8.26; 4.30]	.54	-1.77	[-7.99; 4.44]	.58
Body mass index				0.75	[0.54; 0.95]	<.001
PHQ-9				0.15	[-0.08; 0.39]	.20
Sitting hours per week				0.05	[-0.01; 0.12]	.08

For peer review only

Table 6

Results of Regression Models Estimating the Association Between Relationship Satisfaction and Diastolic Blood Pressure, Controlling for Demographic and Socioeconomic Characteristics (Model 1) and Additionally Relevant Health Related Variables (Model 2)

	Model 1			Model 2		
	Estimate (*B*)	95% CI	*p*	Estimate (*B*)	95% CI	*p*
CSI-4	0.09	[-0.04; 0.22]	.18	0.07	[-0.06; 0.21]	.28
Age (years)	0.03	[-0.02; 0.08]	.21	0.04	[-0.01; 0.10]	.12
Female (vs male)	-0.89	[-1.87; 0.08]	.07	-1.33	[-2.30; -0.36]	.01
Household wealth (vs poorest quintile)						
2nd poorest quintile	-0.57	[-2.15; 1.00]	.47	-0.77	[-2.33; 0.79]	.34
Middle quintile	0.47	[-1.11; 2.05]	.56	-0.04	[-1.61; 1.54]	.96
2nd richest quintile	0.56	[-1.03; 2.15]	.49	0.06	[-1.52; 1.65]	.94
Richest quintile	1.94	[0.27; 3.61]	.02	0.56	[-1.14; 2.26]	.52
Education (vs no education)	1.93	[0.05; 3.81]	.04	1.48	[-0.39; 3.35]	.12
Ethnicity (vs Other ethnicities)						
Bwaba	-2.15	[-5.73; 1.43]	.24	-1.87	[-5.41; 1.68]	.30
Dafin	-2.62	[-6.17; 0.92]	.15	-2.26	[-5.77; 1.26]	.21
Mossi	-2.94	[-6.64; 0.76]	.12	-2.65	[-6.32; 1.02]	.16
Peulh	-3.63	[-7.45; 0.19]	.06	-2.57	[-6.37; 1.22]	.18
Semo	-2.64	[-6.52; 1.24]	.18	-2.49	[-6.33; 1.36]	.20
Body mass index				0.44	[0.31; 0.57]	<.001
PHQ-9				0.06	[-0.08; 0.21]	.40
Sitting hours per week				0.03	[-0.01; 0.06]	.17

For peer review only

334 **Systolic blood pressure**

335 RS was significantly and positively (*B* = 0.23; 95% CI (0.02 to 0.45); *p* = .03) associated
336 with SBP in Model 1 which controlled for demographic and socioeconomic variables,
337 indicating that individuals who were more satisfied in their relationship had slightly higher SBP
338 values. When relevant health parameters were added to the model (Model 2), the association
339 between RS and SBP was no longer significant (*B* = 0.21; 95% CI (-0.01 to 0.42); *p* = .06). In
340 both models for SBP, age and gender were significant covariates. Age was positively associated
341 with SBP in Model 1 and 2, and women had lower SBP compared to men. In Model 2, BMI
342 was also significantly and positively associated with SBP. The adjusted R^2 was .06 for Model
343 1 and .08 for Model 2, indicating that the predictors used in each model jointly explained about
344 6% and 8% of the variance in SBP.

345 **Diastolic blood pressure**

346 No significant association was found between RS and DBP, neither in Model 1 (*B* =
347 0.09; 95% CI (-0.04 to 0.22); *p* = .18), which controlled for demographic and socioeconomic
348 variables, nor in Model 2 (*B* = 0.07; 95% CI (-0.06 to 0.21); *p* = .28), when relevant health
349 parameters were added. In Model 1, the wealthiest, represented by wealth quintile five, had
350 significantly higher DBP compared to the poorest, as did the educated compared to the
351 uneducated. In Model 2, significant differences in DBP existed between genders, with women
352 having lower DBP compared to the reference group of men. Also, BMI was significantly and
353 positively associated with DBP. The adjusted R^2 was .01 for Model 1 and .03 for Model 2.

354 **Gender as a moderator**

355 The interaction term (gender x CSI-4) was not statistically significant, neither in the
356 linear regression model predicting SBP (*B* = 0.03; 95% CI (-0.08 to 0.47); *p* = .88), nor in the
357 linear regression model predicting DBP (*B* = 0.01; 95% CI (-0.10 to 0.24); *p* = .92), reflecting
358 that there was no evidence of gender differences in the association of RS with BP.

359 **DISCUSSION**

For peer review only

The results of our population-based analysis of older adults in Burkina Faso suggest that RS is not associated with DBP. However, RS was positively associated with SBP, although the association was no longer significant after controlling for additional health-related parameters. The correlation coefficient between RS and SBP, although small, was comparable in magnitude to previously reported, mostly small effect sizes of the association between marital quality and health outcomes (r = .07 to .21) (9) that have important implications at a population level. In our multiple regression model adjusted for demographic and socioeconomic characteristics RS was significantly and positively associated with an increase in SBP of 0.23 mmHg per unit change in the CSI-4. The effect size corresponds to roughly half of the SBP-increasing effect of one year of ageing and points into a direction that suggests, contrary to most prior findings (9), that more relationally satisfied individuals in this population have higher SBP levels.

Given that the association of RS and SBP was no longer significant after controlling for additional health-related parameters, these findings should be interpreted with caution. BMI and depressive symptoms were significantly correlated with both RS and SBP and could act as confounders in the relationship between the two. In contrast to the negative associations of BMI on RS typically observed in Western populations, BMI demonstrated a positive correlation with RS in our sample, emphasizing the importance of context-specific interpretations and the possibility of inverse associations between RS and a variety of health outcomes (24). In Burkina Faso, cultural perceptions of body size may differ, potentially viewing higher BMI as a sign of attractiveness and wealth, which could positively influence RS (24). Nevertheless, the observed associations of depressive symptoms, wealth, and education with RS were consistent with patterns observed in Western cultural contexts: depressive symptoms were associated with lower RS, while wealth and education were associated with higher RS, consistent with the often found association of mental health and socioeconomic stability with relationship quality (52).

However, potential association of RS with *higher* cardiovascular risk warrants further attention, as, in general, RS has been shown to be associated with health benefits. In contrast,

For peer review only - http://bmjopen.bmj.com/site/about/guidelines.xhtml

386 the review by Kiecolt-Glaser and Wilson (23) synthesizes empirical data demonstrating health
387 risks associated with high-satisfaction partnerships, particularly in response to a partner's
388 suffering, including elevated BP. In closer and more satisfying relationships, greater emotional
389 interdependence can amplify negative emotions if one partner is suffering and encourage a more
390 compassionate reaction, making one spouse's psychological and physiological issues more
391 impactful on the other. Kiecolt-Glaser and Wilson (23) suggest that this effect is more likely to
392 manifest among older adults with narrowing social networks and long-term shared experiences,
393 resulting in increased vulnerability to these adverse health effects. The paradoxical findings of
394 high RS being a risk factor for health particularly in distressing life situations and presumably
395 more likely for older adults could partially explain the unexpected direction of our results. In
396 Burkina Faso, the high prevalence of chronic diseases (53), mental disorders, and vital threats
397 such as malnutrition and violence (30) create an environment where the adverse effects of high
398 RS could more readily contribute to negative health outcomes. Given that the family plays a
399 central role in coping with stressors (54), a high RS could exacerbate the negative health impact
400 if the stress arises from spousal caregiving or spousal's suffering (23,55).

401 While most studies on RS have focused on monogamous relationships, understanding
402 the context of a polygamous setting is essential for interpretating the results. The complex
403 dynamics inherent in polygamous marriages, a marital type likely common in our sample, may
404 lead to a higher prevalence of ambivalent relationships. These relationships are characterized
405 by both high positive (e.g., social support) and high negative (e.g., conflict) aspects and have
406 been linked to cardiovascular risk factors such as greater 24-hour BP and greater coronary artery
407 calcification (56). According to the review by Ross *et al.* (56), positive and negative aspects of
408 close, ambivalent relationships are interacting in a way that positive aspects might not be health-
409 promoting, while negative aspects could potentially pose even greater risks to health than in
410 low-quality social ties. A high RS, presumably functioning as a positive aspect, could therefore
411 contribute to adverse health effects in the context of this heterogeneous sample. Future research

For peer review only

should address both positive and negative aspects of relationship quality separately, in order to obtain more information regarding the association with health outcomes.

Gender differences

The association between RS and BP was not moderated by gender, which is consistent with Kurniawan *et al.* (24), who similarly found no gender differences through the utilization of an interaction term analysis. This aligns with previous findings that found no gender differences in the relationship between marital satisfaction and mortality in a general American population sample that directly tested gender differences (57). When separate analyses for gender were conducted, Robles *et al.* (9), on the other hand, reported statistically significant, larger associations between marital quality and surrogate cardiovascular endpoints in women than in men. It was discussed that the small, mostly insignificant gender differences were possibly due to too little power to measure small effect sizes or a smaller proportion of women in the sample. Both aspects, as well as a possible overestimation of RS for women (as explained further below), may have influenced our study's findings as well.

Relationship satisfaction

This study represents one of the first investigations to examine RS in a Burkinabe population. The level of RS in our sample indicates that most participants experienced relationship distress, as about 64% (N = 1,345) of individuals reported CSI-4 scores below the cut-off value of 13.5. However, this is likely a slight overestimation of individuals scoring in the distressed range, given that the maximum possible score on the CSI-4 as implemented in the current study was one point lower compared to the original scale. If the cutoff was lowered by one point to 12.5, still over half of our sample, 53.7% (N = 1136), would be considered distressed in their relationships. Women reported poorer overall RS than men, according to mean CSI-4 scores. This gender difference could be due to sociocultural specific characteristics, especially gender roles and patriarchy (58), given the results of Jackson *et al.*'s (59) meta-

For peer review only

437 analysis indicating that there are no differences in marital satisfaction between men and women
438 in community-based samples.

439 **Methodological Considerations**

440 Methodological considerations must also be taken into account when interpreting our
441 findings. Robles et al. (9) noted a more consistent association between RS and structural
442 markers of cardiovascular disease compared to functional indicators like BP. Although studies
443 using BP as a surrogate marker for cardiovascular risk have mostly found beneficial effects of
444 high RS, the results have not always been uniform, nor has the context in which BP was
445 considered - including BP measurements during interactions with spouses, as the mean from
446 24-hour (ambulatory BP) or from a one-time series of BP measurements (clinical BP) (9). One
447 study, reporting findings in line with our results using a Western sample, is that of Cornelius *et*
448 *al.* (19), which showed that higher RS was associated with higher 24-hour mean SBP, and not
449 with DBP values. The authors suggest the effects of high RS possibly being time-dependent,
450 reflecting both short-term reactive BP fluctuations and long-term cardiovascular adaptations
451 that may influence outcomes in opposing ways (19). The variability in BP-related outcomes
452 results across studies may underscore the diverse pathways through which RS might influence
453 cardiovascular health.

454 **Strengths and limitations**

455 A significant strength of the current study is that it is large, population-based and
456 representative of local adults from the age of 40 years onwards who are married or have a
457 partner. Accordingly, our findings are locally valid and may also be transferable to other rural
458 areas in Burkina Faso and other Sub-Saharan African countries. Using both standardized
459 questionnaires and physical measurements, internationally established health parameters could
460 be objectively quantified and are thus comparable to other studies.

461 Nevertheless, several limitations in our study should be acknowledged. While the CSI-
462 4 was highly reliable in our sample on a statistical level, its translation into the widely spoken

For peer review only

local language during interviews makes it susceptible to differences in expression. Additionally, the unidimensional way to assess RS may not fully capture the positive and negative aspects present in the diverse range of relationships within a sample of both polygamous and monogamous unions, potentially limiting the nuanced and accurate representation of RS (60). Furthermore, the study design did not account for marital type, wife-ordering, or inter-relations, despite the possibility that the sample includes non-cohabiting couples from six villages with fewer than 50 age-eligible participants (45). Moreover, the household survey setting may have compromised privacy, potentially leading to an overestimation of RS in females, as women, whose social status is deeply connected to their husband's, often face social pressures to withhold personal feelings that could be interpreted as a challenge to the established social order (33).

The current study used clinical BP as a surrogate marker for cardiovascular risk. Since the association of RS on health outcomes seems to be situation and time dependent, the use of 24-hour BP measurements in the home and everyday environment might be more accurate for determining BP values and may potentially lead to greater power in detecting the association between RS and BP.

Limitations that might have prevented the detection of associations between RS, DBP, and SBP after controlling for health-related factors could stem from the study design: the use of clinical rather than ambulatory BP and a brief screening measure for RS rather than a longer unidimensional (e.g., 16- or 32-item CSI) or two-dimensional measure (distinct positive and negative dimensions), which might be able to capture more nuanced variations in RS. However, another reason could be that other predictors are in fact much stronger drivers for BP in the setting of Burkina Faso or because of possible confounding effects. Lastly, the cross-sectional nature of our study cannot demonstrate causation.

CONCLUSIONS

For peer review only

Our study is the first to examine the direct association of RS and BP in rural Sub-Saharan Africa using a study design that offers initial, valuable insights into the role of RS for cardiovascular risk factors in the context of low-income countries in Sub-Saharan Africa, a sociodemographic and economic setting that has been largely ignored in this research field. Our data suggest that RS contributes marginally to explaining SBP variance compared to established BP drivers, especially age and BMI (61), in Burkina Faso and interestingly in the opposite direction to what has mainly been described in other settings. These findings provide evidence for the importance of the contextuality of the relationship between RS and health. Further research is needed in the context of low-income Sub-Saharan Africa to better understand the effects of RS in this sociocultural setting and to draw general conclusions about the pathways through which RS might exert influence. To examine the direct effect of RS in Sub-Saharan Africa, future studies should address clinical outcomes such as mortality, as well as other cardiovascular surrogate endpoints such as ambulatory BP, and use measures of RS that can particularly capture the positive and negative aspects of relationships in diverse populations. Furthermore, the moderating role of RS on the stress-health link should be investigated further in the context of low-income countries, as it may yield interesting results regarding relevant sample characteristics such as high-prevalence chronic disease or family roles.

For peer review only

AUTHOR CONTRIBUTIONS

T. Bärnighausen, A. Sié and G. Harling conceived and designed the overall CRSN Heidelberg Aging Study. G. Harling and A. Sié coordinated data collection and preparation. M. Bountogo contributed to data acquisition as coordinator of field data collection. F.M. Jaspert conducted the analysis and wrote the manuscript. M.S. Fischer, B. Ditzen and T. Bärnighausen supervised the analysis and manuscript development. All authors substantively reviewed manuscripts, inputted into revisions, and approved the final manuscript.

In preparing this manuscript, ChatGPT was used to assist with refining text for clarity and accuracy, and DeepL was utilized for translation accuracy; all AI-generated content was reviewed and edited by the authors to ensure accuracy and originality.

In accordance with BMJ Open's guidelines, F.M. Jaspert and M.S. Fischer serve as guarantors for this study, accepting full responsibility for the content and conduct of the research.

For peer review only

ACKNOWLEDGMENTS

We would like to express our gratitude to all the interviewers and data collectors responsible for collecting and executing the field study as well as to the participants for taking part in the survey. Additionally, we would like to thank the attendees of the 37th Annual Conference of the European Health Psychology Society (EHPS) for their interest in our research and the valuable discussions during the poster session. The presented results have already been showcased at the conference, and the feedback received has contributed to the development of this manuscript. We refer to the published abstract by Jaspert *et al*. (62), titled 'Association of relationship satisfaction and blood pressure in rural Burkina Faso's elderly population.', presented at the EHPS in 2023, for further details.

FUNDING

This work was supported by the Alexander von Humboldt Foundation through an Alexander von Humboldt Professorship award to T. Bärnighausen, which is funded by the German Federal

For peer review only - http://bmjopen.bmj.com/site/about/guidelines.xhtml

Ministry of Education and Research. G. Harling is supported by a fellowship from the Wellcome Trust and Royal Society (grant number 210479/Z/18/Z). M. Fischer was supported by a grant of the Baden-Wuerttemberg Ministry of Science, Research, and the Arts (Germany) and the European Social Fund. This research was funded in whole, or in part, by the Wellcome Trust (grant number 210479/Z/18/Z). For the purpose of open access, the author has applied a CC BY public copyright license to any Author Accepted Manuscript version arising from this submission. The funders had no role in study design, data collection and analysis, decision to publish, or preparation of the manuscript.

COMPETING INTERESTS

The authors declare no conflict of interest.

DATA SHARING STATEMENT

Data used in this study are not publicly available due to restrictions on data sharing imposed by the consent agreements with participants. The dataset includes entire age cohorts from specific villages, and there is a potential risk of deductive disclosure with sufficient local information. Anonymized data can be obtained from the CRSN Heidelberg Aging Study data controllers upon signing a data use agreement that restricts onward transmission. Researchers interested in replicating the analyses or pursuing collaborative studies using CRSN Heidelberg Aging Study data are encouraged to contact Dr. Guy Harling (g.harling@ucl.ac.uk) for further information and to discuss potential collaborations.

REFLEXIVITY STATEMENT

The manuscript underwent individual review by all co-authors, ensuring constructive engagement with the reflexivity statement. Research partners who conducted on-site data collection and survey implementation collaborated with partners specialized in relationship satisfaction to develop the research question. Given the increasing prevalence of cardiovascular diseases in LMICs, investigating potential risk factors is a priority. While there is no LMIC co-author as first or last author due to the collaborative nature of the research question, which

For peer review only

originated from two German institutes and was conducted as part of a medical doctoral thesis, LMIC co-authors played a significant role in the design and implementation of the large-scale population study as well as in the development of this manuscript. Data sharing is ensured as the study is approved by all parties including LMIC co-authors. Furthermore, an open access publication is aimed for to facilitate publication dissemination. Open access funding from the Wellcome Trust has been granted.

ETHICAL APPROVAL

Ethical approval for this study was granted by the Ethics Commission of the Medical Faculty Heidelberg (S-120/2018), the Burkina Faso Comité d'Ethique pour la Recherche en Santé (CERS) in Ouagadougou (2018-4-045), and the Institutional Ethics Committee (CIE) of the CRSN (2018-04). Prior to participation, oral assent was obtained from all village elders. Additionally, written informed consent was obtained from each participant, and in cases of illiteracy, a literate witness assisted in the consent process.

TRANSPARENCY STATEMENT

The lead authors affirm that this manuscript provides an honest, accurate, and transparent account of the study reported herein. No important aspects of the study have been omitted, and any discrepancies from the original study plan have been duly explained.

For peer review only

For peer review only - http://bmjopen.bmj.com/site/about/guidelines.xhtml

REFERENCES

1. United Nations. World Population Prospects 2019: Highlights: United Nations; 2019. https://doi.org/10.18356/13bf5476-en

2. Gouda HN, Charlson F, Sorsdahl K, *et al.* Burden of non-communicable diseases in sub-Saharan Africa, 1990–2017: results from the Global Burden of Disease Study 2017. *The Lancet Global Health* 2019;7(10):e1375-e87. https://doi.org/10.1016/S2214-109X(19)30374-2

3. Bennett JE, Stevens GA, Mathers CD, *et al.* NCD Countdown 2030: worldwide trends in non-communicable disease mortality and progress towards Sustainable Development Goal target 3.4. *The Lancet* 2018;392(10152):1072-88. https://doi.org/10.1016/S0140-6736(18)31992-5

4. Holt-Lunstad J, Smith TB, Layton JB. Social Relationships and Mortality Risk: A Meta-analytic Review. *PLOS Medicine* 2010;7(7):e1000316. https://doi.org/10.1371/journal.pmed.1000316

5. Orth-Gomér K, Rosengren A, Wilhelmsen L. Lack of social support and incidence of coronary heart disease in middle-aged Swedish men. *Psychosomatic medicin.* 1993;55(1):37-43. https://doi.org/10.1097/00006842-199301000-00007

6. House JS, Landis KR, Umberson D. Social relationships and health. *Science* 1988;241(4865):540-5. https://doi.org/10.1126/science.3399889

7. Rutledge T, Reis SE, Olson M, *et al.* Social Networks Are Associated With Lower Mortality Rates Among Women With Suspected Coronary Disease: The National Heart, Lung, and Blood Institute-Sponsored Women's Ischemia Syndrome Evaluation Study. *Psychosomatic Medicine* 2004;66(6):882-8. https://doi.org/10.1097/01.psy.0000145819.94041.52

For peer review only

8. Birditt KS, Antonucci TC. Life sustaining irritations? Relationship quality and mortality in the context of chronic illness. *Social Science & Medicine* 2008;67(8):1291-9. https://doi.org/10.1016/j.socscimed.2008.06.029.

9. Robles TF, Slatcher RB, Trombello JM, *et al.* Marital quality and health: A meta-analytic review. *Psychological Bulletin* 2014;140(1): 140–187. https://doi.org/10.1037/a0031859

10. Holt-Lunstad J, Birmingham W, Jones BQ. Is There Something Unique about Marriage? The Relative Impact of Marital Status, Relationship Quality, and Network Social Support on Ambulatory Blood Pressure and Mental Health. *Annals of Behavioral Medicine* 2008;35(2):239-44. https://doi.org/10.1007/s12160-008-9018-y

11. Fincham FD, Rogge R, Beach SRH. Relationship Satisfaction. In: Vangelisti AL, Perlman D, editors. The Cambridge Handbook of Personal Relationships. Cambridge: Cambridge University Press 2018;p. 422–36. (Cambridge Handbooks in Psychology). https://doi.org/10.1017/9781316417867.033

12. Funk JL, Rogge RD. Testing the ruler with item response theory: Increasing precision of measurement for relationship satisfaction with the Couples Satisfaction Index. *Journal of Family Psychology* 2007;21(4):572-583. https://doi.org/10.1037/0893-3200.21.4.572

13. Fonseca AL, Ye T, Curran M, *et al.* Cultural similarities and differences in relationship goals in intercultural romantic couples. *Journal of Family Issues* 2020;42(4), 813-838. https://doi.org/10.1177/0192513X20929071

14. Heyman RE, Sayers SL, Bellack AS. Global marital satisfaction versus marital adjustment: An empirical comparison of three measures. *Journal of Family Psychology* 1995;8(4):432–446. https://doi.org/10.1037/0893-3200.8.4.432

For peer review only

15. Lesch E, Engelbrecht S-K. Relationship satisfaction and gender differences in a South African farm-worker community. *South African Review of Sociology* 2011;42(1):58-77. https://doi.org/10.1080/21528586.2011.563542

16. Lokubal P, Calvert C, Cousens S, *et al.* Investigating the effect of relationship satisfaction on postpartum women's health-related quality of life in Burkina Faso: a cross-sectional analysis. *BMJ Open* 2021;11(9), e048230. https://doi.org/10.1136/bmjopen-2020-048230

17. Sodeinde KJ, Olu-Abiodun O, Bamdele EF, *et al.* A cross-sectional study of female HIV patient perspective of dyadic adjustment in HIV sero-concordant and sero-discordant couples. *Research Journal of Health Sciences* 2021;*9*(2):133-141. https://doi.org/10.4314/rejhs.v9i2.3

18. Smith TW. Intimate relationships and coronary heart disease: implications for risk, prevention, and patient management. *Current Cardiovascular Reports* 2022;24:761-74. https://doi.org/10.1007/s11886-022-01695-4

19. Cornelius T, Birk JL, Edmondson D, *et al.* Romantic relationship satisfaction and ambulatory blood pressure during social interactions: specificity or spillover effects? *Annals of Behavioral Medicine* 2019;53:223-31. https://doi.org/10.1093/abm/kay030

20. Uchino BN, Way BM. Integrative pathways linking close family ties to health: a neurochemical perspective. *American Psychologist* 2017;72:590-600. https://doi.org/10.1037/amp0000049

21. Birditt KS, Newton N, Hope S. Implications of Marital/Partner Relationship Quality and Perceived Stress for Blood Pressure Among Older Adults. *The Journals of Gerontology: Series B* 2012;69(2):188-98. https://doi.org/10.1093/geronb/gbs123

For peer review only

22. Robles TF, Kiecolt-Glaser JK. The physiology of marriage: pathways to health. *Physiology & Behavior*. 2003;79(3):409-16. https://doi.org/10.1016/s0031-9384(03)00160-4

23. Kiecolt-Glaser JK, Wilson SJ. Lovesick: How Couples' Relationships Influence Health. *Annual Review of Clinical Psychology* 2017;13:421-43. https://doi.org/10.1146%2Fannurev-clinpsy-032816-045111

24. Kurniawan AL, Schretzmann J, Paramastri R, et al. Relationship satisfaction and metabolic health parameters: A cross-sectional study in Burkinabe population of older adults. BMC Public Health 2024;24:827. https://doi.org/10.1186/s12889-024-17998-w

25. Fuchs FD, Whelton PK. High blood pressure and cardiovascular disease. *Hypertension* 2020;*75*(2):285-292. https://doi.org/10.1161/hypertensionaha.119.14240

26. Rasnake CM, Trumbo PR, Heinonen TM. Surrogate endpoints and emerging surrogate endpoints for risk reduction of cardiovascular disease. *Nutrition Reviews* 2008;66(2):76-81. https://doi.org/10.1111/j.1753-4887.2007.00010.x

27. Zhou B, Bentham J, Di Cesare M, *et al.* Worldwide trends in blood pressure from 1975 to 2015: a pooled analysis of 1479 population-based measurement studies with 19·1 million participants. *The Lancet* 2017;389(10064):37-55. https://doi.org/10.1016/S0140-6736(16)31919-5

28. Roth GA, Mensah GA, Johnson CO, *et al.* Global Burden of Cardiovascular Diseases and Risk Factors, 1990–2019: Update From the GBD 2019 Study. *Journal of the American College of Cardiology* 2020;76(25):2982-3021. https://doi.org/10.1016/j.jacc.2020.11.010

For peer review only

29. Hendriks ME, Wit FW, Roos MT, *et al.* Hypertension in sub-Saharan Africa: cross-sectional surveys in four rural and urban communities. *PLoS One* 2012;7(3):e32638. https://doi.org/10.1371/journal.pone.0032638

30. Bos ER, Jamison DT, Bainga F, *et al*. Disease and Mortality in Sub-Saharan Africa: *The World Bank* 2006. 410 p. https://doi.org/10.1596/978-0-8213-6397-3

31. Ren Y, Cheng L, Song Y, *et al.* Ideal cardiovascular health index and high-normal blood pressure in elderly people: evidence based on real-world data. *Scientific Reports*. 2024;14(1):10166. https://doi.org/10.1038/s41598-024-60906-w

32. Markman HJ, Rhoades GK. Relationship Education Research: Current Status and Future Directions. *Journal of Marital and Family Therapy* 2012;38(1):169-200. https://doi.org/10.1111/j.1752-0606.2011.00247.x

33. Bove R, Valeggia C. Polygyny and women's health in sub-Saharan Africa. *Social Science & Medicine*. 2009;68(1):21-9. https://doi.org/10.1016/j.socscimed.2008.09.045

34. Shaiful Bahari I, Norhayati MN, Nik Hazlina NH, *et al.* Psychological impact of polygamous marriage on women and children: a systematic review and meta-analysis. *BMC Pregnancy Childbirth*. 2021;21(1):823. https://doi.org/10.1186/s12884-021-04301-7

35. Guirkinger C, Gross J, Platteau J-P. Are women emancipating? Evidence from marriage, divorce and remarriage in rural Northern Burkina Faso. *World Development* 2021;146:105512. https://doi.org/10.1016/j.worlddev.2021.105512

36. Madhavan S. Best of Friends and Worst of Enemies: Competition and Collaboration in Polygyny. *Ethnology* 2002;41(1):69-84. https://doi.org/10.2307/4153021

For peer review only

37. Shepard LD. The impact of polygamy on women's mental health: a systematic review. *Epidemiology and Psychiatric Sciences* 2013;22(1):47-62. https://doi.org/10.1017/s2045796012000121

38. Al-Krenawi A, Graham JR. A Comparison of Family Functioning, Life and Marital Satisfaction, and Mental Health of Women in Polygamous and Monogamous Marriages. *International Journal of Social Psychiatry* 2006;52(1):5-17. https://doi.org/10.1177/0020764006061245

39. Hewitt B, Churchill B. Chapter 3 - Convergence and difference: Marriage and family life from a cross-cultural perspective. In: Halford WK, van de Vijver F, editors. Cross-Cultural Family Research and Practice: Academic Press; 2020. p. 57-102. http://dx.doi.org/10.1016/B978-0-12-815493-9.00003-X

40. Vos T, Lim SS, Abbafati C, *et al.* Global burden of 369 diseases and injuries in 204 countries and territories, 1990–2019: a systematic analysis for the Global Burden of Disease Study 2019. *The Lancet* 2020;396(10258):1204-22. https://doi.org/10.1016/S0140-6736(20)30925-9

41. United Nations Development Programme. Statistical update 2018: Human development indices and indicators [online]. http://report2017.archive.s3-website-us-east-1.amazonaws.com (accessed 11.09.2023).

42. Sié A, Louis VR, Gbangou A, *et al.* The Health and Demographic Surveillance System (HDSS) in Nouna, Burkina Faso, 1993-2007. *Glob Health Action* 2010;3, 5284. https://doi.org/10.3402/gha.v3i0.5284

43. Macrotrends. *Burkina Faso literacy rate 1975-2024*. [online] https://www.macrotrends.net/countries/BFA/burkina-faso/literacy-rate (accessed 25.03.2024).

44. Institut National de la Statistique et de la Démographie [INSD] et ICF International. Enquête démographique et de santé et à indicateurs multiples du

For peer review only

Burkina Faso 2010. (EDSBF-MICS IV). 2012. https://dhsprogram.com/pubs/pdf/FR256/FR256.pdf

45. Witham MD, Davies JI, Bärnighausen T, *et al.* Frailty and physical performance in the context of extreme poverty: a population-based study of older adults in rural Burkina Faso. *Wellcome Open Research* 2019;4:135. https://doi.org/10.12688/wellcomeopenres.15455.1

46. World Health Organization. Noncommunicable Diseases and Mental Health Cluster. WHO STEPS surveillance manual: the WHO STEPwise approach to chronic disease risk factor surveillance/ Noncommunicable Diseases and Mental Health. *World Health Organization* 2005. https://iris.who.int/handle/10665/43376

47. Whelton PK, Carey RM, Aronow WS, *et al.* 2017 ACC/AHA/AAPA/ABC/ACPM/AGS/APhA/ASH/ASPC/NMA/PCNA Guideline for the Prevention, Detection, Evaluation, and Management of High Blood Pressure in Adults: A Report of the American College of Cardiology/American Heart Association Task Force on Clinical Practice Guidelines. *Hypertension* 2018;71(6):e13-e115. https://doi.org/doi:10.1161/HYP.0000000000000065

48. Rutstein SO, Johnson K. The DHS wealth index. Calverton, Maryland, USA: *ORC Macro* 2004. http://dhsprogram.com/pubs/pdf/CR6/CR6.pdf

49. Werner LK, Jabbarian J, Kagoné M, *et al.* "Because at school, you can become somebody" – The perceived health and economic returns on secondary schooling in rural Burkina Faso. *PLoS One* 2019;14:e0226911. https://doi.org/10.1371/journal.pone.0226911

50. R Core Team. R: A language and environment for statistical computing. R Foundation for Statistical Computing, Vienna, Austria; 2020. https://www.R-project.org/

51. Sanghavi M, Rutherford JD. Cardiovascular Physiology of Pregnancy. *Circulation* 2014;130(12):1003-8. https://doi.org/10.1161/CIRCULATIONAHA.114.009029

52. Rashidi Fakari F, Doulabi MA, Mahmoodi Z. Predict marital satisfaction based on the variables of socioeconomic status (SES) and social support, mediated by mental health, in women of reproductive age: Path analysis model. *Brain and Behavior* 2022;12(3):e2482. https://doi.org/10.1002/brb3.2482

53. Odland ML, Payne C, Witham MD, *et al.* Epidemiology of multimorbidity in conditions of extreme poverty: a population-based study of older adults in rural Burkina Faso. *BMJ Global Health* 2020;5:e002096. https://doi.org/10.1136/bmjgh-2019-002096

54. Bougma M, Rossier C. The role of family networks and social capital on women's fertility intentions in Ouagadougou, Burkina Faso. *Journal of Demographic Economics* 2022;88(2):237-55. https://doi.org/10.1017/dem.2021.18

55. Monin JK, Schulz R, Martire LM, *et al.* Spouses' Cardiovascular Reactivity to Their Partners' Suffering. *The Journals of Gerontology: Series B* 2010;65B(2):195-201. https://doi.org/10.1093/geronb/gbp133

56. Ross KM, Rook K, Winczewski L, *et al.* Close Relationships and Health: The Interactive Effect of Positive and Negative Aspects. *Social and Personality Psychology Compass* 2019;13(6). https://doi.org/10.1111/spc3.12468

57. Whisman MA, Gilmour AL, Salinger JM. Marital satisfaction and mortality in the United States adult population. *Health Psychology* 2018;37(11):1041-4. https://doi.org/10.1037/hea0000677

58. Dobrowolska M, Groyecka-Bernard A, Sorokowski P, *et al.* Global Perspective on Marital Satisfaction. *Sustainability* 2020;12(21):8817. https://doi.org/10.3390/su12218817

For peer review only

For peer review only - http://bmjopen.bmj.com/site/about/guidelines.xhtml

771 59. Jackson JB, Miller RB, Oka M, *et al.* Gender Differences in Marital Satisfaction:
772 A Meta-analysis. *Journal of Marriage and Family* 2014;76(1):105-
773 29. https://doi.org/10.1111/jomf.12077
774 60. Mattson RE, Rogge RD, Johnson MD, *et al*. The positive and negative semantic
775 dimensions of relationship satisfaction. *Personal Relationships* 2013;20(2):328-55.
776 https://psycnet.apa.org/doi/10.1111/j.1475-6811.2012.01412.x
777 61. Bosu WK, Aheto JMK, Zucchelli E, *et al.* Determinants of systemic hypertension
778 in older adults in Africa: a systematic review. *BMC Cardiovascular Disorders*.
779 2019;19(1):173. https://doi.org/10.1186/s12872-019-1147-7
780 62. Jaspert FM, Harling G, Sié A, et al. "Association of relationship satisfaction and
781 blood pressure in rural Burkina Faso's elderly population." Abstract presented at
782 the 37th Annual Conference of the European Health Psychology Society (EHPS
783 2023), September 4-8, 2023, Bremen, p.404. https://2023.ehps.net/wp-
784 content/uploads/2023/09/EHPS_2023_Abstracts_UPLOAD.pdf

For peer review only

Section I: Sociodemographic Characteristics

A. Demographic Information, household composition and educational attainment

DEMOGRAPHIC INFORMATION		
D1	Are you the head of this household?	Yes……………………………………1<br>No……………………………………2
D2	What is your relationship to the head of this household?	Partner<br>Parent<br>Grandparent<br>Grandson or Granddaughter<br>Son or daughter<br>Aunt or uncle<br>Niece or nephew<br>Cousin<br>Tenant<br>Friend<br>Other, specify: ________________
D3	Sex. Ask if not clear:	Male……………………………………1<br>Female…………………………………2
D4	How old are you?	__________ years
D5	How many people over the age of 18, including yourself, live in your house-hold?	__________ people
D6	How many people under the age of 18 live in your household?	__________ people
D7	In total, how many years have you spent at school or in full-time study?	__________ years
D8	What is the highest level of education you have completed?	No Formal Schooling…………..1<br>Less than Primary………………2<br>Primary complete………………3<br>Some secondary ………………4<br>Secondary complete…………..5<br>High School complete…………6<br>College/University……………7
D9	What is your marital status?	Never Married………………………1<br>Currently Married…………………2<br>Separated……………………………3<br>Divorced……………………………4<br>Widowed……………………………5<br>Cohabitating…………………………6<br>Refused………………………………7

For peer review only - http://bmjopen.bmj.com/site/about/guidelines.xhtml

B. Socioeconomic Status, Health Expenditures, and Insurance

B1. Household assets

HOUSEHOLD ASSETS		
W1	What is the main source of water drunk by members of your household?	• Eau du robinet dans le logement<br>• Eau du robinet dans la cour/concession<br>• Eau du robinet public/borne fontaine<br>• Eau du robinet chez le voisin<br>• Puits à pompe ou forage<br>• Puits protégés<br>• Puits non protégés<br>• Source protégée<br>• Source non protégée<br>• Eau de pluie<br>• Camion citerne<br>• Charette avec petite citerne/tonneau<br>• Eau de surface<br>• Eau en bouteille<br>• Autre
W2	How long does it take to get the water and return home?	• Time, in minutes
W3	What type of toilet do members of your household usually use?	• Chasse d'eau - à un système d'égout<br>• Chasse d'eau - à un système d'égout - shared<br>• Chasse d'eau - à une fosse septique<br>• Chasse d'eau - à une fosse septique - shared<br>• Chasse d'eau - à des latrines<br>• Fosses/latrines - ventilées ameliorées (VIP)<br>• Fosses/latrines - ventilées ameliorées (VIP) - shared<br>• Fosses/latrines - avec dalles<br>• Fosses/latrines - avec dalles - shared<br>• Fosses/latrines - sans dalles/trou ouvert<br>• Fosses/latrines - sans dalles/trou ouvert - shared<br>• Toilettes à compostage<br>• Toilettes à compostage - shared<br>• Pas de toilette/nature<br>• Autre<br>• Autre - shared
W4	Do you share this toilet with other households?	• Yes/No
W5	What type of fule does your household usually use for cooking?	• Gaz propane liquifié (GPL)<br>• Charbon de bois<br>• Bois<br>• Repas non préparé dans le mémage<br>• Autre
W6	Do you have a separate room that you use as a kitchen?	• Yes/No
W7	How many bedrooms does this household have?	• Number
W8	Does your household own livestock, herds, other farm animals or poultry?	• Yes/No, if N, skip next questions if No

For peer review only - http://bmjopen.bmj.com/site/about/guidelines.xhtml

W9	Which of the following animals does your household own:	• Yes/No
W10	Vaches laitières ou taureaux ?	• Yes/No
W11	Autre bétail ?	• Yes/No
W12	Chevaux, ânes ou mules ?	• Yes/No
W13	Chèvres ?	• Yes/No
W14	Moutons ?	• Yes/No
W15	Poulets ou autre volaille ?	• Yes/No
W16	Does anyone in your household own farmland?	• Yes/No, skip next question if No
W17	How many hectares of farmland do household members have?	• Number
	In this household, doyou have :	
W18	L'électricité ?	• Yes/No
W19	Un poste radio ?	• Yes/No
W20	Une télévision ?	• Yes/No
W21	Un téléphone fixe ?	• Yes/No
W22	Un ordinateur ?	• Yes/No
W23	Un réfrigérateur ?	• Yes/No
W24	Un table?	• Yes/No
W25	Des chaises?	• Yes/No
W26	Une Armoire/bibliothèque?	• Yes/No
	Does a member of this household own :	
W27	Une montre ?	• Yes/No
W28	Un téléphone portable ?	• Yes/No
W29	If yes, how many mobile phones in your household are currently in use?	• Number
W30	How many of the mobile phones currently in use in your household are “smartphones”?	• Number
W31	If so, do you pay for mobile data for any of these phones?	• Yes/No
W32	Une bicyclette ?	• Yes/No
W33	Une motocyclette ou un scooter ?	• Yes/No
W34	Une charrette tirée par un animal ?	• Yes/No
W35	Une voiture ou une camionnette ?	• Yes/No
W37	Does any household member have a bank account ?	• Yes/No

For peer review only - http://bmjopen.bmj.com/site/about/guidelines.xhtml

C. Frailty (For disability, see WHODAS 2.0 above)

FRIED FRAILTY + FALLS		
F1	Have you or those close to you noticed that you have lost weight or become thinner over the last year?	Yes……………………………………………1<br>No…………………………………………….2<br>(IF NO, SKIP TO F3)
F2	How much weight have you lost (in kg)?	1. Provide in kilograms
F3	Does your health now limit the kinds or amounts of vigorous activities you can do, like digging, fetching water from a well or splitting firewood?	Yes, limited a lot…………………..1<br>Yes, limited a little………………..2<br>No…………………………………………..3
F4	How has your overall health changed in the last 12 months?	Much better…………………………..1<br>Better……………………………………..2<br>The same………………………………..3<br>Worse……………………………………..4<br>Much worse…………………………….5
	Please tell me how often you have felt this way during the past week:	
F5	I felt that everything I did was an effort.	Rarely (<1 days)…………………………..1<br>Some of the time (1-2 days)………….2<br>Occasionally (3-4 days)…………………3<br>Most or all of the time (5-7 days)….4
F6	I could not get going.	Rarely (<1 days)…………………………..1<br>Some of the time (1-2 days)………….2<br>Occasionally (3-4 days)…………………3<br>Most or all of the time (5-7 days)….4
	Please consider your activity during a usual week. The rest of the questions will provide extra information on sedentary behaviour.	
F7	On a usual weekday, how many hours did you spend sitting or reclining (excluding sleep)? This may include time sitting on a chair or bench, visiting friends, reading, sitting in church, sitting down to watch television.	____________________Hours<br>____________________Minutes
F8	On a usual weekend day, how many hours did you spend sitting or reclining (excluding sleep)? This may include time sitting on a chair or bench, visiting friends, reading, sitting in church, sitting down to watch television.	____________________Hours<br>____________________Minutes

For peer review only - http://bmjopen.bmj.com/site/about/guidelines.xhtml

PHQ9 (9 questions)		
	Over the past two weeks, how often have you been bothered by any of the following problems?	
P1	Little interest or pleasure in doing things	Not at all......................................0<br>Several days.................................1<br>More than half the days............2<br>Nearly every day.........................3
P2	Feeling down, depressed or hopeless	Not at all......................................0<br>Several days.................................1<br>More than half the days............2<br>Nearly every day.........................3
P3	Trouble falling asleep, staying asleep, or sleeping too much	Not at all......................................0<br>Several days.................................1<br>More than half the days............2<br>Nearly every day.........................3
P4	Feeling tired or having little energy	Not at all......................................0<br>Several days.................................1<br>More than half the days............2<br>Nearly every day.........................3
P5	Poor appetite or overeating	Not at all......................................0<br>Several days.................................1<br>More than half the days............2<br>Nearly every day.........................3
P6	Feeling bad about yourself – or that you are a failure or have let yourself or your family down	Not at all......................................0<br>Several days.................................1<br>More than half the days............2<br>Nearly every day.........................3
P7	Trouble concentrating on things	Not at all......................................0<br>Several days.................................1<br>More than half the days............2<br>Nearly every day.........................3
P8	Moving or speaking so slowly that other people could have noticed. Or the opposite – becoming so fidgety or restless that you have been moving around a lot more than usual	Not at all......................................0<br>Several days.................................1<br>More than half the days............2<br>Nearly every day.........................3
P9	Thoughts that you would be better off dead, or thoughts of hurting yourself in some way	Not at all......................................0<br>Several days.................................1<br>More than half the days............2<br>Nearly every day.........................3

For peer review only

For peer review only - http://bmjopen.bmj.com/site/about/guidelines.xhtml

		1	2	3	4	5	6	7
12	In most ways my life is close to ideal.							
13	I am satisfied with the current state of affairs in my life.							
14	If I could live my life over, I would change almost nothing.							
15	My life *does not* live up to the standards I have for a good life.							
16	I am satisfied with my life.							

E. Relationship satisfaction

IF Currently married or cohabiting: skip to Q2

1. Are you currently in a romantic relationship with someone?<br>For example: currently dating, being engaged, being married	Yes	No

IF No: Skip to next section

	Extremely unhappy	Fairly un-happy	A little unhappy	Happy	Very happy	Extremely happy
2. Please indicate the degree of happiness, all things considered, of your relationship.						
	Not at all	A little	Somewhat	Mostly	Almost completely	Completely
3. How true is the statement: "I have a warm and comfortable relationship with my partner"						
4. How rewarding is your relationship with your partner?						
5. In general, how satisfied are you with your relationship?						

For peer review only - http://bmjopen.bmj.com/site/about/guidelines.xhtml

Section IV: Healthcare Utilization

A. Medical History

MEDICAL HISTORY & STEPS		
MH1a	Have you ever had your blood pressure measured by a doctor or other health worker?	Yes……1<br>No……2<br>Don't Know……3
MH1b	Have you ever been told by a doctor or other health worker that you have raised blood pressure or hypertension?	Yes……1<br>No……2<br>Don't Know……3
MH1c	If yes, have you ever received treatment for raised blood pressure from a doctor or other health worker?	Yes……1<br>No……2<br>Don't Know……3
MH1d	If yes, during the past two weeks, have you been taking your treatment for raised blood pressure with medications prescribed by a doctor or other health worker?	Yes……1<br>No……2<br>Don't Know……3
MH2a	Have you ever had your blood sugar measured by a doctor or other health worker?	Yes……1<br>No……2<br>Don't Know……3
MH2b	Have you ever been told by a doctor or other health worker that you have raised blood sugar or diabetes?	Yes……1<br>No……2<br>Don't Know……3
MH2c	If yes, have you ever received treatment for raised blood sugar or diabetes from a doctor or other health worker?	Yes……1<br>No……2<br>Don't Know……3
MH2d	If yes, during the past two weeks, have you been taking your treatment for raised blood sugar or diabetes with medications prescribed by a doctor or other health worker?	Yes……1<br>No……2<br>Don't Know……3
MH3a	Have you ever had your cholesterol measured by a doctor or other health worker?	Yes……1<br>No……2<br>Don't Know……3
MH3b	Have you ever been told by a doctor, nurse, or other health worker that you have high cholesterol?	Yes……1<br>No……2<br>Don't Know……3
MH3c	If yes, have you ever received treatment for high cholesterol by a doctor or other health worker?	Yes……1<br>No……2<br>Don't Know……3
MH3d	If yes, during the past two weeks, have you been taking your treatment for high cholesterol with medications prescribed by a doctor or other health worker?	Yes……1<br>No……2<br>Don't Know……3
MH4a	Have you ever been told by a doctor or other health doctor that you have a heart disease (heart failure, heart attack, angina etc.)?	Yes……1<br>No……2<br>Don't Know……3

For peer review only - http://bmjopen.bmj.com/site/about/guidelines.xhtml

Section V: Physical Battery, Measurements & Laboratory Studies

Height/Weight		
	Height	
	Weight	
	Waist Circumference	
	Women: are you pregnant?	Yes……………………………………..1<br>No……………………………………….2<br>Don't Know………………………..3

Blood Pressure		
	Blood Pressure Measurement 1	Systolic:<br>Diastolic:
	Blood Pressure Measurement 2	Systolic:<br>Diastolic:
	Blood Pressure Measurement 3	Systolic:<br>Diastolic:

Walk speed		
	Normal walk [REFER TO SHOWCARDS] Did respondent complete the walk at usual pace?	Yes………………………………………………..1<br>No, REFUSED…………………………………2<br>No, cannot walk even with support…3
	Normal walk. Time at 4 meters:	____________________Seconds
	Normal walk: Second attempt	____________________Seconds

Grip strength		
	Have you had any surgery on your left arm, hand or wrist in the last 3 months OR arthritis or pain in your left hand or wrist?	YES………………………………………..1<br>NO 2
	Have you had any surgery on your right arm, hand or wrist in the last 3 months OR arthritis or pain in your right hand or wrist?	Yes...1<br>No 2
	Which hand do you consider your dominant hand? [INSTRUCTIONS: IF A RESPONDENT IS AMBIDEXTROUS, THE HAND THAT IS USED FOR SIGNING/WRITING IS CONSIDERED THE DOMINANT HAND.]	LEFT…………………………………………1<br>RIGHT……………………………………..2<br>USE BOTH THE SAME…………………3
	Did respondent complete the grip strength test?	Yes<br>No, refused<br>No, unable to understand instructions

For peer review only - http://bmjopen.bmj.com/site/about/guidelines.xhtml

Couple Satisfaction Index (CSI-4) - French Version

Avez-vous actuellement un partenaire?

- Oui
- Non

1. Dans l'ensemble, quel est le degré de bonheur de votre relation?
   0) Extrêmement malheureux
   1) Assez malheureux
   2) un peu malheureux
   3) Content
   4) Très heureux
   5) Extrêmement heureux

2. Dans quelle mesure cette affirmation est-elle vraie pour vous: "J'ai une relation chaleureuse et confortable avec mon partenaire"
   0) Pas du tout
   1) Un peu
   2) Modérément
   3) La plupart
   4) Presque totalment
   5) Complètement

3. Dans quelle mesure cette affirmation est-elle vraie pour vous: "J'ai une relation gratifiante avec mon partenaire"?
   0) Pas du tout
   1) Un peu
   2) Modérément
   3) La plupart
   4) Presque totalment
   5) Complètement

4. En général, dans quelle mesure êtes-vous satisfait de votre relation?
   0) Pas du tout
   1) Un peu
   2) Modérément
   3) La plupart
   4) Presque totalment
   5) Complètement

For peer review only

BMJ Open

The Association of Relationship Satisfaction with Blood Pressure: A Cross-Sectional Study of Older Adults in Rural Burkina Faso

Journal:	*BMJ Open*
Manuscript ID	bmjopen-2024-089374.R2
Article Type:	Original research
Date Submitted by the Author:	13-Oct-2024
Complete List of Authors:	Jaspert, Felicitas; Heidelberg University, Institute of Medical Psychology, Medical Faculty and University Hospital<br>Harling, Guy; Africa Health Research Institute, Research Department of Infection and Population Health; University College London, Institute for Global Health<br>Sie, Ali; Centre de Recherche en Sante de Nouna<br>Bountogo, Mamadou; Centre de Recherche en Sante de Nouna<br>Bärnighausen, Till ; University of Heidelberg, Institute of Global Health<br>Ditzen, Beate; Heidelberg University, Institute of Medical Psychology, Medical Faculty and University Hospital<br>Fischer, Melanie; Heidelberg University, Institute of Medical Psychology, Medical Faculty and University Hospital
**Primary Subject Heading**:	Public health
Secondary Subject Heading:	Cardiovascular medicine, Epidemiology, Global health
Keywords:	Cardiovascular Disease, Aged, Blood Pressure, Cross-Sectional Studies, Social Support, PUBLIC HEALTH

SCHOLARONE™
Manuscripts

I, the Submitting Author has the right to grant and does grant on behalf of all authors of the Work (as defined in the below author licence), an exclusive licence and/or a non-exclusive licence for contributions from authors who are: i) UK Crown employees; ii) where BMJ has agreed a CC-BY licence shall apply, and/or iii) in accordance with the terms applicable for US Federal Government officers or employees acting as part of their official duties; on a worldwide, perpetual, irrevocable, royalty-free basis to BMJ Publishing Group Ltd ("BMJ") its licensees and where the relevant Journal is co-owned by BMJ to the co-owners of the Journal, to publish the Work in this journal and any other BMJ products and to exploit all rights, as set out in our licence.

The Submitting Author accepts and understands that any supply made under these terms is made by BMJ to the Submitting Author unless you are acting as an employee on behalf of your employer or a postgraduate student of an affiliated institution which is paying any applicable article publishing charge ("APC") for Open Access articles. Where the Submitting Author wishes to make the Work available on an Open Access basis (and intends to pay the relevant APC), the terms of reuse of such Open Access shall be governed by a Creative Commons licence – details of these licences and which Creative Commons licence will apply to this Work are set out in our licence referred to above.

Other than as permitted in any relevant BMJ Author's Self Archiving Policies, I confirm this Work has not been accepted for publication elsewhere, is not being considered for publication elsewhere and does not duplicate material already published. I confirm all authors consent to publication of this Work and authorise the granting of this licence.

For peer review only

The Association of Relationship Satisfaction with Blood Pressure: A Cross-Sectional Study of Older Adults in Rural Burkina Faso

Felicitas M. Jaspert[1], Guy Harling[2,3,4,5], MD, ScD, Ali Sié[6], MD, PhD, Mamadou Bountogo[6], MD, Till Bärnighausen[7], MD, PhD, ScD, Beate Ditzen[1], PhD and Melanie S. Fischer[1], PhD

[1] Institute of Medical Psychology, Heidelberg University Hospital, Heidelberg University, Germany

[2] Africa Health Research Institute, South Africa

[3] Institute for Global Health, University College London, UK

[4] School of Nursing & Public Health, University of KwaZulu-Natal, South Africa

[5] MRC/Wits Rural Public Health & Health Transitions Research Unit (Agincourt), University of the Witwatersrand, South Africa

[6] Centre de Recherche en Santé de Nouna, Burkina Faso

[7] Heidelberg Institute of Global Health, Heidelberg University, Germany

For peer review only

Author Note

Guy Harling https://orcid.org/0000-0001-6604-491X

Ali Sié https://orcid.org/0000-0002-3959-2520

Mamadou Bountogo https://orcid.org/0000-0001-5010-1617

Till Bärnighausen https://orcid.org/0000-0002-4182-4212

Beate Ditzen https://orcid.org/0000-0001-5853-4572

Melanie S. Fischer https://orcid.org/0000-0002-3907-1549

Melanie S. Fischer is now at the Department of Psychology, Philipps-Universität Marburg, Germany.

Correspondence concerning this article should be addressed to Melanie S. Fischer, Department of Psychology, Philipps-Universität Marburg, Gutenbergstr. 18, 35037 Marburg, Germany. E-mail: melanie.fischer@uni-marburg.de

The corresponding author attests that all listed authors meet authorship criteria and that no others meeting the criteria have been omitted.

For peer review only

ABSTRACT

Objectives

To examine the association between relationship satisfaction and blood pressure (BP) in a low-income setting and to screen for gender moderation in this context. Research conducted in high-income settings suggests that relationship satisfaction is associated with better physical and mental health outcomes.

Design

Cross-sectional study design using cross-sectional questionnaire and physical measurement data. Multiple linear regression models were calculated for systolic and diastolic BP and adjusted for age, gender, demographics/socioeconomics, and other health-related variables. Gender moderation was tested using interaction terms in multivariable analyses.

Setting

Household survey conducted in 2018 in rural northwestern Burkina Faso.

Participants

Final analysis included 2,114 participants aged over 40 who were not pregnant, reported being in a partnership, and had valid BP readings.

Main Outcome Measures

Systolic and diastolic BP levels.

Results

A significant positive association existed between relationship satisfaction (Couples Satisfaction Index-4 score) and systolic BP (B = 0.23, 95% CI (0.02 to 0.45), p = .03) when controlling for demographics/socioeconomics. Nevertheless, this relationship lost statistical significance when additional adjustments were made for health-related variables (B = 0.21, 95%

For peer review only

CI (-0.01 to 0.42), p = .06). There was no significant association of relationship satisfaction and diastolic BP and no evidence of gender moderation.

Conclusion

In contrast to many higher-income settings, we found a positive association between relationship satisfaction and systolic BP in very-low-income rural Burkina Faso. Our results add to the evidence regarding the contextual nature of the association between relationship satisfaction and health, as high relationship satisfaction may not act as a health promotor in this socioeconomic context.

For peer review only

STRENGHTS AND LIMITATIONS OF THIS STUDY

- Population-based Design: The study's large, representative sample of older adults from rural Burkina Faso enhances the generalizability of the findings to similar sociodemographic settings in Sub-Saharan Africa.
- Objective Measurements: The use of standardized questionnaires and physical measurements allows for objective quantification of health parameters, ensuring comparability with other studies.
- High Reliability of the Couples Satisfaction Index-4 (CSI-4): The CSI-4 demonstrated high reliability in the sample, supporting the robustness of the relationship satisfaction measure used.
- Measurement Constraints: The study's reliance on a brief, unidimensional measure of relationship satisfaction (CSI-4) and the use of clinical blood pressure measurements, rather than ambulatory methods, may have limited the ability to capture nuanced variations in relationship satisfaction and its potential associations with blood pressure.
- Cross-sectional Design: The cross-sectional nature of the study limits the ability to infer causation, highlighting the need for longitudinal research to better understand the causal pathways between relationship satisfaction and blood pressure in this context.

For peer review only

INTRODUCTION

Between 2019 and 2050, Sub-Saharan Africa will account for more than half of world population growth with a predicted population rise of one billion people [1]. Meanwhile, life expectancy in Sub-Saharan Africa has steadily improved from 49.4 years in 1990 to 62.9 years in 2019 and is projected to reach 68.5 years by 2050 [1]. Based on population growth and population ageing, the proportion and absolute number of older people in Sub-Saharan Africa is expected to increase. As demographics change, non-communicable diseases, including cardiovascular diseases (CVDs), are on the rise. This is reflected in the 67% increase in total disability-adjusted life years associated with non-communicable diseases in Sub-Saharan Africa between 1990 and 2017 [2]. In 2017, the age-standardized disability-adjusted life year rate due to non-communicable diseases was almost as high as the one due to communicable, maternal, neonatal, and nutritional diseases, the main burden of disease in Sub-Saharan Africa to date [2]. Among the non-communicable diseases causing disability-adjusted life years, CVDs account for the largest share from age 40 onwards, making them particularly impactful on the older population in Sub-Saharan Africa [2]. The systemic role of CVD is emphasized by the fact that in most low- and middle-income countries, the main cause of decline in non-communicable disease mortality is due to the decline in deaths from CVD [3]. It is therefore especially important to target main risk factors for CVD to address the increasing public health burden of non-communicable diseases in Sub-Saharan Africa.

Social relationships are associated with the development and progression of CVD [4,5] and comparable in effect size to established health risk factors [6]. Epidemiological studies have shown that lower social support is associated with a higher mortality rate of affected individuals, especially due to CVDs [e.g. 5,7]. However, to date, studies examining the impact of social support and relationships on health have been conducted mainly in high-income Western countries.

When considering sources of social support, close relationships such as marriage and romantic partnership play a key role. Comparing the effects of different social relationships on

For peer review only

well-being, the quality of marriage is more strongly associated with health than the quality of other family and friendship relationships [8], making spousal relationships a particularly important target in health promotion. Although married people generally exhibit better health than unmarried people, as demonstrated in a meta-analysis by Robles *et al.* [9], the mere fact of being in a partnership or marriage does not in itself necessarily bring benefits, but rather satisfaction and support within such a relationship [10].

Relationship satisfaction is the most important construct studied to represent relationship quality [11], as well as a core construct in basic relationship research [12]. Relationship quality is measured almost exclusively by means of self-reports, thematically built on two main approaches: First, on interpersonal aspects of specific couple interactions such as communication and conflict, and second, on intrapersonal aspects such as a global, subjective evaluation of the relationship. Relationship satisfaction tends to be assigned to the second approach, although items in established measurement scales often overlap with both [11]. In Funk and Rogge's [12] evaluation of measurement scales assessing relationship satisfaction, self-reports focusing on the global assessment of partnership were found to have more information, higher precision, and greater explanatory power with respect to group differences, arguing for more focused measurements of relationship satisfaction using intrapersonal aspects. Since satisfaction measures based on global ratings of relationship satisfaction are less dependent on culturally determined interaction processes (such as communication norms) than measures based on ratings of specific relationship behaviors [13,14], relationship satisfaction is a readily applicable measure for assessing couple relationships in a non-Western context.

In Africa, very little research exists specifically addressing relationship satisfaction or its impact [e.g. 15-17]. Previous research in Western countries demonstrate a significant association between high relationship satisfaction and better cardiovascular health, including lower blood pressure (BP), reduced risk of coronary heart disease, improved heart rate variability, as well as

For peer review only

129 decreased incidence of myocardial infarction [9,18,19]. Nevertheless, research specifically
130 addressing the association of relationship satisfaction with BP remains scarce, with studies
131 reporting mostly small, positive effect sizes, along with significant heterogeneity across studies
132 [9]. To date, several psychophysiological pathways have been proposed to explain the health-
133 promoting effects of high relationship satisfaction, including improved long-term health behaviors,
134 effects on the endocrine and immune systems, and chronic stress buffering effects [9,18,20-22].
135 Central to psychosocial research on CVD is the 'reactivity hypothesis', which posits that stress
136 triggers cardiovascular reactivity, such as elevated BP and heart rate, as well as increases in stress
137 hormone levels and the promotion of inflammation, all of which contribute to the development
138 and progression of CVD [18,22]. Reviews of the literature show, that marital quality significantly
139 affects cardiovascular reactivity, with negative aspects, such as hostility, leading to increased BP
140 and heart rate in marital conflict and positive aspects, such as support, buffering stress reactivity
141 [9,23]. However, the association of relationship satisfaction with cardiovascular health is complex.
142 Particularly in high-quality relationships, stress can be transmitted between partners if one partner
143 has health problems. This potential stress-spillover effect might influence the link between
144 relationship quality and health [23]. As far as we know, our study marks the first investigation into
145 the association between relationship satisfaction and cardiovascular health outcomes in African
146 countries. Concurrent research conducted by Kurniawan *et al.* [24] within the same survey
147 explored the association between relationship satisfaction and metabolic outcomes, expanding
148 upon our inquiry. This represents an initial step in addressing the need for targeted relationship
149 research in this socioeconomic and cultural context.

150 To determine the association of relationship satisfaction with CVD, surrogate endpoints
151 that serve as biomarkers for CVD risk can be used as health endpoints. High BP, a reliable
152 surrogate endpoint and main risk factor for CVD, is most strongly evidenced as a causative factor
153 and highly prevalent in exposure [25,26]. With the shift in world's highest BP values from high-

For peer review only

to low-income countries, Sub-Saharan Africa has become one of the regions with the highest mean BP levels and increasing prevalence of elevated BP [27]. Systolic BP (SBP) ranked first among modifiable risk factors for CVD burden in 2019 [28] and given the high prevalence and impact of elevated BP as the most common risk factor for developing CVD in Sub-Saharan Africa [29], elevated BP is particularly important as a key factor for CVD preventability. In Sub-Saharan Africa, many established risk factors for elevated BP such as age, high body-mass-index (BMI), a sedentary lifestyle, high salt intake, alcohol consumption and smoking have already been confirmed and studied [29,30], while data on the influence of relationship satisfaction on BP remain scarce or even non-existent. As research on composite indices distinguishing cardiovascular health levels and high normal BP in older adults is just beginning to emerge [31], and considering that relationship satisfaction is a potentially modifiable risk factor - such as through relationship education programs [32] - a more refined understanding of these associations could significantly inform targeted interventions.

Apart from the lack of research on health impacts of relationship satisfaction in Sub-Saharan Africa in general, there is an ongoing need to clarify possible moderating effects. Literature on health effects of relationship quality shows evidence of moderation by gender. This moderation is particularly evidenced when cardiovascular surrogate endpoints are used as outcomes and tends to indicate a stronger association of relationship quality and cardiovascular health for women than for men [9]. Nevertheless, most studies conducted separate analyses for gender and did not test directly whether effects were significantly different for men and women, and found mostly small, non-significant differences, where it can be assumed that the sample size used was not sufficient to detect such small effect sizes.

Current study

The current study examined the association between relationship satisfaction and BP, a surrogate endpoint to future cardiovascular risk, in older people of Burkina Faso, a low-income

For peer review only

179 country in Western- Sub-Saharan Africa, and the moderating role of gender in this context using a large, representative sample. This is the first investigation of relationship satisfaction and cardiovascular health in Sub-Saharan Africa focusing on the potential health impact of relationship satisfaction on clinically important, biological, and modifiable CVD risk factors.

183 Marriage practices in Burkina Faso differ significantly from Western norms. Marriage is nearly universal, with women often entering unions before age 18 through arranged marriages, frequently involving significant age disparities. Polygamous marriages are widespread, especially in rural areas, and are commonly associated with lower levels of education [33,34]. While such marriages are generally perceived positively by most Burkinabe women [35] and can offer benefits like emotional support among co-wives, shared child-rearing responsibilities, and economic advantages [36], research indicates that women in polygamous marriages exhibit significantly higher rates of mental health issues and report greater difficulties in family functioning, marital relationships, and overall life satisfaction, with senior wives being more affected than junior wives [33,37,38]. The frequency of divorces initiated by women is increasing, alongside a rise in remarriages [35,39]. When examining relationship satisfaction in this context, it's crucial to consider the unique aspects of relationship formation and marital expectations: While Western marriages often emphasize passionate love and intimacy, Burkinabe unions may prioritize family obligations and economic considerations [39]. Therefore, in arranged marriages, relationship satisfaction may be more closely tied to fulfilling familial duties and achieving social stability rather than personal emotional fulfillment. Nevertheless, an individual's global assessment of their relationship satisfaction, even if based on factors specific to this cultural context, may still play an important role in terms of health outcomes.

201 Additionally, Burkina Faso faces a high prevalence of CVDs, with two CVDs ranking among the top ten causes of death in 2019, and high BP being the fourth major risk factor for mortality and disability [40]. This reinforces the relevance of our research question in this specific

For peer review only

country and unique cultural and health context, potentially suggesting relevant societal and health policy directions.

Based on previous research from Western countries we expected (a) that higher relationship satisfaction would have a protective and thus negative association with SBP and diastolic BP (DBP) levels, and (b) that gender would moderate the relationship, with women showing a stronger association than men.

METHODS

Study area and population

Burkina Faso, a land-locked country of Sub-Saharan Africa, is ranking as one of the lowest-income countries in the world. In 2016, Burkina Faso was listed as the seventh last country in terms of the Human Development Index with 64.8% of the population living in severe multidimensional poverty [41]. The sample used for the study was drawn from the population of the Nouna Health and Demographic Surveillance System (HDSS), located in the Kossi Province, western Burkina Faso. The sample is representative of the population of Burkina Faso with appropriate caution [42]. The HDSS includes the semi-urban city of Nouna and 58 surrounding villages, a study area with a total population of ~ 107,000 people living in around 15,000 households according to the 2015 census. It is surveyed by the national health research center Centre de Recherche en Santé de Nouna (CRSN). The mostly rural study population relies primarily on farming and cattle keeping. Major ethnic groups that make up the multi-ethnic health district are Dafing, Bwaba, Mossi, Peulh and Samo. Islam and the Roman Catholic Church are the most prominent religions represented. Besides the official language French, there are several local dialects, with Dioula being the most widely spoken. Most children receive hardly any education, averaging one year of schooling for women and two years for men [41]. In 2018, less than half of adults (≥ 15 years) could both read and write [42,43]. Marriage is the most common relationship status for adults in Burkina Faso. By the age of 25, 95% of women are married. 58% of the

For peer review only

population in marital unions live monogamously, with polygamy also being widespread, concerning almost 42% of married women [44].

Procedure

The study used cross-sectional data collected as part of the CRSN Heidelberg Aging Study, a household survey conducted in the Nouna HDSS between May and July 2018. The primary objective of the CRSN Heidelberg Aging Study was to conduct a quantitative survey to assess the health status of older adults (≥ 40 years) in rural Burkina Faso. The age-eligible population consisted of approximately 18,000 individuals from the 2015 HDSS census. With the target of 3,000 responses and the assumption of 25% loss due to mortality, mobility, or non-response, 3,998 individuals were selected using a stratified two-stage cluster random sampling approach, explained in more detail elsewhere [45]. Selected individuals were asked for written informed consent, with a witness in case of illiteracy. Data collection was carried out by trained local fieldworkers in the form of a structured questionnaire at the respondents' place of residence. It consisted of an interview on sociodemographic characteristics, physical and mental health, and healthcare utilization, conducted either in French or translated into Dioula. The interviews were individual and either public or private, as chosen by the interviewees. Furthermore, physical measurements and a blood sample were taken by certified research staff. A blank copy of the questionnaire, including relevant sections used in this study, as well as the French version of the Couple Satisfaction Index-4 (CSI-4), is provided as supplementary material. Ethical approval for the CRSN Heidelberg Aging Study was granted by the CRSN Comité d’éthique institutionnelle, the Comité national d’éthique pour la recherche en santé in Ouagadougou, and the Ethics Committee of the University of Heidelberg’s Faculty of Medicine.

Measures

Relationship satisfaction

For peer review only

Considering the sociocultural background as well as the size of the sample, an applicable, brief, reliable, and valid measure of relationship satisfaction was aimed for. Given these requirements, the CSI-4 was selected [12]. The CSI-4 is a psychometrically optimized 4-item self-report scale designed to assess relationship satisfaction in married or cohabiting couples. Items assess relationship satisfaction at a global level (e.g., "In general, how satisfied are you with your relationship?"). The items are typically rated on a 7-point Likert scale for the first item (0 = extremely unhappy; 6 = perfect) and on a 6-point Likert scale for the other three items (0 = not at all; 5 = perfect). The CSI-4 was translated to French for the purpose of the CRSN Heidelberg Aging Study survey and, like other self-report measures in the survey, was administered as an interview by research staff. In order to optimize the measure for administration in the CRSN Heidelberg Aging Study, all items were rated on a 6-point Likert scale, thus making the highest score of the first item 5 = extremely happy. Item scores are summed, with higher total scores reflecting higher levels of overall relationship satisfaction. The CSI-4 total score ranges from 0 to 20 (usually: 0 to 21) and is used as a continuous score. Internal consistency (Cronbach's alpha) in the current sample was $\alpha = .84$. A CSI-4 score of 13.5 or lower has been established as a cutoff to indicate relationship distress [12]. Compared to traditional relationship satisfaction measurements, such as the Marital Adjustment Test and the Dyadic Adjustment Scale, which have already been applied in Burkina Faso [16], the CSI-4, although much shorter, provides a large amount of information, demonstrating a high degree of precision and power [12].

Blood Pressure

Following the recommendations of the WHO STEPS Surveillance Manual [46], BP – SBP and DBP – was measured a total of three times in the left arm after at least 15 minutes of rest in a seated position. Measurements were performed using a portable BP Monitor (Omron Series 7) at 5-minute intervals between the second and third reading. The mean systolic and diastolic values of the last two measurements were calculated to minimize random errors and ensure a more

For peer review only

accurate estimate of BP. The mean values were included in the analyses as continuous variables. Hypertension was defined using the cutoff values set by current guidelines from the American College of Cardiology/American Heart Association (SBP ≥ 130 mmHg or DBP ≥ 80 mmHg) [47].

Covariates

This study accounted for age and gender, relevant demographic and socioeconomic variables as well as health related variables. The selection of covariates was based on their established relevance in epidemiological studies and their known influence on both relationship satisfaction and BP, aiming to control for potential confounding factors. Age was included as a continuous variable, and gender as a binary variable with men as the reference group. Demographic and socioeconomic variables included wealth quintiles, defined using a wealth index based on important housing features and household ownership of durable assets [48], categorized from quintile one (poorest = reference group) to five (richest). Education was coded as a binary variable where 0 represented those who did not complete primary school (less than primary = reference group) and 1 represented those who completed primary school or attained higher levels of education (some = primary completed or more). In the context of Burkina Faso, primary education consists of six years of formal schooling, and completing this level is considered a significant achievement in education attainment, particularly in rural areas where access to education is limited [49]. Because the number of participants who completed any additional schooling past primary school was extremely small (4.1%), we combined education levels into these two categories. Ethnicity was included with the five main ethnic groups coded separately and others as the reference group. Health related variables included BMI, depression measured using the Patient Health Questionnaire for Depression (PHQ-9) and sitting hours during a usual week (Sit-h/week), an indicator of physical activity, all used as continuous variables.

Patient and public involvement statement

302 Patients and/or the public were not involved in the design, or conduct, or reporting, or
303 dissemination plans of this research.

304 **Statistical analyses**

305 To account for potential confounding effects of covariates that might influence both
306 relationship satisfaction and BP values, Pearson's correlation coefficients were calculated for all
307 pairs of continuous variables to assess linear relationships. For categorical variables, mean
308 relationship satisfaction and BP values were computed for each category. Differences between
309 binary categories were assessed using t-tests, while differences across multiple categories were
310 evaluated using one-way ANOVA, providing insight into the statistical significance of group
311 differences.

312 Multiple linear regression models were then applied to test the association between
313 relationship satisfaction and SBP as well as DBP. All models were adjusted for age and gender.
314 The effect of relationship satisfaction on BP was assessed when controlling for demographic and
315 socioeconomic characteristics (Model 1) and when additionally controlling for relevant health
316 related variables (Model 2). The individual conditions for the application of multiple linear
317 regression models, such as the absence of multicollinearity or normal distribution of the residuals,
318 were checked.

319 To investigate whether the association between relationship satisfaction and BP was
320 moderated by gender, an interaction term (gender x CSI-4) was created, and multivariable linear
321 regression models were run for SBP and DBP with all the above covariates and the interaction
322 term. All analyses were performed using R Studio [50].

323 **RESULTS**

324 Of the 3,998 people sampled, 3,026 were willing and able to give informed consent to
325 participate and provided baseline information on sociodemographic variables. The subsample used
326 in our analyses included only respondents who were not pregnant at the time of the survey (N =

For peer review only

2,969), as pregnancy is known to be associated with significant physiological changes including BP levels [51]. In the subsample, 2,254 study participants reported being in a partnership and provided information on relationship satisfaction. Of these, averaged SBP and DBP readings were collected from 2,222 individuals. 101 participants stated having taken BP medication in the last two weeks and were therefore not included in the analyses. Six participants with missing values for BMI and Sit-h/week, as well as one outlier with an extreme value for Sit-h/week (168h = 7x24h) were removed from the sample, giving a total subsample size of N = 2,114 participants. Summary statistics were compiled for the total subsample and for women and men individually and can be found in Table 1. To protect participant confidentiality and prevent identification of individuals in groups with small numbers, the marital statuses of separated, divorced, and widowed were combined into a single category. Descriptive statistics for all study variables are reported in Table 2.

For peer review only

Table 1
Sociodemographic Characteristics of Included Participants (N = 2,114)

	Total (%)	Female (%)	Male (%)
	N = 2,114 (100)	*N* = 826 (39.07)	*N* = 1,288 (60.93)
Age mean (years)	51.7	51	52.1
Marital Status			
Never married	17 (0.80)	7 (0.85)	10 (0.78)
Currently married	1,952 (92.34)	759 (91.89)	1,193 (92.62)
Cohabiting	124 (5.87)	46 (5.57)	78 (6.06)
Seperated, divorced or widowed [a]	21 (0.99)	14 (1.70)	7 (0.55)
Wealth-Quintiles			
Poorest quintile	332 (15.70)	140 (16.95)	192 (14.91)
2nd poorest quintile	442 (20.91)	176 (21.31)	266 (20.65)
Middle quintile	451 (21.33)	168 (20.34)	283 (21.97)
2nd richest quintile	466 (22.04)	185 (22.40)	281 (21.82)
Richest quintile	423 (20.01)	157 (19.01)	266 (20.65)
Ethnicity			
Dafin	834 (39.45)	306 (37.05)	528 (40.99)
Bwama	623 (29.47)	263 (31.84)	360 (27.95)
Mossi	282 (13.34)	105 (12.17)	177 (13.74)
Peulh	186 (8.80)	80 (9.69)	106 (8.23)
Samo	150 (7.10)	57 (6.90)	93 (7.22)
Other	39 (1.84)	15 (1.82)	24 (1.86)
Language			
French	73 (3.45)	5 (0.61)	68 (5.28)
Dioula	1,857 (87.84)	742 (89.83)	1,115 (86.57)
Other	184 (8.71)	79 (9.56)	105 (8.15)
Education			
Less than primary	1,958 (92.62)	797 (96.49)	1,161 (90.14)
Primary school or more	156 (7.38)	29 (3.51)	127 (9.86)

Note. [a] Categories were combined in order to avoid reporting on cells with < 3 participants.

For peer review only

Table 2

Descriptive Characteristics of Included Participants (N = 2,114): Means and Standard Deviations for the Total Sample, Women and Men and T-tests Comparing Gender Group Differences

	Total		Women		Men		T-tests
	N = 2114		*N* = 826		*N* = 1288		
	Mean	SD	Mean	SD	Mean	SD	*t*
CSI-4	12.2	3.6	11.6	3.7	12.5	3.5	5.43***
SBP[a]	125.22	18.22	123.01	19.20	126.63	17.43	4.48***
DBP	80.54	10.98	79.83	10.99	80.99	10.96	2.38*
BMI[b]	22.10	3.90	22.56	4.53	21.80	3.41	-4.41***
PHQ-9	4	3.4	4.3	3.3	3.7	3.4	-4.02***
Sit-h/week	24.1	12.6	24	12.2	24.2	12.8	0.53
	N	%	N	%	N	%	
Hypertensive[c]	1140	53.93	418	50.61	722	56.06	
Stage I[d]	637	30.13	229	27.72	408	31.68	
Stage II[e]	503	23.79	189	22.88	314	24.38	

Note. CSI-4 = Couple Satisfaction Index-4; SBP = Systolic Blood Pressure; DBP = Diastolic Blood Pressure; BMI = Body Mass Index; PHQ-9 = Patient Health Questionnaire for Depression; Sit-h/week = Sitting hours per usual week

.$p < .1$. *$p < .05$. **$p < .01$. ***$p < .001$

[a] BP [mmHg]

[b] BMI [kg/m^2]

[c] SBP ≥130 mmHg or DBP ≥80 mmHg

[d] SBP 130-139 mmHg or DBP 80-89 mmHg

[e] SBP ≥140 mmHg or DBP ≥ 90 mmHg

For peer review only

For peer review only - http://bmjopen.bmj.com/site/about/guidelines.xhtml

The results of the correlation matrix can be found in Table 3, and the statistical comparisons across categories are detailed in Table 4. Relationship satisfaction and SBP were significantly but weakly positively correlated, $r(2112) = .05$, $p = .024$, while relationship satisfaction and DBP were not significantly correlated, $r(2112) = .04$, $p = .056$. Given the significant positive association between BMI, relationship satisfaction, and BP, as well as the higher levels of relationship satisfaction overserved in wealthier quintiles, a post-hoc ANOVA was performed to examine whether higher wealth is associated with a higher BMI, followed by a Tukey-HSD test to identify specific differences between wealth quintiles. The analysis revealed significant differences in BMI between wealth quintiles, with individuals in higher wealth quintiles exhibiting significantly higher BMI scores compared to those in lower quintiles (see supplementary material for mean BMI comparisons by wealth quintiles with ANOVA and Tukey-HSD results). The results of the multiple linear regression analyses are presented in Table 5 (SBP) and Table 6 (DBP).

For peer review only

Table 3

Pearson Correlation Matrix of Continuous Variables

Variables	CSI-4	SBP	DBP	Age	BMI	PHQ-9	Sit-h/week
CSI-4	1						
SBP	0.05*	1					
DBP	0.04.	0.78***	1				
Age	-0.04.	0.21***	0.02	1			
BMI	0.07**	0.12***	0.16***	-0.17***	1		
PHQ-9	-0.15***	0.06**	0.00	0.29***	-0.11***	1	
Sit-h/week	0.00	0.09***	0.04*	0.23***	-0.01	0.17***	1

Note. CSI-4 = Couple Satisfaction Index-4; SBP = Systolic Blood Pressure; DBP = Diastolic Blood Pressure; BMI = Body Mass Index; PHQ-9 = Patient Health Questionnaire for Depression; Sit-h/week = Sitting hours per usual week

.$p < .1$ and *$p < .05$ and **$p < .01$ and ***$p < .001$

353

For peer review only

Table 4

Comparison of Mean Relationship Satisfaction, Systolic Blood Pressure, and Diastolic Blood Pressure by Categorical Variables with T-test and ANOVA Results

Variable[a]	Category	Relationship Satisfaction		Systolic Blood Pressure		Diastolic Blood Pressure	
		Mean	*t*	Mean	*t*	Mean	*t*
Education	Less than primary	12.1	-2.34*	125.06	-1.42	80.31	-3.36***
	Some	12.8		127.22		83.38	
		Mean	F-value	Mean	F-value	Mean	F-value
Ethnicity	Dafin	12.4	5.21***	125.45	2.46*	80.59	1.41
	Bwama	11.7		125.96		80.70	
	Mossi	12		124.06		80.25	
	Peulh	12.9		121.49		79.22	
	Samo	12.1		127.19		80.82	
	Other	12.5		127.08		84.03	
Wealth-Quintiles	Poorest quintile	11.4	10.78***	124.88	1.30	80.00	4.49**
	2nd poorest quintile	11.9		123.78		79.34	
	Middle quintile	11.9		125.05		80.38	
	2nd richest quintile	12.5		126.18		80.55	
	Richest quintile	12.9		126.10		82.36	

Note. [a]Gender differences are reported in Table 2.

For peer review only

Table 5

Results of Regression Models Estimating the Association Between Relationship Satisfaction and Systolic Blood Pressure, Controlling for Demographic and Socioeconomic Characteristics (Model 1) and Additionally Relevant Health Related Variables (Model 2)

	Model 1			Model 2		
	Estimate (*B*)	95% CI	*p*	Estimate (*B*)	95% CI	*p*
CSI-4	0.23	[0.02; 0.45]	.03	0.21	[-0.01; 0.42]	.06
Age (years)	0.42	[0.34; 0.50]	<.001	0.43	[0.34; 0.51]	<.001
Female (vs male)	-2.83	[-4.41; -1.26]	<.001	-3.60	[-5.17; -2.03]	<.001
Household wealth (vs poorest quintile)						
2nd poorest quintile	0.22	[-2.33; 2.77]	.87	-0.09	[-2.61; 2.43]	.95
Middle quintile	1.09	[-1.47; 3.65]	.40	0.27	[-2.28; 2.82]	.84
2nd richest quintile	2.36	[-0.22; 4.94]	.07	1.55	[-1.02; 4.11]	.24
Richest quintile	2.03	[-0.67; 4.73]	.14	-0.29	[-3.04; 2.46]	.83
Education (vs no education)	1.76	[-1.28; 4.81]	.26	1.02	[-2.00; 4.04]	.51
Ethnicity (vs Other ethnicities)						
Bwaba	-1.34	[-7.13; 4.46]	.65	-0.87	[-6.60; 4.86]	.77
Dafin	-2.95	[-8.69; 2.80]	.31	-2.36	[-8.04; 3.32]	.42
Mossi	-4.59	[-10.58; 1.41]	.13	-4.11	[-10.04;1.82]	.17
Peulh	-5.84	[-12.02; 0.35]	.06	-4.08	[-10.22; 2.05]	.19
Semo	-1.98	[-8.26; 4.30]	.54	-1.77	[-7.99; 4.44]	.58
Body mass index				0.75	[0.54; 0.95]	<.001
PHQ-9				0.15	[-0.08; 0.39]	.20
Sitting hours per week				0.05	[-0.01; 0.12]	.08

Note. CSI-4 = Couple Satisfaction Index-4; PHQ-9 = Patient Health Questionnaire for Depression

For peer review only

Table 6

Results of Regression Models Estimating the Association Between Relationship Satisfaction and Diastolic Blood Pressure, Controlling for Demographic and Socioeconomic Characteristics (Model 1) and Additionally Relevant Health Related Variables (Model 2)

	Model 1			Model 2		
	Estimate (*B*)	95% CI	*p*	Estimate (*B*)	95% CI	*p*
CSI-4	0.09	[-0.04; 0.22]	.18	0.07	[-0.06; 0.21]	.28
Age (years)	0.03	[-0.02; 0.08]	.21	0.04	[-0.01; 0.10]	.12
Female (vs male)	-0.89	[-1.87; 0.08]	.07	-1.33	[-2.30; -0.36]	.01
Household wealth (vs poorest quintile)						
2nd poorest quintile	-0.57	[-2.15; 1.00]	.47	-0.77	[-2.33; 0.79]	.34
Middle quintile	0.47	[-1.11; 2.05]	.56	-0.04	[-1.61; 1.54]	.96
2nd richest quintile	0.56	[-1.03; 2.15]	.49	0.06	[-1.52; 1.65]	.94
Richest quintile	1.94	[0.27; 3.61]	.02	0.56	[-1.14; 2.26]	.52
Education (vs no education)	1.93	[0.05; 3.81]	.04	1.48	[-0.39; 3.35]	.12
Ethnicity (vs Other ethnicities)						
Bwaba	-2.15	[-5.73; 1.43]	.24	-1.87	[-5.41; 1.68]	.30
Dafin	-2.62	[-6.17; 0.92]	.15	-2.26	[-5.77; 1.26]	.21
Mossi	-2.94	[-6.64; 0.76]	.12	-2.65	[-6.32; 1.02]	.16
Peulh	-3.63	[-7.45; 0.19]	.06	-2.57	[-6.37; 1.22]	.18
Semo	-2.64	[-6.52; 1.24]	.18	-2.49	[-6.33; 1.36]	.20
Body mass index				0.44	[0.31; 0.57]	<.001
PHQ-9				0.06	[-0.08; 0.21]	.40
Sitting hours per week				0.03	[-0.01; 0.06]	.17

Note. CSI-4 = Couple Satisfaction Index-4; PHQ-9 = Patient Health Questionnaire for Depression

For peer review only

Systolic blood pressure

Relationship satisfaction was significantly and positively (*B* = 0.23; 95% CI (0.02 to 0.45); *p* = .03) associated with SBP in Model 1 which controlled for demographic and socioeconomic variables, indicating that individuals who were more satisfied in their relationship had slightly higher SBP values. When relevant health parameters were added to the model (Model 2), the association between relationship satisfaction and SBP was no longer significant (*B* = 0.21; 95% CI (-0.01 to 0.42); *p* = .06). In both models for SBP, age and gender were significant covariates. Age was positively associated with SBP in Model 1 and 2, and women had lower SBP compared to men. In Model 2, BMI was also significantly and positively associated with SBP. The adjusted R^2 was .06 for Model 1 and .08 for Model 2, indicating that the predictors used in each model jointly explained about 6% and 8% of the variance in SBP.

Diastolic blood pressure

No significant association was found between relationship satisfaction and DBP, neither in Model 1 (*B* = 0.09; 95% CI (-0.04 to 0.22); *p* = .18), which controlled for demographic and socioeconomic variables, nor in Model 2 (*B* = 0.07; 95% CI (-0.06 to 0.21); *p* = .28), when relevant health parameters were added. In Model 1, the wealthiest, represented by wealth quintile five, had significantly higher DBP compared to the poorest, as did the educated compared to the uneducated. In Model 2, significant differences in DBP existed between genders, with women having lower DBP compared to the reference group of men. Also, BMI was significantly and positively associated with DBP. The adjusted R^2 was .01 for Model 1 and .03 for Model 2.

Gender as a moderator

The interaction term (gender x CSI-4) was not statistically significant, neither in the linear regression model predicting SBP (*B* = 0.03; 95% CI (-0.08 to 0.47); *p* = .88), nor in the linear regression model predicting DBP (*B* = 0.01; 95% CI (-0.10 to 0.24); *p* = .92), reflecting

For peer review only

379 that there was no evidence of gender differences in the association of relationship satisfaction
380 with BP.

381 **DISCUSSION**

382 The results of our population-based analysis of older adults in Burkina Faso suggest that
383 relationship satisfaction is not associated with DBP. However, relationship satisfaction was
384 positively associated with SBP, although the association was no longer significant after
385 controlling for additional health-related parameters. The correlation coefficient between
386 relationship satisfaction and SBP, although small, was comparable in magnitude to previously
387 reported, mostly small effect sizes of the association between marital quality and health
388 outcomes (r = .07 to .21) [9] that have important implications at a population level. In our
389 multiple regression model adjusted for demographic and socioeconomic characteristics
390 relationship satisfaction was significantly and positively associated with an increase in SBP of
391 0.23 mmHg per unit change in the CSI-4. The effect size corresponds to roughly half of the
392 SBP-increasing effect of one year of ageing and points into a direction that suggests, contrary
393 to most prior findings [9], that more relationally satisfied individuals in this population have
394 higher SBP levels.

395 Given that the association of relationship satisfaction and SBP was no longer significant
396 after controlling for additional health-related parameters, these findings should be interpreted
397 with caution. BMI and depressive symptoms were significantly correlated with both
398 relationship satisfaction and SBP and could act as confounders in the relationship between the
399 two. In contrast to the negative associations of BMI on relationship satisfaction typically
400 observed in Western populations, BMI demonstrated a positive correlation with relationship
401 satisfaction in our sample, emphasizing the importance of context-specific interpretations and
402 the possibility of inverse associations between relationship satisfaction and a variety of health
403 outcomes [24]. In Burkina Faso, cultural perceptions of body size may differ, potentially
404 viewing higher BMI as a sign of attractiveness and wealth, which could positively influence

For peer review only - http://bmjopen.bmj.com/site/about/guidelines.xhtml

relationship satisfaction [24]. Nevertheless, the observed associations of depressive symptoms, wealth, and education with relationship satisfaction were consistent with patterns observed in Western cultural contexts: depressive symptoms were associated with lower relationship satisfaction, while wealth and education were associated with higher relationship satisfaction, consistent with the often-found association of mental health and socioeconomic stability with relationship quality [52]. Furthermore, the post-hoc ANOVA and Tukey-HSD results examining the relationship between BMI and wealth showed that individuals in higher wealth quintiles had significantly higher BMI scores compared to those in lower quintiles. Given that wealthier individuals also report higher relationship satisfaction and considering the significant positive correlation between BMI and BP in our sample, these findings imply that the link between higher relationship satisfaction and increased BP may be partially mediated by BMI.

However, potential association of relationship satisfaction with *higher* cardiovascular risk warrants further attention, as, in general, relationship satisfaction has been shown to be associated with health benefits. In contrast, the review by Kiecolt-Glaser and Wilson [23] synthesizes empirical data demonstrating health risks associated with high-satisfaction partnerships, particularly in response to a partner's suffering, including elevated BP. In closer and more satisfying relationships, greater emotional interdependence can amplify negative emotions if one partner is suffering and encourage a more compassionate reaction, making one spouse's psychological and physiological issues more impactful on the other. Kiecolt-Glaser and Wilson [23] suggest that this effect is more likely to manifest among older adults with narrowing social networks and long-term shared experiences, resulting in increased vulnerability to these adverse health effects. The paradoxical findings of high relationship satisfaction being a risk factor for health particularly in distressing life situations and presumably more likely for older adults could partially explain the unexpected direction of our results. In Burkina Faso, the high prevalence of chronic diseases [53], mental disorders, and vital threats such as malnutrition and violence [30] create an environment where the adverse

For peer review only

effects of high relationship satisfaction could more readily contribute to negative health outcomes. Given that the family plays a central role in coping with stressors [54], a high relationship satisfaction could exacerbate the negative health impact if the stress arises from spousal caregiving or spousal's suffering [23,55].

While most studies on relationship satisfaction have focused on monogamous relationships, understanding the context of a polygamous setting is essential for interpretating the results. The complex dynamics inherent in polygamous marriages, a marital type likely common in our sample, may lead to a higher prevalence of ambivalent relationships. These relationships are characterized by both high positive (e.g., social support) and high negative (e.g., conflict) aspects and have been linked to cardiovascular risk factors such as greater 24-hour BP and greater coronary artery calcification [56]. According to the review by Ross *et al.* [56], positive and negative aspects of close, ambivalent relationships are interacting in a way that positive aspects might not be health-promoting, while negative aspects could potentially pose even greater risks to health than in low-quality social ties. A high relationship satisfaction, presumably functioning as a positive aspect, could therefore contribute to adverse health effects in the context of this heterogeneous sample. Future research should address both positive and negative aspects of relationship quality separately, in order to obtain more information regarding the association with health outcomes.

Gender differences

The association between relationship satisfaction and BP was not moderated by gender, which is consistent with Kurniawan *et al.* [24], who similarly found no gender differences through the utilization of an interaction term analysis. This aligns with previous findings that found no gender differences in the relationship between marital satisfaction and mortality in a general American population sample that directly tested gender differences [57]. When separate analyses for gender were conducted, Robles *et al.* [9], on the other hand, reported statistically significant, larger associations between marital quality and surrogate cardiovascular endpoints

For peer review only

in women than in men. It was discussed that the small, mostly insignificant gender differences were possibly due to too little power to measure small effect sizes or a smaller proportion of women in the sample. Both aspects, as well as a possible overestimation of relationship satisfaction for women (as explained further below), may have influenced our study's findings as well.

Relationship satisfaction

This study represents one of the first investigations to examine relationship satisfaction in a Burkinabe population. The level of relationship satisfaction in our sample indicates that most participants experienced relationship distress, as about 64% (N = 1,345) of individuals reported CSI-4 scores below the cut-off value of 13.5. However, this is likely a slight overestimation of individuals scoring in the distressed range, given that the maximum possible score on the CSI-4 as implemented in the current study was one point lower compared to the original scale. If the cutoff was lowered by one point to 12.5, still over half of our sample, 53.7% (N = 1136), would be considered distressed in their relationships. Women reported poorer overall relationship satisfaction than men, according to mean CSI-4 scores. This gender difference could be due to sociocultural specific characteristics, especially gender roles and patriarchy [58], given the results of Jackson *et al.*'s [59] meta-analysis indicating that there are no differences in marital satisfaction between men and women in community-based samples.

Methodological Considerations

Methodological considerations must also be taken into account when interpreting our findings. Robles et al. [9] noted a more consistent association between relationship satisfaction and structural markers of cardiovascular disease compared to functional indicators like BP. Although studies using BP as a surrogate marker for cardiovascular risk have mostly found beneficial effects of high relationship satisfaction, the results have not always been uniform, nor has the context in which BP was considered - including BP measurements during interactions with spouses, as the mean from 24-hour (ambulatory BP) or from a one-time series

For peer review only

of BP measurements (clinical BP) [9]. One study, reporting findings in line with our results using a Western sample, is that of Cornelius *et al.* [19], which showed that higher relationship satisfaction was associated with higher 24-hour mean SBP, and not with DBP values. The authors suggest the effects of high relationship satisfaction possibly being time-dependent, reflecting both short-term reactive BP fluctuations and long-term cardiovascular adaptations that may influence outcomes in opposing ways [19]. The variability in BP-related outcomes results across studies may underscore the diverse pathways through which relationship satisfaction might influence cardiovascular health.

Strengths and limitations

A significant strength of the current study is that it is large, population-based and representative of local adults from the age of 40 years onwards who are married or have a partner. Accordingly, our findings are locally valid and may also be transferable to other rural areas in Burkina Faso and other Sub-Saharan African countries. Using both standardized questionnaires and physical measurements, internationally established health parameters could be objectively quantified and are thus comparable to other studies.

Nevertheless, several limitations in our study should be acknowledged. While the CSI-4 was highly reliable in our sample on a statistical level, its translation into the widely spoken local language during interviews makes it susceptible to differences in expression. Additionally, the unidimensional way to assess relationship satisfaction may not fully capture the positive and negative aspects present in the diverse range of relationships within a sample of both polygamous and monogamous unions, potentially limiting the nuanced and accurate representation of relationship satisfaction [60]. Furthermore, the study design did not account for marital type, wife-ordering, or inter-relations, despite the possibility that the sample includes non-cohabiting couples from six villages with fewer than 50 age-eligible participants [45]. Moreover, the household survey setting may have compromised privacy, potentially leading to an overestimation of relationship satisfaction in females, as women, whose social status is

For peer review only

deeply connected to their husband's, often face social pressures to withhold personal feelings that could be interpreted as a challenge to the established social order [33].

The current study used clinical BP as a surrogate marker for cardiovascular risk. Since the association of relationship satisfaction on health outcomes seems to be situation and time dependent, the use of 24-hour BP measurements in the home and everyday environment might be more accurate for determining BP values and may potentially lead to greater power in detecting the association between relationship satisfaction and BP.

Limitations that might have prevented the detection of associations between relationship satisfaction, DBP, and SBP after controlling for health-related factors could stem from the study design: the use of clinical rather than ambulatory BP and a brief screening measure for relationship satisfaction rather than a longer unidimensional (e.g., 16- or 32-item CSI) or two-dimensional measure (distinct positive and negative dimensions), which might be able to capture more nuanced variations in relationship satisfaction. However, another reason could be that other predictors are in fact much stronger drivers for BP in the setting of Burkina Faso or because of possible confounding effects. Lastly, the cross-sectional nature of our study cannot demonstrate causation.

CONCLUSIONS

Our study is the first to examine the direct association of relationship satisfaction and BP in rural Sub-Saharan Africa using a study design that offers initial, valuable insights into the role of relationship satisfaction for cardiovascular risk factors in the context of low-income countries in Sub-Saharan Africa, a sociodemographic and economic setting that has been largely ignored in this research field. Our data suggest that relationship satisfaction contributes marginally to explaining SBP variance compared to established BP drivers, especially age and BMI [61], in Burkina Faso and interestingly in the opposite direction to what has mainly been described in other settings. These findings provide evidence for the importance of the contextuality of the relationship between relationship satisfaction and health. Further research

For peer review only

is needed in the context of low-income Sub-Saharan Africa to better understand the effects of relationship satisfaction in this sociocultural setting and to draw general conclusions about the pathways through which relationship satisfaction might exert influence. To examine the direct effect of relationship satisfaction in Sub-Saharan Africa, future studies should address clinical outcomes such as mortality, as well as other cardiovascular surrogate endpoints such as ambulatory BP, and use measures of relationship satisfaction that can particularly capture the positive and negative aspects of relationships in diverse populations. Furthermore, the moderating role of relationship satisfaction on the stress-health link should be investigated further in the context of low-income countries, as it may yield interesting results regarding relevant sample characteristics such as high-prevalence chronic disease or family roles.

For peer review only

AUTHOR CONTRIBUTIONS

T. Bärnighausen, A. Sié and G. Harling conceived and designed the overall CRSN Heidelberg Aging Study. G. Harling and A. Sié coordinated data collection and preparation. M. Bountogo contributed to data acquisition as coordinator of field data collection. F.M. Jaspert conducted the analysis and wrote the manuscript. M.S. Fischer, B. Ditzen and T. Bärnighausen supervised the analysis and manuscript development. All authors substantively reviewed manuscripts, inputted into revisions, and approved the final manuscript.

In preparing this manuscript, ChatGPT was used to assist with refining text for clarity and accuracy, and DeepL was utilized for translation accuracy; all AI-generated content was reviewed and edited by the authors to ensure accuracy and originality.

In accordance with BMJ Open's guidelines, F.M. Jaspert and M.S. Fischer serve as guarantors for this study, accepting full responsibility for the content and conduct of the research.

ACKNOWLEDGMENTS

We would like to express our gratitude to all the interviewers and data collectors responsible for collecting and executing the field study as well as to the participants for taking part in the survey. Additionally, we would like to thank the attendees of the 37th Annual Conference of the European Health Psychology Society (EHPS) for their interest in our research and the valuable discussions during the poster session. The presented results have already been showcased at the conference, and the feedback received has contributed to the development of this manuscript. We refer to the published abstract by Jaspert *et al*. [62], titled 'Association of relationship satisfaction and blood pressure in rural Burkina Faso's elderly population.', presented at the EHPS in 2023, for further details.

FUNDING

This work was supported by the Alexander von Humboldt Foundation through an Alexander von Humboldt Professorship award to T. Bärnighausen, which is funded by the German Federal

For peer review only

Ministry of Education and Research. G. Harling is supported by a fellowship from the Wellcome Trust and Royal Society (grant number 210479/Z/18/Z). M. Fischer was supported by a grant of the Baden-Wuerttemberg Ministry of Science, Research, and the Arts (Germany) and the European Social Fund. This research was funded in whole, or in part, by the Wellcome Trust (grant number 210479/Z/18/Z). For the purpose of open access, the author has applied a CC BY public copyright license to any Author Accepted Manuscript version arising from this submission. The funders had no role in study design, data collection and analysis, decision to publish, or preparation of the manuscript.

COMPETING INTERESTS

The authors declare no conflict of interest.

DATA SHARING STATEMENT

Data used in this study are not publicly available due to restrictions on data sharing imposed by the consent agreements with participants. The dataset includes entire age cohorts from specific villages, and there is a potential risk of deductive disclosure with sufficient local information. Anonymized data can be obtained from the CRSN Heidelberg Aging Study data controllers upon signing a data use agreement that restricts onward transmission. Researchers interested in replicating the analyses or pursuing collaborative studies using CRSN Heidelberg Aging Study data are encouraged to contact Dr. Guy Harling (g.harling@ucl.ac.uk) for further information and to discuss potential collaborations.

REFLEXIVITY STATEMENT

The manuscript underwent individual review by all co-authors, ensuring constructive engagement with the reflexivity statement. Research partners who conducted on-site data collection and survey implementation collaborated with partners specialized in relationship satisfaction to develop the research question. Given the increasing prevalence of cardiovascular diseases in LMICs, investigating potential risk factors is a priority. While there is no LMIC co-author as first or last author due to the collaborative nature of the research question, which

For peer review only

originated from two German institutes and was conducted as part of a medical doctoral thesis, LMIC co-authors played a significant role in the design and implementation of the large-scale population study as well as in the development of this manuscript. Data sharing is ensured as the study is approved by all parties including LMIC co-authors. Furthermore, an open access publication is aimed for to facilitate publication dissemination. Open access funding from the Wellcome Trust has been granted.

ETHICAL APPROVAL

Ethical approval for this study was granted by the Ethics Commission of the Medical Faculty Heidelberg (S-120/2018), the Burkina Faso Comité d'Ethique pour la Recherche en Santé (CERS) in Ouagadougou (2018-4-045), and the Institutional Ethics Committee (CIE) of the CRSN (2018-04). Prior to participation, oral assent was obtained from all village elders. Additionally, written informed consent was obtained from each participant, and in cases of illiteracy, a literate witness assisted in the consent process.

TRANSPARENCY STATEMENT

The lead authors affirm that this manuscript provides an honest, accurate, and transparent account of the study reported herein. No important aspects of the study have been omitted, and any discrepancies from the original study plan have been duly explained.

For peer review only

613 **REFERENCES**

614 1. United Nations. World Population Prospects 2019: Highlights: United Nations; 2019. https://doi.org/10.18356/13bf5476-en
616 2. Gouda HN, Charlson F, Sorsdahl K, *et al.* Burden of non-communicable diseases in sub-Saharan Africa, 1990–2017: results from the Global Burden of Disease Study 2017. *The Lancet Global Health* 2019;7(10):e1375-e87. https://doi.org/10.1016/S2214-109X(19)30374-2
620 3. Bennett JE, Stevens GA, Mathers CD, *et al.* NCD Countdown 2030: worldwide trends in non-communicable disease mortality and progress towards Sustainable Development Goal target 3.4. *The Lancet* 2018;392(10152):1072-88. https://doi.org/10.1016/S0140-6736(18)31992-5
624 4. Holt-Lunstad J, Smith TB, Layton JB. Social Relationships and Mortality Risk: A Meta-analytic Review. *PLOS Medicine* 2010;7(7):e1000316. https://doi.org/10.1371/journal.pmed.1000316
627 5. Orth-Gomér K, Rosengren A, Wilhelmsen L. Lack of social support and incidence of coronary heart disease in middle-aged Swedish men. *Psychosomatic medicin.* 1993;55(1):37-43. https://doi.org/10.1097/00006842-199301000-00007
630 6. House JS, Landis KR, Umberson D. Social relationships and health. *Science* 1988;241(4865):540-5. https://doi.org/10.1126/science.3399889
632 7. Rutledge T, Reis SE, Olson M, *et al.* Social Networks Are Associated With Lower Mortality Rates Among Women With Suspected Coronary Disease: The National Heart, Lung, and Blood Institute-Sponsored Women's Ischemia Syndrome Evaluation Study. *Psychosomatic Medicine* 2004;66(6):882-8. https://doi.org/10.1097/01.psy.0000145819.94041.52

For peer review only

8. Birditt KS, Antonucci TC. Life sustaining irritations? Relationship quality and mortality in the context of chronic illness. *Social Science & Medicine* 2008;67(8):1291-9. https://doi.org/10.1016/j.socscimed.2008.06.029.

9. Robles TF, Slatcher RB, Trombello JM, *et al.* Marital quality and health: A meta-analytic review. *Psychological Bulletin* 2014;140(1): 140–187. https://doi.org/10.1037/a0031859

10. Holt-Lunstad J, Birmingham W, Jones BQ. Is There Something Unique about Marriage? The Relative Impact of Marital Status, Relationship Quality, and Network Social Support on Ambulatory Blood Pressure and Mental Health. *Annals of Behavioral Medicine* 2008;35(2):239-44. https://doi.org/10.1007/s12160-008-9018-y

11. Fincham FD, Rogge R, Beach SRH. Relationship Satisfaction. In: Vangelisti AL, Perlman D, editors. The Cambridge Handbook of Personal Relationships. Cambridge: Cambridge University Press 2018;p. 422–36. (Cambridge Handbooks in Psychology). https://doi.org/10.1017/9781316417867.033

12. Funk JL, Rogge RD. Testing the ruler with item response theory: Increasing precision of measurement for relationship satisfaction with the Couples Satisfaction Index. *Journal of Family Psychology* 2007;21(4):572-583. https://doi.org/10.1037/0893-3200.21.4.572

13. Fonseca AL, Ye T, Curran M, *et al.* Cultural similarities and differences in relationship goals in intercultural romantic couples. *Journal of Family Issues* 2020;42(4), 813-838. https://doi.org/10.1177/0192513X20929071

14. Heyman RE, Sayers SL, Bellack AS. Global marital satisfaction versus marital adjustment: An empirical comparison of three measures. *Journal of Family Psychology* 1995;8(4):432–446. https://doi.org/10.1037/0893-3200.8.4.432

For peer review only

15. Lesch E, Engelbrecht S-K. Relationship satisfaction and gender differences in a South African farm-worker community. *South African Review of Sociology* 2011;42(1):58-77. https://doi.org/10.1080/21528586.2011.563542

16. Lokubal P, Calvert C, Cousens S, *et al.* Investigating the effect of relationship satisfaction on postpartum women's health-related quality of life in Burkina Faso: a cross-sectional analysis. *BMJ Open* 2021;11(9), e048230. https://doi.org/10.1136/bmjopen-2020-048230

17. Sodeinde KJ, Olu-Abiodun O, Bamdele EF, *et al.* A cross-sectional study of female HIV patient perspective of dyadic adjustment in HIV sero-concordant and sero-discordant couples. *Research Journal of Health Sciences* 2021;*9*(2):133-141. https://doi.org/10.4314/rejhs.v9i2.3

18. Smith TW. Intimate relationships and coronary heart disease: implications for risk, prevention, and patient management. *Current Cardiovascular Reports* 2022;24:761-74. https://doi.org/10.1007/s11886-022-01695-4

19. Cornelius T, Birk JL, Edmondson D, *et al.* Romantic relationship satisfaction and ambulatory blood pressure during social interactions: specificity or spillover effects? *Annals of Behavioral Medicine* 2019;53:223-31. https://doi.org/10.1093/abm/kay030

20. Uchino BN, Way BM. Integrative pathways linking close family ties to health: a neurochemical perspective. *American Psychologist* 2017;72:590-600. https://doi.org/10.1037/amp0000049

21. Birditt KS, Newton N, Hope S. Implications of Marital/Partner Relationship Quality and Perceived Stress for Blood Pressure Among Older Adults. *The Journals of Gerontology: Series B* 2012;69(2):188-98. https://doi.org/10.1093/geronb/gbs123

For peer review only

22. Robles TF, Kiecolt-Glaser JK. The physiology of marriage: pathways to health. *Physiology & Behavior*. 2003;79(3):409-16. https://doi.org/10.1016/s0031-9384(03)00160-4

23. Kiecolt-Glaser JK, Wilson SJ. Lovesick: How Couples' Relationships Influence Health. *Annual Review of Clinical Psychology* 2017;13:421-43. https://doi.org/10.1146%2Fannurev-clinpsy-032816-045111

24. Kurniawan AL, Schretzmann J, Paramastri R, et al. Relationship satisfaction and metabolic health parameters: A cross-sectional study in Burkinabe population of older adults. BMC Public Health 2024;24:827. https://doi.org/10.1186/s12889-024-17998-w

25. Fuchs FD, Whelton PK. High blood pressure and cardiovascular disease. *Hypertension* 2020;*75*(2):285-292. https://doi.org/10.1161/hypertensionaha.119.14240

26. Rasnake CM, Trumbo PR, Heinonen TM. Surrogate endpoints and emerging surrogate endpoints for risk reduction of cardiovascular disease. *Nutrition Reviews* 2008;66(2):76-81. https://doi.org/10.1111/j.1753-4887.2007.00010.x

27. Zhou B, Bentham J, Di Cesare M, *et al.* Worldwide trends in blood pressure from 1975 to 2015: a pooled analysis of 1479 population-based measurement studies with 19·1 million participants. *The Lancet* 2017;389(10064):37-55. https://doi.org/10.1016/S0140-6736(16)31919-5

28. Roth GA, Mensah GA, Johnson CO, *et al.* Global Burden of Cardiovascular Diseases and Risk Factors, 1990–2019: Update From the GBD 2019 Study. *Journal of the American College of Cardiology* 2020;76(25):2982-3021. https://doi.org/10.1016/j.jacc.2020.11.010

For peer review only

29. Hendriks ME, Wit FW, Roos MT, *et al.* Hypertension in sub-Saharan Africa: cross-sectional surveys in four rural and urban communities. *PLoS One* 2012;7(3):e32638. https://doi.org/10.1371/journal.pone.0032638

30. Bos ER, Jamison DT, Bainga F, *et al*. Disease and Mortality in Sub-Saharan Africa: *The World Bank* 2006. 410 p. https://doi.org/10.1596/978-0-8213-6397-3

31. Ren Y, Cheng L, Song Y, *et al.* Ideal cardiovascular health index and high-normal blood pressure in elderly people: evidence based on real-world data. *Scientific Reports*. 2024;14(1):10166. https://doi.org/10.1038/s41598-024-60906-w

32. Markman HJ, Rhoades GK. Relationship Education Research: Current Status and Future Directions. *Journal of Marital and Family Therapy* 2012;38(1):169-200. https://doi.org/10.1111/j.1752-0606.2011.00247.x

33. Bove R, Valeggia C. Polygyny and women's health in sub-Saharan Africa. *Social Science & Medicine*. 2009;68(1):21-9. https://doi.org/10.1016/j.socscimed.2008.09.045

34. Shaiful Bahari I, Norhayati MN, Nik Hazlina NH, *et al.* Psychological impact of polygamous marriage on women and children: a systematic review and meta-analysis. *BMC Pregnancy Childbirth*. 2021;21(1):823. https://doi.org/10.1186/s12884-021-04301-7

35. Guirkinger C, Gross J, Platteau J-P. Are women emancipating? Evidence from marriage, divorce and remarriage in rural Northern Burkina Faso. *World Development* 2021;146:105512. https://doi.org/10.1016/j.worlddev.2021.105512

36. Madhavan S. Best of Friends and Worst of Enemies: Competition and Collaboration in Polygyny. *Ethnology* 2002;41(1):69-84. https://doi.org/10.2307/4153021

For peer review only

37. Shepard LD. The impact of polygamy on women's mental health: a systematic review. *Epidemiology and Psychiatric Sciences* 2013;22(1):47-62. https://doi.org/10.1017/s2045796012000121

38. Al-Krenawi A, Graham JR. A Comparison of Family Functioning, Life and Marital Satisfaction, and Mental Health of Women in Polygamous and Monogamous Marriages. *International Journal of Social Psychiatry* 2006;52(1):5-17. https://doi.org/10.1177/0020764006061245

39. Hewitt B, Churchill B. Chapter 3 - Convergence and difference: Marriage and family life from a cross-cultural perspective. In: Halford WK, van de Vijver F, editors. Cross-Cultural Family Research and Practice: Academic Press; 2020. p. 57-102. http://dx.doi.org/10.1016/B978-0-12-815493-9.00003-X

40. Vos T, Lim SS, Abbafati C, *et al.* Global burden of 369 diseases and injuries in 204 countries and territories, 1990–2019: a systematic analysis for the Global Burden of Disease Study 2019. *The Lancet* 2020;396(10258):1204-22. https://doi.org/10.1016/S0140-6736(20)30925-9

41. United Nations Development Programme. Statistical update 2018: Human development indices and indicators [online]. http://report2017.archive.s3-website-us-east-1.amazonaws.com (accessed 11.09.2023).

42. Sié A, Louis VR, Gbangou A, *et al.* The Health and Demographic Surveillance System (HDSS) in Nouna, Burkina Faso, 1993-2007. *Glob Health Action* 2010;3, 5284. https://doi.org/10.3402/gha.v3i0.5284

43. Macrotrends. *Burkina Faso literacy rate 1975-2024*. [online] https://www.macrotrends.net/countries/BFA/burkina-faso/literacy-rate (accessed 25.03.2024).

44. Institut National de la Statistique et de la Démographie [INSD] et ICF International. Enquête démographique et de santé et à indicateurs multiples du

For peer review only

Burkina Faso 2010. (EDSBF-MICS IV). 2012. https://dhsprogram.com/pubs/pdf/FR256/FR256.pdf

45. Witham MD, Davies JI, Bärnighausen T, *et al.* Frailty and physical performance in the context of extreme poverty: a population-based study of older adults in rural Burkina Faso. *Wellcome Open Research* 2019;4:135. https://doi.org/10.12688/wellcomeopenres.15455.1

46. World Health Organization. Noncommunicable Diseases and Mental Health Cluster. WHO STEPS surveillance manual: the WHO STEPwise approach to chronic disease risk factor surveillance/ Noncommunicable Diseases and Mental Health. *World Health Organization* 2005. https://iris.who.int/handle/10665/43376

47. Whelton PK, Carey RM, Aronow WS, *et al.* 2017 ACC/AHA/AAPA/ABC/ACPM/AGS/APhA/ASH/ASPC/NMA/PCNA Guideline for the Prevention, Detection, Evaluation, and Management of High Blood Pressure in Adults: A Report of the American College of Cardiology/American Heart Association Task Force on Clinical Practice Guidelines. *Hypertension* 2018;71(6):e13-e115. https://doi.org/doi:10.1161/HYP.0000000000000065

48. Rutstein SO, Johnson K. The DHS wealth index. Calverton, Maryland, USA: *ORC Macro* 2004. http://dhsprogram.com/pubs/pdf/CR6/CR6.pdf

49. Werner LK, Jabbarian J, Kagoné M, *et al.* "Because at school, you can become somebody" – The perceived health and economic returns on secondary schooling in rural Burkina Faso. *PLoS One* 2019;14:e0226911. https://doi.org/10.1371/journal.pone.0226911

50. R Core Team. R: A language and environment for statistical computing. R Foundation for Statistical Computing, Vienna, Austria; 2020. https://www.R-project.org/

For peer review only

51. Sanghavi M, Rutherford JD. Cardiovascular Physiology of Pregnancy. *Circulation* 2014;130(12):1003-8. https://doi.org/10.1161/CIRCULATIONAHA.114.009029

52. Rashidi Fakari F, Doulabi MA, Mahmoodi Z. Predict marital satisfaction based on the variables of socioeconomic status (SES) and social support, mediated by mental health, in women of reproductive age: Path analysis model. *Brain and Behavior* 2022;12(3):e2482. https://doi.org/10.1002/brb3.2482

53. Odland ML, Payne C, Witham MD, *et al.* Epidemiology of multimorbidity in conditions of extreme poverty: a population-based study of older adults in rural Burkina Faso. *BMJ Global Health* 2020;5:e002096. https://doi.org/10.1136/bmjgh-2019-002096

54. Bougma M, Rossier C. The role of family networks and social capital on women's fertility intentions in Ouagadougou, Burkina Faso. *Journal of Demographic Economics* 2022;88(2):237-55. https://doi.org/10.1017/dem.2021.18

55. Monin JK, Schulz R, Martire LM, *et al.* Spouses' Cardiovascular Reactivity to Their Partners' Suffering. *The Journals of Gerontology: Series B* 2010;65B(2):195-201. https://doi.org/10.1093/geronb/gbp133

56. Ross KM, Rook K, Winczewski L, *et al.* Close Relationships and Health: The Interactive Effect of Positive and Negative Aspects. *Social and Personality Psychology Compass* 2019;13(6). https://doi.org/10.1111/spc3.12468

57. Whisman MA, Gilmour AL, Salinger JM. Marital satisfaction and mortality in the United States adult population. *Health Psychology* 2018;37(11):1041-4. https://doi.org/10.1037/hea0000677

58. Dobrowolska M, Groyecka-Bernard A, Sorokowski P, *et al.* Global Perspective on Marital Satisfaction. *Sustainability* 2020;12(21):8817. https://doi.org/10.3390/su12218817

For peer review only

59. Jackson JB, Miller RB, Oka M, *et al.* Gender Differences in Marital Satisfaction: A Meta-analysis. *Journal of Marriage and Family* 2014;76(1):105-29. https://doi.org/10.1111/jomf.12077

60. Mattson RE, Rogge RD, Johnson MD, *et al*. The positive and negative semantic dimensions of relationship satisfaction. *Personal Relationships* 2013;20(2):328-55. https://psycnet.apa.org/doi/10.1111/j.1475-6811.2012.01412.x

61. Bosu WK, Aheto JMK, Zucchelli E, *et al.* Determinants of systemic hypertension in older adults in Africa: a systematic review. *BMC Cardiovascular Disorders*. 2019;19(1):173. https://doi.org/10.1186/s12872-019-1147-7

62. Jaspert FM, Harling G, Sié A, et al. "Association of relationship satisfaction and blood pressure in rural Burkina Faso's elderly population." Abstract presented at the 37th Annual Conference of the European Health Psychology Society (EHPS 2023), September 4-8, 2023, Bremen, p.404. https://2023.ehps.net/wp-content/uploads/2023/09/EHPS_2023_Abstracts_UPLOAD.pdf

For peer review only

Table 7

Comparison of Mean Body Mass Index by Wealth Quintiles with ANOVA and Tukey-HSD[1] Results

		Mean	F-value
1st	Poorest quintile	20.6	54.8***
2nd	2nd poorest quintile	21.3	
3rd	Middle quintile	21.9	
4th	2nd richest quintile	22.1	
5th	Richest quintile	24.3	

Note. [1]Post-hoc comparisons revealed that means differed significantly between all pairs of wealth quintiles, except for the 1st and 2nd, 2nd and 3rd, and 3rd and 4th quintiles.

For peer review only

Section I: Sociodemographic Characteristics

A. Demographic Information, household composition and educational attainment

DEMOGRAPHIC INFORMATION		
D1	Are you the head of this household?	Yes……………………………………1<br>No……………………………………2
D2	What is your relationship to the head of this household?	Partner<br>Parent<br>Grandparent<br>Grandson or Granddaughter<br>Son or daughter<br>Aunt or uncle<br>Niece or nephew<br>Cousin<br>Tenant<br>Friend<br>Other, specify: ________________
D3	Sex. Ask if not clear:	Male……………………………………..1<br>Female…………………………………2
D4	How old are you?	__________ years
D5	How many people over the age of 18, including yourself, live in your house-hold?	__________ people
D6	How many people under the age of 18 live in your household?	__________ people
D7	In total, how many years have you spent at school or in full-time study?	__________ years
D8	What is the highest level of education you have completed?	No Formal Schooling…………..1<br>Less than Primary……………….2<br>Primary complete……………….3<br>Some secondary ……………….4<br>Secondary complete…………..5<br>High School complete…………6<br>College/University……………..7
D9	What is your marital status?	Never Married……………………….1<br>Currently Married…………………..2<br>Separated……………………………..3<br>Divorced………………………………4<br>Widowed……………………………..5<br>Cohabitating…………………………6<br>Refused…………………………………7

For peer review only - http://bmjopen.bmj.com/site/about/guidelines.xhtml

B. Socioeconomic Status, Health Expenditures, and Insurance

B1. Household assets

HOUSEHOLD ASSETS		
W1	What is the main source of water drunk by members of your household?	• Eau du robinet dans le logement<br>• Eau du robinet dans la cour/concession<br>• Eau du robinet public/borne fontaine<br>• Eau du robinet chez le voisin<br>• Puits à pompe ou forage<br>• Puits protégés<br>• Puits non protégés<br>• Source protégée<br>• Source non protégée<br>• Eau de pluie<br>• Camion citerne<br>• Charette avec petite citerne/tonneau<br>• Eau de surface<br>• Eau en bouteille<br>• Autre
W2	How long does it take to get the water and return home?	• Time, in minutes
W3	What type of toilet do members of your household usually use?	• Chasse d'eau - à un système d'égout<br>• Chasse d'eau - à un système d'égout - shared<br>• Chasse d'eau - à une fosse septique<br>• Chasse d'eau - à une fosse septique - shared<br>• Chasse d'eau - à des latrines<br>• Fosses/latrines - ventilées ameliorées (VIP)<br>• Fosses/latrines - ventilées ameliorées (VIP) - shared<br>• Fosses/latrines - avec dalles<br>• Fosses/latrines - avec dalles - shared<br>• Fosses/latrines - sans dalles/trou ouvert<br>• Fosses/latrines - sans dalles/trou ouvert - shared<br>• Toilettes à compostage<br>• Toilettes à compostage - shared<br>• Pas de toilette/nature<br>• Autre<br>• Autre - shared
W4	Do you share this toilet with other households?	• Yes/No
W5	What type of fule does your household usually use for cooking?	• Gaz propane liquifié (GPL)<br>• Charbon de bois<br>• Bois<br>• Repas non préparé dans le mémage<br>• Autre
W6	Do you have a separate room that you use as a kitchen?	• Yes/No
W7	How many bedrooms does this household have?	• Number
W8	Does your household own livestock, herds, other farm animals or poultry?	• Yes/No, if N, skip next questions if No

For peer review only

W9	Which of the following animals does your household own:	• Yes/No
W10	Vaches laitières ou taureaux ?	• Yes/No
W11	Autre bétail ?	• Yes/No
W12	Chevaux, ânes ou mules ?	• Yes/No
W13	Chèvres ?	• Yes/No
W14	Moutons ?	• Yes/No
W15	Poulets ou autre volaille ?	• Yes/No
W16	Does anyone in your household own farmland?	• Yes/No, skip next question if No
W17	How many hectares of farmland do household members have?	• Number
	In this household, doyou have :	
W18	L'électricité ?	• Yes/No
W19	Un poste radio ?	• Yes/No
W20	Une télévision ?	• Yes/No
W21	Un téléphone fixe ?	• Yes/No
W22	Un ordinateur ?	• Yes/No
W23	Un réfrigérateur ?	• Yes/No
W24	Un table?	• Yes/No
W25	Des chaises?	• Yes/No
W26	Une Armoire/bibliothèque?	• Yes/No
	Does a member of this household own :	
W27	Une montre ?	• Yes/No
W28	Un téléphone portable ?	• Yes/No
W29	If yes, how many mobile phones in your household are currently in use?	• Number
W30	How many of the mobile phones currently in use in your household are “smartphones”?	• Number
W31	If so, do you pay for mobile data for any of these phones?	• Yes/No
W32	Une bicyclette ?	• Yes/No
W33	Une motocyclette ou un scooter ?	• Yes/No
W34	Une charrette tirée par un animal ?	• Yes/No
W35	Une voiture ou une camionnette ?	• Yes/No
W37	Does any household member have a bank account ?	• Yes/No

For peer review only - http://bmjopen.bmj.com/site/about/guidelines.xhtml

C. Frailty (For disability, see WHODAS 2.0 above)

FRIED FRAILTY + FALLS		
F1	Have you or those close to you noticed that you have lost weight or become thinner over the last year?	Yes……………………………………………1<br>No……………………………………………..2<br>(IF NO, SKIP TO F3)
F2	How much weight have you lost (in kg)?	1. Provide in kilograms
F3	Does your health now limit the kinds or amounts of vigorous activities you can do, like digging, fetching water from a well or splitting firewood?	Yes, limited a lot…………………..1<br>Yes, limited a little………………..2<br>No…………………………………………..3
F4	How has your overall health changed in the last 12 months?	Much better……………………………..1<br>Better……………………………………..2<br>The same…………………………………..3<br>Worse………………………………………..4<br>Much worse……………………………..5
	Please tell me how often you have felt this way during the past week:	
F5	I felt that everything I did was an effort.	Rarely (<1 days)…………………………..1<br>Some of the time (1-2 days)………….2<br>Occasionally (3-4 days)………………..3<br>Most or all of the time (5-7 days)….4
F6	I could not get going.	Rarely (<1 days)…………………………..1<br>Some of the time (1-2 days)………….2<br>Occasionally (3-4 days)………………..3<br>Most or all of the time (5-7 days)….4
	Please consider your activity during a usual week. The rest of the questions will provide extra information on sedentary behaviour.	
F7	On a usual weekday, how many hours did you spend sitting or reclining (excluding sleep)? This may include time sitting on a chair or bench, visiting friends, reading, sitting in church, sitting down to watch television.	______________________Hours<br>______________________Minutes
F8	On a usual weekend day, how many hours did you spend sitting or reclining (excluding sleep)? This may include time sitting on a chair or bench, visiting friends, reading, sitting in church, sitting down to watch television.	______________________Hours<br>______________________Minutes

For peer review only - http://bmjopen.bmj.com/site/about/guidelines.xhtml

PHQ9 (9 questions)		
	Over the past two weeks, how often have you been bothered by any of the following problems?	
P1	Little interest or pleasure in doing things	Not at all.......................................0<br>Several days...................................1<br>More than half the days............2<br>Nearly every day.........................3
P2	Feeling down, depressed or hopeless	Not at all.......................................0<br>Several days...................................1<br>More than half the days............2<br>Nearly every day.........................3
P3	Trouble falling asleep, staying asleep, or sleeping too much	Not at all.......................................0<br>Several days...................................1<br>More than half the days............2<br>Nearly every day.........................3
P4	Feeling tired or having little energy	Not at all.......................................0<br>Several days...................................1<br>More than half the days............2<br>Nearly every day.........................3
P5	Poor appetite or overeating	Not at all.......................................0<br>Several days...................................1<br>More than half the days............2<br>Nearly every day.........................3
P6	Feeling bad about yourself – or that you are a failure or have let yourself or your family down	Not at all.......................................0<br>Several days...................................1<br>More than half the days............2<br>Nearly every day.........................3
P7	Trouble concentrating on things	Not at all.......................................0<br>Several days...................................1<br>More than half the days............2<br>Nearly every day.........................3
P8	Moving or speaking so slowly that other people could have noticed. Or the opposite – becoming so fidgety or restless that you have been moving around a lot more than usual	Not at all.......................................0<br>Several days...................................1<br>More than half the days............2<br>Nearly every day.........................3
P9	Thoughts that you would be better off dead, or thoughts of hurting yourself in some way	Not at all.......................................0<br>Several days...................................1<br>More than half the days............2<br>Nearly every day.........................3

For peer review only - http://bmjopen.bmj.com/site/about/guidelines.xhtml

		1	2	3	4	5	6	7
12	In most ways my life is close to ideal.							
13	I am satisfied with the current state of affairs in my life.							
14	If I could live my life over, I would change almost nothing.							
15	My life *does not* live up to the standards I have for a good life.							
16	I am satisfied with my life.							

E. Relationship satisfaction

IF Currently married or cohabiting: skip to Q2

1. Are you currently in a romantic relationship with someone?<br>For example: currently dating, being engaged, being married	Yes	No

IF No: Skip to next section

	Extremely unhappy	Fairly un-happy	A little unhappy	Happy	Very happy	Extremely happy
2. Please indicate the degree of happiness, all things considered, of your relationship.						
	Not at all	A little	Somewhat	Mostly	Almost completely	Completely
3. How true is the statement: "I have a warm and comfortable relationship with my partner"						
4. How rewarding is your relationship with your partner?						
5. In general, how satisfied are you with your relationship?						

For peer review only - http://bmjopen.bmj.com/site/about/guidelines.xhtml

Section IV: Healthcare Utilization

A. Medical History

MEDICAL HISTORY & STEPS		
MH1a	Have you ever had your blood pressure measured by a doctor or other health worker?	Yes……1<br>No……2<br>Don't Know……3
MH1b	Have you ever been told by a doctor or other health worker that you have raised blood pressure or hypertension?	Yes……1<br>No……2<br>Don't Know……3
MH1c	If yes, have you ever received treatment for raised blood pressure from a doctor or other health worker?	Yes……1<br>No……2<br>Don't Know……3
MH1d	If yes, during the past two weeks, have you been taking your treatment for raised blood pressure with medications prescribed by a doctor or other health worker?	Yes……1<br>No……2<br>Don't Know……3
MH2a	Have you ever had your blood sugar measured by a doctor or other health worker?	Yes……1<br>No……2<br>Don't Know……3
MH2b	Have you ever been told by a doctor or other health worker that you have raised blood sugar or diabetes?	Yes……1<br>No……2<br>Don't Know……3
MH2c	If yes, have you ever received treatment for raised blood sugar or diabetes from a doctor or other health worker?	Yes……1<br>No……2<br>Don't Know……3
MH2d	If yes, during the past two weeks, have you been taking your treatment for raised blood sugar or diabetes with medications prescribed by a doctor or other health worker?	Yes……1<br>No……2<br>Don't Know……3
MH3a	Have you ever had your cholesterol measured by a doctor or other health worker?	Yes……1<br>No……2<br>Don't Know……3
MH3b	Have you ever been told by a doctor, nurse, or other health worker that you have high cholesterol?	Yes……1<br>No……2<br>Don't Know……3
MH3c	If yes, have you ever received treatment for high cholesterol by a doctor or other health worker?	Yes……1<br>No……2<br>Don't Know……3
MH3d	If yes, during the past two weeks, have you been taking your treatment for high cholesterol with medications prescribed by a doctor or other health worker?	Yes……1<br>No……2<br>Don't Know……3
MH4a	Have you ever been told by a doctor or other health doctor that you have a heart disease (heart failure, heart attack, angina etc.)?	Yes……1<br>No……2<br>Don't Know……3

For peer review only - http://bmjopen.bmj.com/site/about/guidelines.xhtml

Section V: Physical Battery, Measurements & Laboratory Studies

Height/Weight		
	Height	
	Weight	
	Waist Circumference	
	Women: are you pregnant?	Yes……………………………………..1<br>No………………………………………..2<br>Don't Know………………………..3

Blood Pressure		
	Blood Pressure Measurement 1	Systolic:<br>Diastolic:
	Blood Pressure Measurement 2	Systolic:<br>Diastolic:
	Blood Pressure Measurement 3	Systolic:<br>Diastolic:

Walk speed		
	Normal walk [REFER TO SHOWCARDS] Did respondent complete the walk at usual pace?	Yes……………………………………………….1<br>No, REFUSED………….…………………..2<br>No, cannot walk even with support…3
	Normal walk. Time at 4 meters:	___________________Seconds
	Normal walk: Second attempt	___________________Seconds

Grip strength		
	Have you had any surgery on your left arm, hand or wrist in the last 3 months OR arthritis or pain in your left hand or wrist?	YES...1<br>NO 2
	Have you had any surgery on your right arm, hand or wrist in the last 3 months OR arthritis or pain in your right hand or wrist?	Yes...1<br>No 2
	Which hand do you consider your dominant hand? [INSTRUCTIONS: IF A RESPONDENT IS AMBIDEXTROUS, THE HAND THAT IS USED FOR SIGNING/WRITING IS CONSIDERED THE DOMINANT HAND.]	LEFT…………………………………………1<br>RIGHT………………………………………2<br>USE BOTH THE SAME…………………3
	Did respondent complete the grip strength test?	Yes<br>No, refused<br>No, unable to understand instructions

For peer review only - http://bmjopen.bmj.com/site/about/guidelines.xhtml

Couple Satisfaction Index (CSI-4) - French Version

Avez-vous actuellement un partenaire?

- Oui
- Non

1. Dans l'ensemble, quel est le degré de bonheur de votre relation?
   0) Extrêmement malheureux
   1) Assez malheureux
   2) un peu malheureux
   3) Content
   4) Très heureux
   5) Extrêmement heureux

2. Dans quelle mesure cette affirmation est-elle vraie pour vous: "J'ai une relation chaleureuse et confortable avec mon partenaire"
   0) Pas du tout
   1) Un peu
   2) Modérément
   3) La plupart
   4) Presque totalment
   5) Complètement

3. Dans quelle mesure cette affirmation est-elle vraie pour vous: "J'ai une relation gratifiante avec mon partenaire"?
   0) Pas du tout
   1) Un peu
   2) Modérément
   3) La plupart
   4) Presque totalment
   5) Complètement

4. En général, dans quelle mesure êtes-vous satisfait de votre relation?
   0) Pas du tout
   1) Un peu
   2) Modérément
   3) La plupart
   4) Presque totalment
   5) Complètement

For peer review only
